# The J Domain Proteins of *Plasmodium knowlesi*, a Zoonotic Malaria Parasite of Humans

**DOI:** 10.3390/ijms252212302

**Published:** 2024-11-16

**Authors:** Michael O. Daniyan, Harpreet Singh, Gregory L. Blatch

**Affiliations:** 1 Department of Pharmacology, Faculty of Pharmacy, Obafemi Awolowo University, Ile-Ife 220005, Nigeria; 2 Department of Bioinformatics, Hans Raj Mahila Maha Vidyalaya, Jalandhar 144008, India; 3 Biomedical Biotechnology Research Unit, Department of Biochemistry, Microbiology and Bioinformatics, Rhodes University, Makhanda 6140, South Africa; 4 Centre for Molecular Medicine and Innovative Therapeutics, Murdoch University, Perth, WA 6150, Australia; 5 The Vice Chancellery, The University of Notre Dame Australia, Fremantle, WA 6959, Australia

**Keywords:** heat shock proteins, HSP40, HSP70, HSP90, J domain protein, molecular chaperones, *Plasmodium knowlesi*, protein folding, zoonotic malaria

## Abstract

*Plasmodium knowlesi* is a zoonotic form of human malaria, the pathology of which is poorly understood. While the J domain protein (JDP) family has been extensively studied in *Plasmodium falciparum*, and shown to contribute to malaria pathology, there is currently very limited information on the *P. knowlesi* JDPs (PkJDPs). This review provides a critical analysis of the literature and publicly available data on PkJDPs. Interestingly, the *P. knowlesi* genome encodes at least 31 PkJDPs, with well over half belonging to the most diverse types which contain only the signature J domain (type IIIs, 19) or a corrupted version of the J domain (type IVs, 2) as evidence of their membership. The more typical PkJDPs containing other domains typical of JDPs in addition to the J domain are much fewer in number (type IIs, 8; type Is, 2). This study indentifies PkJDPs that are potentially involved in: folding of newly synthesized or misfolded proteins within the *P. knowlesi* cytosol (a canonical type I and certain typical type IIs); protein translocation (a type III) and folding (a type II) in the ER; and protein import into mitochondria (a type III). Interestingly, a type II PkJDP is potentially exported to the host cell cytosol where it may recruit human HSP70 for the trafficking and folding of other exported *P. knowlesi* proteins. Experimental studies are required on this fascinating family of proteins, not only to validate their role in the pathology of knowlesi malaria, but also because they represent potential anti-malarial drug targets.

## 1. Introduction

**Figure 1:**
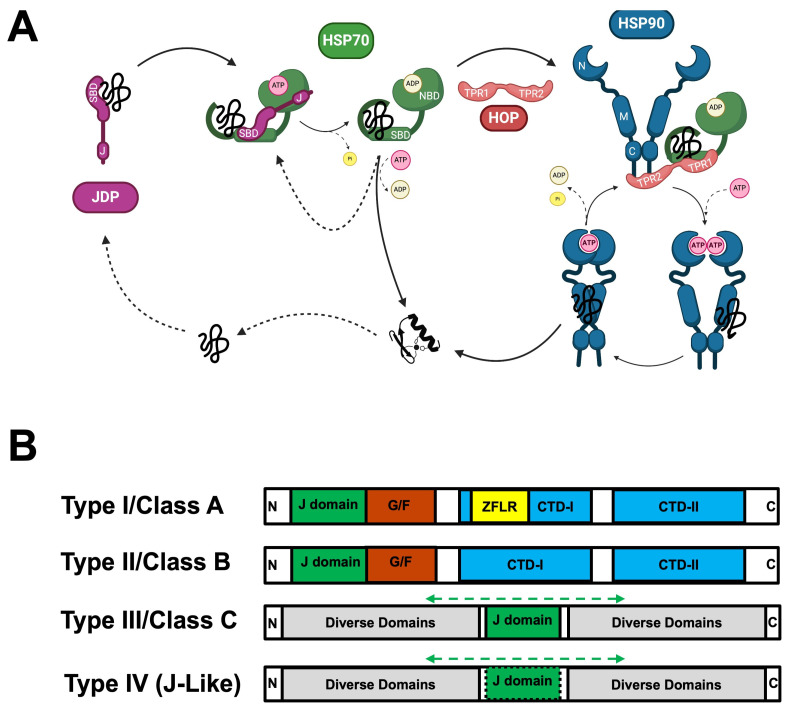
JDPs are a structurally diverse family of proteins that network with the major chaperones, HSP70s and HSP90s. (**A**) Chaperones assist newly synthesized or unfolded/misfolded proteins (black coil) to fold into a functional state (black helix/sheet) [17]. JDPs (purple) recognize a defined range of protein substrates which they target to partner HSP70s (green), thereby stimulating ATP hydrolysis and locking the substrate in the HSP70 SBD [18,19]. Nucleotide exchange triggers the release of substrate to fold, or in the case of certain specialized HSP90 client proteins, HSP70 delivers the substrate to HSP90 (blue) with the assistance of HOP (red), resulting in another ATP-dependent chaperone cycle, before the substrate is released to fold or re-enter the cycle if necessary [20]. Abbreviations used: HSP, heat shock protein; JDP, J domain protein; SBD, substrate-binding domain; J, J domain; NBD, nucleotide-binding domain; N, N-terminal domain; M, middle domain; C, C-terminal domain; HOP, HSP70-HSP90 organizing protein; TPR1 and TPR2, tetratricopeptide repeat domains 1 and 2, respectively. This graphic was created with BioRender (biorender.com). (**B**) The domain organization in the various types or classes of JDPs. The J domain is the signature domain (green) of JDPs, with types I-III J domains containing the highly conserved histidine-proline-aspartic acid (HPD) motif (indicated by the solid box) [21,22,23], and type IV J domains containing a corrupted HPD motif (indicated by the dashed box) [8]. The J domain occurs near the N-terminus of type I and II JDPs, while in type III and IV JDPs it can be found anywhere along the sequence (green dashed double-headed arrow). The other domains are as follows: the glycine and phenylalanine rich region (G/F; brick red); the C-terminal substrate-binding domain I (CTD-I; sky blue) with an embedded zinc-finger-like region (ZFLR; yellow); and the C-terminal substrate-binding domain II (CTD-II; sky blue). The letters N and C indicate the N-terminal and C-terminal ends of the proteins.

While the annual human death toll due to malaria declined steadily over the 2000–2019 period (reaching 576,000 deaths), this trend was reversed over the 2020–2022 period (with annual deaths increasing to over 600,000) (World Malaria Report 2023, World Health Organization, WHO). Malaria in humans is caused by five species of apicomplexan parasites belonging to the genus *Plasmodium*, namely *Plasmodium falciparum*, *Plasmodium vivax*, *Plasmodium malariae*, *Plasmodium ovale* and *Plasmodium knowlesi*. *P. falciparum* causes the most severe form of malaria and is arguably the most well characterized species, while there are considerable knowledge gaps in the pathobiology of *P. knowlesi*. *P. knowlesi* is a zoonotic form of human malaria with simian origins [1]. The natural hosts of knowlesi malaria are predominantly leaf monkeys and macaques found across Southeast Asia. While the earliest reports (1960s and 1970s) of natural infections of humans with *P. knowlesi* were isolated cases of travelers passing through Southeast Asia, by the early 2000s local transmission was highly prevalent in this region leading to the export of knowlesi malaria beyond its borders [2]. While there has been near-elimination of human-only *Plasmodium* species in Malaysia, *P. knowlesi* is now the major cause of human malaria deaths in this area [3]. The existence of this zoonotic form of human malaria complicates global strategies towards the eradication of malaria, and hence there is an urgent need to bridge the knowledge gap on the epidemiology, biology and pathology of knowlesi malaria. In this article we address this knowledge gap by critically analyzing the literature on a key family of proteins known to contribute to the pathology of malaria, the heat shock protein 40s (HSP40s) also referred to as J domain proteins (JDPs) (Figure 1).

## 2. JDPs Functionalize Chaperone Machinery and Are Structurally Diverse

Relative to other human malaria parasites, the *P. falciparum* JDPs (PfJDPs) have been extensively studied, and hence there are a number of reviews covering their properties: evolution and categorization as molecular chaperones [4,5]; membership of the exportome [6]; biology and pathology [7,8,9]; and potential as drug targets [10,11,12]. In contrast, there appear to be no studies focused on the characterization of *P. knowlesi* JDPs (PkJDPs), and very few reviews that mention them [13]. However, with increasing attention on *P. knowlesi* as a causative agent of zoonotic malaria in humans, information is starting to emerge on PkJDPs embedded within broader studies, especially genomics [14] and proteomics analyses [15,16]. 

JDPs are co-chaperones of the major molecular chaperones, heat shock protein 70 (HSP70) and heat shock protein 90 (HSP90), and together they form a network that is critically important for ensuring cellular protein integrity and homeostasis (proteostasis) (Figure 1A), particularly under stressful conditions, including disease states [24,25]. Not surprisingly, the JDP-HSP70-HSP90 chaperone machinery of malaria parasites, especially in *P. falciparum* (PfJDPs, PfHSP70s and PfHSP90s), has been found to play an essential role in malaria parasite growth, survival, differentiation and pathogenesis [26]. JDPs are specialist chaperones that functionalize the generalist chaperones, HSP70 and HSP90, by targeting specific substrates to them [17] (Figure 1A). The evidence indicates that JDPs work most closely with HSP70 (Figure 1A), delivering substrate and simultaneously stimulating the HSP70 ATPase activity thereby enabling substrate capture [18,19]. Interestingly, while the co-chaperone HSP70/HSP90 organizing protein (HOP) has been shown to be important in eukaryotic systems for the transfer of specialized substrates from HSP70 to HSP90 [20] (Figure 1A), there is also evidence that certain JDPs can triage with HSP70 and HSP90 to ensure such substrate transfer [27]. Consequently, there are typically many more JDPs than HSP70s and HSP90s, with each JDP responsible for channeling a range of protein substrates to the major chaperones for subsequent assisted folding and/or functional activation [28]. The *P. falciparum* chaperone machinery is a good example, where the PfJDPs (49 members) [8] far outnumber the PfHSP70s (six members) and PfHSP90s (four members) [5], with 18 PfJDPs predicted or shown to be exported [6], and one PfHSP70 (PfHSP70-x) shown to be exported [29,30]. The exported PfJDPs are a major component of the exportome in *P. falciparum*-infected erythrocytes, and there is substantial evidence that certain PfJDPs interact with both exported PfHSP70-x and human HSP70, and are involved in the trafficking of virulence factors (e.g., *P. falciparum* erythrocyte membrane protein 1, PfEMP1), and in ensuring the functional integrity of complexes of human and parasite proteins involved in pathology (e.g., cytoskeletal, membrane and knob complexes) [31,32,33,34].

As their name suggests, the signature domain of JDPs is the J domain, which is essential for functional interaction with HSP70 [18,19]. The J domain is a highly dynamic structure that becomes conformationally constrained as it binds to HSP70, and consists of four helices (I–IV), with anti-parallel helices II and III separated by a loop region containing a highly conserved and catalytically important histidine-proline-aspartic acid (HPD) motif [35]. The JDP family is structurally diverse, with the majority containing just a J domain as evidence of their membership (type III or class C JDPs) [21,23] (Figure 1B). While type III JDPs do not appear to associate directly with unfolded or misfolded protein substrates, they play an important role in ensuring HSP70 is activated appropriately in space and time to carry out its chaperone function [24,25,28]. 

JDPs that are capable of binding to protein substrates for delivery to HSP70 contain a number of other domains, with the canonical members (type I or class A) containing four additional domains (a glycine and phenylalanine (G/F) rich region; a C-terminal substrate-binding domain I, CTD-I, with an embedded zinc-finger-like region, ZFLR; and a C-terminal substrate-binding domain II, CTD-II), and other members (type II or class B) containing all these additional domains except for the ZFLR [21,23] (Figure 1B). Interestingly, an unusual and relatively uncharacterized type of JDP has been discovered (type IV) which has a J domain with a corrupted HPD motif [8] (Figure 1B).

## 3. The PkJDP Family Has Types I to IV and Potential Exported Members

The PkJDPs, *P. knowlesi* HSP70s (PkHSP70s), *P. knowlesi* HSP90s (PkHSP90s) and *P. knowlesi* HOP (PkHOP) were identified using publicly available *P. knowlesi* genomic and transcriptomic sequence information on the National Centre for Biotechnology Information website (NCBI; ncbi.nlm.nih.gov; accessed on 15 July 2024), and the *Plasmodium* Informatics Resources website (PlasmoDB; plasmodb.org/plasmo/app; accessed on 15 August 2024) (Table 1 and Table S1). The PkJDPs, PkHSP70s, PkHSP90s and PkHOP were identified by searching for matching sequences when PfJDPs, PfHSP70s, PfHSP90s and PfHOP were entered as query sequences in the Basic Local Alignment Search Tool (BLAST; blast.ncbi.nlm.nih.gov/Blast.cgi; accessed on 15 July 2024). The *P. knowlesi* PKNH reference genome [36] with recent updates [14] were the primary source of genome sequence and gene annotation information used in the identification of the members of these chaperone families (Table 1). There are only a limited number of comprehensive proteomics studies conducted on *P. knowlesi* within the context of its natural host, and even fewer associated with infection of humans [16]. *P. knowlesi* has been adapted for in vitro culture within human erythrocytes, establishing a model system to study the biology and pathology of zoonotic knowlesi malaria [37]. Using this model system, proteomics studies of *P. knowlesi*-infected human erythrocytes are starting to emerge, providing valuable functional genomics information about this key phase of the life cycle in the human host [15]. Useful information on the expression and localization of *P. knowlesi* chaperones was captured from proteomics analyses of sub-cellular fractions of *P. knowlesi*-infected human erythrocytes supplementary materials of [15] (Table 1 and Table S1).

### 3.1. There Is a Canonical and a Non-Canonical Type I PkJDP

The *P. knowlesi* genome encodes at least 31 PkJDPs (Table 1 and Table S1), five PkHSP70s, four PkHSP90s and one PkHOP (Table S1). Not surprisingly, over half of the PkJDP members are type III (19), with a number of type II (8), and a few type I (2) and IV (2) members (Table 1). The type I PkJDPs, PKNH_0424600 and PKNH_0307500, are homologs of the previously characterized canonical (PfHSP40 [38]) and non-canonical (Pfj1 [39,40,41]) type I PfJDPs, respectively. PfHSP40 was shown to be a co-chaperone of the major cytosolic and nuclear PfHSP70, PfHSP70-1, exhibiting similar localization patterns and upregulation under heat shock [8]. Therefore, PKNH_0424600 is most likely the canonical parasite-resident PkJDP associating with the canonical parasite-resident PkHSP70 (PKNH_1312700; Table 1 and Table S1). While Pfj1 appears to contain a potential mitochondrial import signal (RRKVCS), it has been shown to be localized to the apicoplast [41]. Furthermore, Pfj1 appears to be a functional JDP and it can stimulate the ATPase activity of HSP70 [40]. Although PKNH_0307500 is homologous to Pfj1 (Figure S1B; Table 1; 83% sequence identity), the N-terminal region upstream from the J domain has very low sequence identity to Pfj1 and no obvious motifs associated with localization (i.e., no organellar import signal sequences or PEXEL motif for export). 

### 3.2. The Type II PkJDPs Include a Potential Exported Member

**Figure 2:**
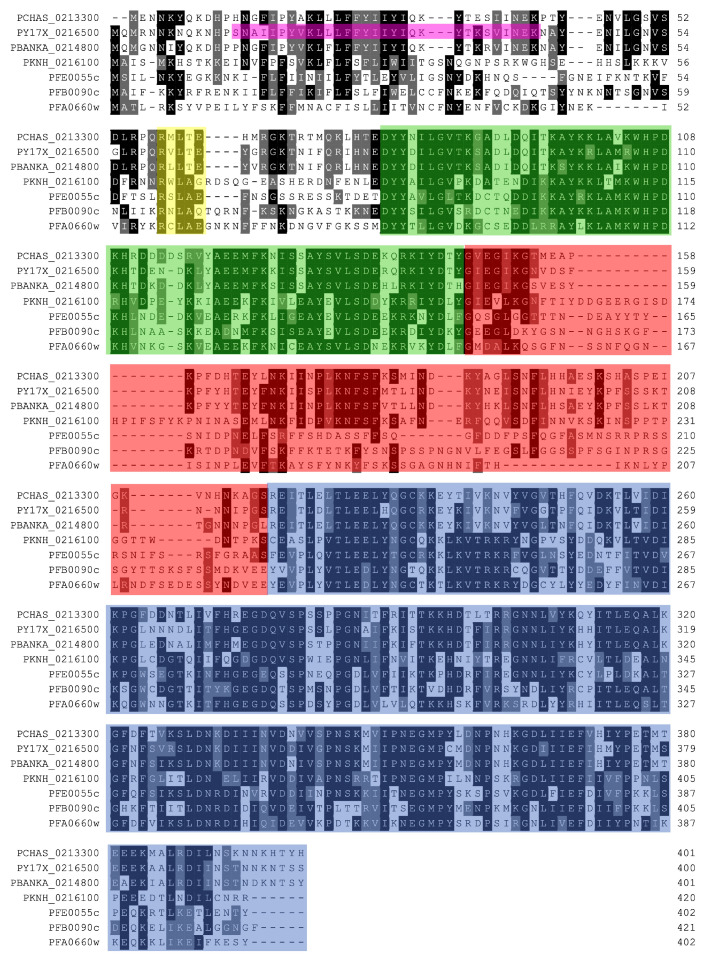
The *P. knowlesi* type II JDP, PKNH_0216100, is predicted to be exported into the human host cell cytosol and is homologous to other plasmodial type II JDPs known or predicted to be exported. The proteins in the multiple sequence alignment are defined by their PlasmoDB accession number in the first column: *P. chabaudi* type II JDP, PCHAS_0213300; *P. yoelii* type II JDP, PY17X_0216500; *P. berghei* type II JDP, PBANKA_0214800; *P. knowlesi* type II JDP, PKNH_0216100; *P. falciparum* type II JDPs, PFE0055c/PF3D7_0501100, PFB0090c/PF3D7_0201800, and PFA0066w/PF3D7_0113700. The protein sequences for all JDPs are listed in the Supplementary Materials (List S1). Colored in black are identical amino acids (in at least 50% of the aligned sequences), colored in light gray are similar amino acids (in at least 50% of the aligned sequences), and colored in white are the amino acids with no identity or similarity. The default categories for similar amino acids were applied to the multiple sequence alignment (ILV, FWY, KRH, DE, GAS, P, C and TNQM). In *P. yoelli*, the *Plasmodium* Lipophilic And Secondary structure Mediated Export Domain (PLASMED) motif (purple shading) has been identified as required for export [42]. In *P. falciparum*, most exported proteins contain a *Plasmodium* export element (PEXEL, also known as the host-targeting signal), a pentameric motif (RxLxE/Q/D; yellow shading) generally located downstream of a recessed signal sequence [43,44]. During export, the PEXEL sequence is recognized and cleaved between the third and fourth residues by Plasmepsin V. The J domain (green shading), the G/F-rich region (brick red shading), and the C-Terminal Domain (CTD containing CTD-I and II; blue shading) containing the substrate binding site, are all indicated by colored shading. The numbers in the last column are the residue positions within the full-length sequence. The alignments were created using Clustal Omega [45] and rendered with box shading using Multiple Align Show [46].

Almost all the type II PkJDPs appear to be homologs of at least one member of the type II PfJPDs (Table 1 and Table S1), with phylogenetic analyses (Figure S1A,B) and a multiple sequence alignment (Figure 2) indicating that one PkJDP (PKNH_0216100) was potentially homologous to a number of type II PfJDPs known or predicted to be exported.

**Figure 3:**
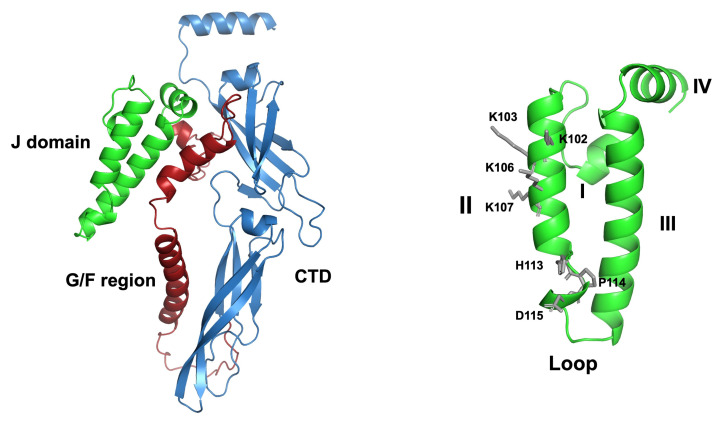
Predicted three-dimensional (3D) structure of PKNH_0216100. The 3D structures of the near-full-length protein, J domain (green)-glycine/phenylalanine (G/F)-rich region (brick red)-C-terminal-domain (CTD; sky blue) (left-hand side), and the J domain alone (green; right-hand side) were determined using the AlphaFold 3 Server (alphafoldserver.com [47]). The predicted template modeling (pTM) scores were above the threshold (0.5) for the predicted structures to be considered potentially similar to the true structure. For the J domain structure, helicies I–IV and the Loop region are labeled, and conserved residues previously identified in other JDPs as important for functional interaction with HSP70 [19,22,35,48,49,50,51,52], have been highlighted as sticks (gray); the highly conserved basic residues on helix II (K102, K103, K106 and K107) and the invariant histidine-proline-aspartate (HPD) motif in the Loop region. The protein sequence for PKNH_0216100 is listed in the Supplementary Materials (List S1). The predicted structures were rendered graphically using PyMol 3.0.4 (PyMOL Molecular Graphics System, Version 3.0 Schrödinger, LLC, New York, NY, USA).

Interestingly, a sub-cellular proteomics study of *P. knowlesi*-infected human erythrocytes found PKNH_0216100 within host cell cystosol (HCC) and vesicle fractions, suggesting that it was potentially exported [15]. Sequence analysis of PKNH_0216100 revealed that it contained a *Plasmodium* export element (PEXEL; [43,44]) motif at the N-terminus just upstream from the J domain (Figure 2). Furthermore, a multiple sequence alignment (Figure 2) revealed that PKNH_0216100 was similar in sequence and domain organization to plasmodial type II JDPs known (PFE0055c and PFA0066w [32]; PFB0090c [31]; and PY17X_0216500 [42]) or predicted to be exported (the multiple sequence alignment in Figure 2 identified putative PEXEL motifs at the N-terminus of PCHAS_0213300 and PBANKA_0214800). While PKNH_0216100 has a PEXEL motif, it does not appear to have a *Plasmodium* Lipophilic And Secondary structure Mediated Export Domain (PLASMED) motif, previously identified in exported proteins of *P. yoelli*, including the type II JDP PY17X_0216500 [42] (Figure 2). PKNH_0216100 is a typical type II JDP with respect to the presence of domains beyond the J domain, containing a relatively lengthy G/F region which is also rich in serine residues, and a C-terminal domain involved in substrate binding (CTD containing CTD-I and II sub-domains) (Figure 2 and Figure 3). 

Furthermore, the J domain of PKNH_0216100 contains residues that are conserved across prokaryotic and eukaryotic JDPs and found to be important for functional interaction with HSP70s [19,22,35,48,49,50,51,52]. For PKNH_0216100, these are the conserved basic residues on helix II (K102, K103, K106 and K107) and the invariant HPD motif (residues 113–115) in the loop region (Figure 3). 

**Figure 4:**
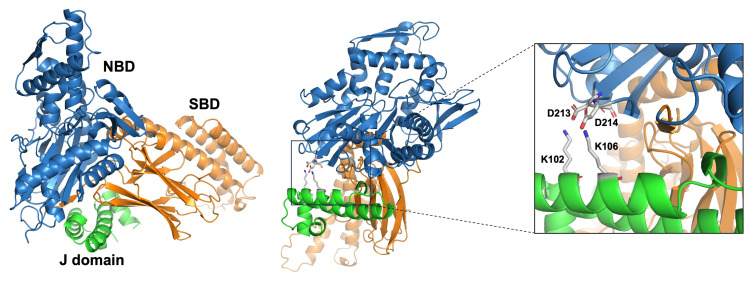
Predicted 3D structure of the complex of the J domain of PKNH_0216100 and human HSP70 (HSPA1A). The complex was rendered in two different orientations, to show the J domain (green) bound to human HSP70 (N-terminal nucleotide-binding domain, NBD, sky blue; C-terminal substrate-binding domain, SBD, orange), with the J domain antiparallel helices II and III front-on (left-hand side) and side-on (middle and right-hand side). The structure predicted that two conserved basic residues of helix II (K102 and K106) and two conserved acidic residues on the underside cleft of human HSP70 NBD (D213 and D214) projected into the binding interface of the complex. These key residues are shown as sticks colored by element type (middle and right-hand side). The two basic residues of helix II are equivalent to previously identified conserved JDP residues (see the legend of Figure 3) that make key contacts with conserved acidic residues on the underside cleft of the NBD of HSP70s (acidic residues topologically equivalent to D213 and D214 of human HSP70 [57,58,59]). The predicted 3D complex was determined using the AlphaFold 3 Server (alphafoldserver.com; [47]). The interface predicted template modeling (ipTM) score was above the threshold (0.8) for the predicted complex to be considered potentially similar to the true structure. The protein sequences for PKNH_0216100 and human HSP70 (HSPA1A) are listed in the Supplementary Materials (List S1). The complex was graphically rendered using PyMol 3.0.4 (PyMOL Molecular Graphics System, Version 3.0 Schrödinger, LLC).

There is compelling evidence that two of the exported type II PfJDPs, PFA0660w and PFE0055c, can associate with the exported PfHSP70-x. They can functionally engage PfHSP70-x as co-chaperones (PFA0660w [53]; PFE0055c [52]), in complexes involved in the trafficking of virulence factors (e.g., PfEMP1) through the infected erythrocyte cytosol (“J dots”; PFE0055c and PFA0660w [54,55,56]), and in complexes within the parasitophorous vacuole (PV) potentially involved in trafficking of exported proteins (PFE0055c [32]). Importantly, there is also evidence for the interaction of PFE0055c [32] and PFA0660w [33] with human HSP70. Given that PKNH_0216100 is predicted to be exported, and there appears to be no exported PkHSP70, it is plausible that it functionally interacts with host HSP70 in the trafficking and folding of exported *P. knowlesi* proteins. Within the context of zoonotic human malaria, the possible physical interaction of PKNH_0216100 with human HSP70 (HSPA1A) was explored using in silico analyses (Figure 4). 

The J domain of PKNH_0216100 was predicted to form a complex with full-length human HSP70, binding to the underside cleft of the N-terminal nucleotide-binding domain (NBD; also referred to as the ATPase domain) (Figure 4). Inspection of this complex revealed that two of the conserved basic residues of the J domain helix II (K102 and K106) and two conserved acidic residues of the NBD (D213 and D214) projected into the binding interface (Figure 4). As mentioned previously, the two basic residues of helix II are equivalent to previously identified conserved JDP residues that make key contacts with conserved acidic residues on the underside cleft of the NBD of HSP70s; acidic residues topologically equivalent to D213 and D214 of human HSP70 [57,58,59]. Contact analysis of the binding interface of the complex indicated that one of the conserved J domain helix II residues (K106) potentially formed a strong hydrogen bond with one of the conserved acidic residues of the human HSP70 NBD (D213) (Figure S2).

As for PKNH_0216100, proteomics-based localization studies suggested that the type II PfJDP, PKNH_1246700, might be localized to vesicles or the HCC (Table 1); however, based on the localization of its homologs it was overall deemed parasite-resident [15]. Analysis of the publicly available amino acid sequence of PKNH_1246700 indicated that it was potentially incomplete, lacking helices I and II of the J domain at the N-terminus of the protein (List S1 and Table S1). An alternative start codon was identified upstream of the annotated start codon on the genome sequence, and translation of the coding region revealed the potential amino acid sequence for helices I and II of the J domain of PKNH_1246700 (List S1; Figures S3 and S4). Phylogenetic analyses of this more complete sequence of PKNH_1246700 revealed that it was homologous (Figure S1B; 65% sequence identity) to a parasite-resident type II PfJDP, PFB0595w, which was reported to be localized to the cytosol, upregulated under heat shock, and potentially a co-chaperone of PfHSP70-1 [60] (Table 1 and Table S1). However, while PKNH_1246700 may be a homolog of PFB0595w, another type II PkJDP, PKNH_0407900, also appears to be a homolog of this type II PfJDP (Figure S1B; Table 1; 88% sequence identity). Indeed, the phylogenetic analyses indicated that PKNH_1246700 and PKNH_0407900 were in a common cluster with PFB0595w (Figure S1B). Hence, both of these type II PkJDPs would be predicted to associate with the canonical parasite-resident PkHSP70 (PKNH_1312700; Table 1 and Table S1). PKNH_1311500 is homologous to a type II PfJDP (Pfj4) which was found to associate with PfHSP70-1, and similarly to PfHSP70-1 to localize to the cytosol and the nucleus and to be upregulated under heat shock [61] (Figure S1B; Table 1). Therefore, similarly to the canonical type I PkJDP (PKNH_0424600), the type II PkJDPs, PKNH_1246700, PKNH_0407900, and PKNH_1311500, would be likely to associate with the canonical parasite-resident PkHSP70 (PKNH_1312700; Table S1). The type II PkJDP, PKNH_1120300, is homologous to a type II PfJDP (PFF1415c) shown to be localized to the PV [62] (Figure S1B; Table 1). At least one type II PkJDP, PKNH_0906300, is potentially localized to the endoplasmic reticulum (ER). It is homologous to the ER-localized type II PfJDP, Pfj2 [63] (Figure S1B; Table 1), and similarly to Pfj2 it has a potential C-terminal ER-retention motif (TDEL). 

### 3.3. Type III PkJDPs Are Highly Diverse 

While the type III PkJDPs are highly diverse, almost all of the members were found to be homologous to at least one type III PfJDP (Figure S1B; Table 1). Interestingly, a number of the type III PkJDPs appear to be homologs of type III PfJDPs that have been partially characterized or are homologous to human JDPs, allowing speculation on their putative function. While proteomics localization studies have indicated that certain type III PkJDPs might occur in the PV, the PV membrane (PVM) or the HCC (PKNH_0717100; PKNH_0935000; PkNH1009500; PKNH_1317800; and PKNH_1419600), based on the localization of their homologs they were overall deemed parasite-resident [15]. PKNH_1419600 is most likely a homolog of PfSec63 (Figure S1B; Table 1; 82% sequence identity). PfSec63 is homologous to human Sec63, an ER membrane-bound protein with a lumen-facing J domain, shown to associate with and activate the human ER Hsp70, (glucose-regulated protein 78, GRP78; also called BiP), which interacts with the translocation machinery responsible for protein transport into the ER [64]. Similarly, PfSec63 may activate the ER-localized PfHSP70, PfHSP70-2 (homologous to GRP78/BiP) in the context of protein translocation into the ER [65,66,67,68]. Hence, it is tempting to speculate that the type III PkJDP, PKNH_1419600, is located in the ER membrane such that during protein translocation into the ER its J domain can recruit and activate the ER-lumenal PfHSP70, PKNH_0715900 (a homolog of PfHSP70-2) (Table S1). The type III PkJDP, PKNH_0319800, is homologous to the type III PfJDP, PF07_0103 (PF3D7_0724400) (Figure S1B; Table 1; 81% identity). PF07_0103 is homologous to the yeast inner membrane translocase subunit of the mitochondrial import machinery (Tim14 [69]), and hence it is also referred to as PfTIM14 [8,13]. PfTIM14 has been proposed to cooperate with the mitochondrial PfHSP70, PfHSP70-3 (PF11_0351/PF3D7_1134000) [13]. Hence, PKNH_0319800 may be localized to mitochondria and serve a similar function to PfTIM14 in collaboration with the potential mitochondrial PkHSP70, PKNH_0932200 (Table S1).

### 3.4. Type IV PkJDPs Have Unusual J Domains

The type IV PkJDPs, PKNH_0941100 (homologous to PF11_0443/PF3D7_1143200) and PKNH_1129500 (homologous to PFF1010c/PF3D7_0620700), were found to be parasite resident [15] and are homologous to type IV PfJDPs of unknown function (Figure S1B; Table 1). They both have unusual J domains missing key helix II residues important for functional interaction with HSP70, as well as corrupted HPD motifs (a “KNS” for PKNH_0941100; and a “SVH” for PKNH_1129500) (Table S1; Figures S3 and S4). This suggests that these type IV PkJDPs are unlikely to be capable of directly interacting with HSP70 in a typical fashion, or they engage HSP70 in a novel functional manner, a non-functional manner or an inhibitory manner. The type IV PkJDPs membership is also notable for the absence of homologs of key PfJDPs, especially exported members involved in survival and pathogenesis of the falciparum malaria parasite (e.g., PFA0110w/PF3D7_0102200/RESA [70,71]; PF10_0381/PF3D7_1039100 [72]; PF11_0034/PF3D7_1102200/eCiJp [34]) (Figure S1B).

## 4. Conclusions

**Figure 5:**
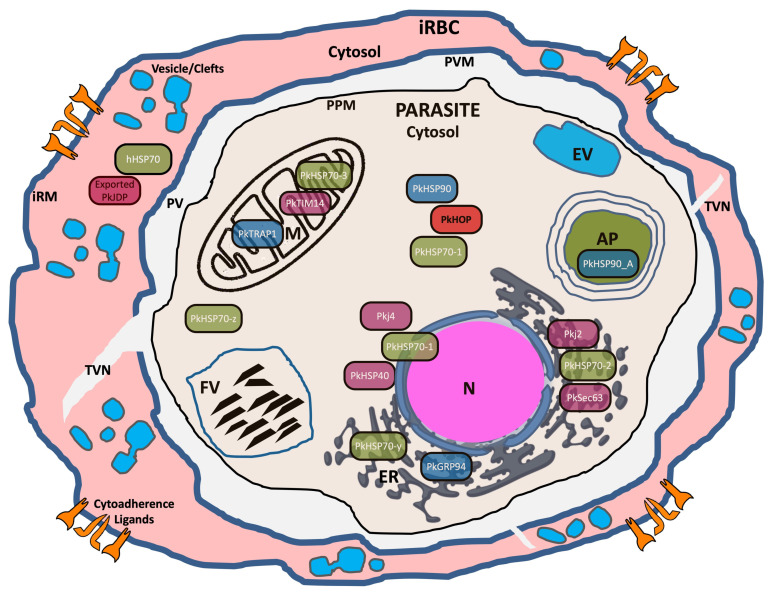
Schematic representation of a *P. knowlesi*-infected red blood cell showing the potential sub-cellular localization and chaperone partnerships of certain PkJDPs. The PkJDPs, PkHSP70s, PkHSP90s and PkHOP have been given common names based on their homology with chaperones and co-chaperones of *P. falciparum*. The PkJDPs include the following members: PkHSP40 (PKNH_0424600); Pkj2 (PKNH_0906300); Pkj4 (PKNH_1311500); PkSec63 (PKNH_1419600); PkTIM14 (PKNH_0319800); and Exported PkJDP (PKNH_0216100). The PkHSP70s include: PkHSP70-1 (PKNH_1312700); PkHSP70-2 (PKNH_0715900); PkHSP70-3 (PKNH_0932200); PkHSP70-y (PKNH_1257200); and PkHSP70-z (PKNH_0107400). The PkHSP90s include: PkHSP90 (PKNH_0107000); PkGRP94 (PKNH_1441400); PkTRAP1 (PKNH_0915900); and PkHSP90_A (PKNH_1238400). There is only one PkHOP protein (PKNH_0420900). Refer to the main text and Supplementary Materials (List S1 and Table S1) for the details of each protein. The infected red blood cell (iRBC) cytosol is shown in pink, and the parasite cytosol is shown in pale yellow. Unlike *P. falciparum*-iRBCs, *P. knowlesi*-iRBCs do not appear to have knobs or Maurer’s Clefts; however, there is evidence for cytoahherence ligands in the iRBC membrane (iRM) [73], and cleft-like structures, vesicles and a tubovesicular network (TVN) within the iRBC cytosol [74]. The abbreviations used for other sub-cellular features include PV, parasitophorous vacuole; PVM, parasitophorous vacuole membrane; PPM, parasite plasma membrane; AP, apicoplast; ER, endoplasmic reticulum; EV, endocytic vesicle; FV, food vacuole; M, mitochondrion; and N, nucleus.

The co-chaperone–chaperone machinery for the folding of newly synthesized or misfolded proteins within the *P. knowlesi* cytosol is potentially all in place, with the assistance of the canonical type I PkJDP, PkHSP40 (PKNH_0424600), and certain typical cytosolic type II PkJDPs (e.g., Pkj4/PKNH_1311500, PKNH_1246700 and PKNH_0407900), serving as co-chaperones of the putative major cytosolic chaperone, PkHSP70-1 (PKNH_1312700) (Figure 5). Perhaps not surprisingly, type III PkJDPs have the largest and most diverse membership, with only a few members whose function can be predicted with confidence. Indeed, there is a putative ER-membrane-bound PkSec63 (PKNH_1419600) that potentially recruits PkHSP70-2 (PkGRP78/PkBiP; PKNH_0715900) to the protein translocation machinery of the ER (Figure 5). In addition, a putative PkTIM14 (PKNH_0319800) was identified that would potentially recruit PkHSP70-3 (PKNH_0932200) to the mitochondrial inner matrix protein import machinery (Figure 5). The type II PkJDPs also have a putative ER-localized member, Pkj2 (PKNH_0906300), most likely a lumenal protein potentially serving as a co-chaperone of PkHSP70-2 in the folding of ER proteins (Figure 5). However, of particular interest was the finding that an exported type II PkJDP (PKNH_0216100) could potentially interact with human HSP70, suggesting that it may be involved in the trafficking and folding of other exported *P. knowlesi* proteins (Figure 5). Experimental studies on these predicted chaperone networks are needed, not only to validate their predicted function and role in pathology, but also because a number of the PkJDP-PkHSP70 partnerships are potential drug targets for the discovery of novel anti-malarials against knowlesi malaria.

## Figures and Tables

**Table 1:** Gene Identifier, Localization, Homologs and Type Classification of *P. knowlesi* JDPs.

Gene ID ^1^	Localization iRBCs ^2^	PfJDP Homologs ^3^	Type JDP ^4^
*PKNH_0307500*	Parasite/Parasite	PFD0462w/PF3D7_0409400/Pfj1	I
*PKNH_0424600*	Parasite/Parasite	PF14_0359/PF3D7_1437900/PfHsp40	I
*PKNH_0216100*	HCC and Vesicle/Vesicle and HCC	PFE0055c/PF3D7_0501100; PFB0595w/PF3D7_0213100/PfSis1; PFB0090c/PF3D7_0201800; PFA0660w/PF3D7_0113700	II
*PKNH_0407900*	Parasite/Parasite	PFB0595w/PF3D7_0213100/PfSis1	II
*PKNH_0906300*	Parasite/Parasite	PF11_0099/PF3D7_1108700/Pfj2	II
*PKNH_1114800*	ND	MAL13P1.277/PF3D7_1356700	II
*PKNH_1120300*	Parasite/Parasite	PFF1415c/PF3D7_0629200	II
*PKNH_1246700* (*alternative start* ^1^)	Vesicle/HCC and Vesicle	PFB0595w/PF3D7_0213100/PfSis1; PFE0055c/PF3D7_0501100; PFB0090c/PF3D7_0201800; PFA0660w/PF3D7_0113700	II
*PKNH_1311500*	Parasite/Parasite	PFL0565w/PF3D7_1211400/Pfj4	II
*PKNH_1344400*	Parasite/Parasite	PF14_0137/PF3D7_1413900	II
*PKNH_0112400*	Parasite/Parasite	PF08_0115/PF3D7_0806500; MAL8P1.204/PF3D7_0831200; PFB0920w/PF3D7_0220100; PF10_0378/PF3D7_1038800/Pfj3; PFL0055c/PF3D7_1201100	III
*PKNH_0319800*	Parasite/Parasite	PF07_0103/PF3D7_0724400/PfTIM14	III
*PKNH_0717100*	PVM/Parasite	PFI0935w/PF3D7_0919100	III
*PKNH_0718100*	ND	PFI0985c/PF3D7_0920100/PfJac1	III
*PKNH_0801600*	ND	PF10_0032/PF3D7_1002800	III
*PKNH_0804300*	ND	PF10_0057a/PF3D7_1005600/PfJjj1	III
*PKNH_0924200*	ND	PF11_0273/PF3D7_1126300	III
*PKNH_0935000*	Vesicle/HCC and PVM	PF11_0380/PF3D7_1136800	III
*PKNH_0939900*	Parasite/Parasite	PF11_0433/PF3D7_1142100	III
*PKNH_1009500*	PVM and Parasite/Parasite	PFE1170w/PF3D7_0523400	III
*PKNH_1031100*	ND	PFE0135w/PF3D7_0502800/PfJjj3	III
*PKNH_1207800*	Parasite/Parasite	PF14_0700/PF3D7_1473200	III
*PKNH_1270100*	ND	MAL13P1.162/PF3D7_1330300	III
*PKNH_1317800*	Parasite and PVM/Parasite	PF08_0032/PF3D7_0823800	III
*PKNH_1335700*	Parasite/Parasite	PF14_0213/PF3D7_1422300	III
*PKNH_1347000*	Parasite/Parasite	PF14_0111/PF3D7_1411300	III
*PKNH_1407500*	Parasite/Parasite	PF13_0036/PF3D7_1307200	III
*PKNH_1419600*	PVM/PV	PF13_0102/PF3D7_1318800/PfSec63	III
*PKNH_1436500*	Parasite/Parasite	PFL0815w/PF3D7_1216900/PfZuo1	III
*PKNH_0941100*	Parasite/Parasite	PF11_0443/PF3D7_1143200	IV
*PKNH_1129500*	Parasite/Parasite	PFF1010c/PF3D7_0620700	IV

^1^ Gene ID = Gene Identifier for each *P. knowlesi* JDP [14,36]; the protein sequences for all PkJDPs are listed in the Supplementary Materials (List S1); re-analysis of PKNH_1246700 revealed an alternative start codon and a more complete protein sequence which was used for this table. ^2^ Detection and localization in sub-cellular fractions of infected human red blood cells (iRBCs) subjected to proteomics analyses; localization was determined by two algorithms with up to two possible locations for each approach, first most probable and second most probable; the first prediction methodology, “used an ‘iterative Permutation’ (P) algorithm”, and the second prediction methodology “used a parallel ensemble ‘Machine Learning’ (ML) approach”; ND = Not Determined/Identified; HCC = host cell cytosol [15]. ^3^ Potential PfJDP homologs were identified on the basis of phylogenetic analyses; the Supplementary Materials contains the details of the analyses, including the sequences used (List S1) and the phylogenetic trees (Figure S1A,B). ^4^ The JDPs were categorized by type; see main text for the definitions of types I, II, III and IV [8].

## Data Availability

All the data associated with this article can be found in the body of the article and in the Supplementary Materials.

## Supplementary Materials

The following supporting information can be downloaded at: https://www.mdpi.com/article/10.3390/ijms252212302/s1.
