# Supplementary material for: The J Domain Proteins of Plasmodium knowlesi, a Zoonotic Malaria Parasite of Humans
Source: Int J Mol Sci. 2024 Nov 16;25(22):12302. doi: 10.3390/ijms252212302 (PMC11594657; doi:10.3390/ijms252212302)
Supplement: Supplementary file 1 [file ijms-25-12302-s001.zip › ijms-3281706-supplementary.pdf]

International Journal of *Molecular Sciences*

Review

The J Domain Proteins of *Plasmodium knowlesi*, a Zoonotic Malaria Parasite of Humans

Michael O. Daniyan [1,†], Harpreet Singh [2,†] and Gregory L. Blatch [3,4,5,*]

[1] Department of Pharmacology, Faculty of Pharmacy, Obafemi Awolowo University, Ile-Ife, Osun State 220005, Nigeria
[2] Department of Bioinformatics, Hans Raj Mahila Maha Vidyalaya, Jalandhar, Punjab 144008, India
[3] Biomedical Biotechnology Research Unit, Department of Biochemistry, Microbiology and Bioinformatics, Rhodes University, Makhanda 6140, South Africa
[4] Centre for Molecular Medicine and Innovative Therapeutics, Murdoch University, Perth, WA 6150, Australia
[5] The Vice Chancellery, The University of Notre Dame Australia, Fremantle, WA 6959, Australia
* Correspondence: greg.blatch@nd.edu.au
† These authors contributed equally to this work.

SUPPLEMENTARY MATERIALS

Citation: Daniyan, M.O.; Singh, H.; Blatch, G.L. The J Domain Proteins of *Plasmodium knowlesi*, a Zoonotic Malaria Parasite of Humans. *Int. J. Mol. Sci.* **2024**, *25*, 12302. https://doi.org/10.3390/ijms252212302

Academic Editor: Nikolas Nikolaidis

Received: 12 October 2024
Revised: 7 November 2024
Accepted: 8 November 2024
Published: 16 November 2024

Copyright: © 2024 by the authors. Submitted for possible open access publication under the terms and conditions of the Creative Commons Attribution (CC BY) license (https://creativecommons.org/licenses/by/4.0/).

List S1: Protein sequences used in alignments and/or structure predictions

P. knowlesi JDPs (J domains highlighted in green)

P. knowlesi JDPs: Type Is (2 members):

>PKNH_0307500
MMVAPLRILRNGWNGKLLLRGGSIIGSSGNEKGCGALPWNFTKRNINISRRWLNQDPYTVLGLSRNATTN
EIKKQFRLLAKKYHPDINPSPDAKQKMASITAAYELLSDPKKKEFYDKTGMTDDMNYDGHGSSGSASNFE
GAFSGFGDASFMFTDFAEMFSNMAGSKTTSTRGEDIQTEITLKFMEAIKGCEKNIRLNVKVSCNNCNGSG
KKPGTNLTICKVCNGSGIQRIERGPIIIGVPCRNCSGNGQIINNPCKQCSGSGVKFQTKNITLDIPPGIK
KGMQMRIPNQGHSGYRGGKNGHLFVTINIEPHKIFKWVDDNIHVEVPLTMKQCLLGGVIKVPTLNGDMDL
LIRPKTYPNSERVLKGKGPCKVDSHTNGDLIVKFSLKIPDKLTPRQVELIEEFNRIELNQGRDDTDEKFN
VGNSGSGGGNVGDNGAGVRTSREAGAAKGAENHVPEPPPISQKKKNNVNNWEGKNDSNVPIPPPPPKSTR
QGGEAHRGEAHREMKKDTRLHNNMNTTHSNGEGNAKHTSSFDQFNMSSNNMSTNKTGNSSYKMDDTMNKA
NSSGVGVSPDNTINGSNQSADSYQPAGTETRGGNSSSTFSYAKKWISDKLRPKT

>PKNH_0424600
MFFSSGFPFDSMGGQQTRRKREVNNSKYYEVLNLKKNCTTDEVKKAYRKLAIIHHPDKGGDPEKFKEISR
AYEVLSDEEKRKLYDEYGEEGLENGEQPTEATDLFDFILNAGKGKKKRGEDIVSEVKVTLEQLYNGATKK
LAISKDVICANCEGHGGPKDAKVDCKQCNGRGTKTYMRYHSSVLHQTEVTCNGCRGKGKIFNEKDKCANC
KGGCVLKTRKIIEVYIPKGAPNKHKIVFNGEADEKPNVITGNLVVILNEKPHQLFRREGVDLFISHKISL
YESLTGFVAEIVHLDERKILVDCTNSGFVRHGDIREIAEEGMPTYKDPFKKGNLYITFEVEYPMDLIITN
EKKEILKILKKQNEIEKKYDLENSECEVVTCQTVDKEYLKQRLSKQQQQDAYDDEDHQPEMEGQRVACAQ
Q

P. knowlesi JDPs: Type IIs (8 Members):

>PKNH_0216100
MAISMKHSTKKEINVFPFSVKLFLFSFLIWIITGSNQGNPSRKWGHSEHHSLKKKVDFRNNRWLAGRDSQ
GEASHERDNFENLEDYYAILGVPKDATENDIKKAYKKLTMKWHPDRHVDPEYKKIAEEKFKIVLEAYEVL
SDDYKRRIYDLYGIEVLKGNFTIYDDGEERGISDHPIFSFYKPNINASEMLNKFIDPVKNFSFKSAFNER
FQQVSDFINNVKSKINSPPTPGGTTWDNTPKSCEASLPVTLEELYNGCQKKLKVTRKRYNGPVSYDDQKV
LTVDIKPGLCDGTQIIFQGDGDQVSPWIEPGNLIFNVITKEHNIYTREGNNLIFRCVLTLDEALNGFRFG
LITLDNRELIIRVDDIVAPNSRRTIPNEGMPILNNPSKRGDLIIEFIIVFPPNLSPEEEDTLNDILCNRR

>PKNH_0407900
MGKDYYSILGVSKDCTTNDLKKAYRKLAMMWHPDKHKDVKSKKEAEEKFKNIAEAYDVLSDEEKRKIYDA
YGEEGLKGSIPTGGGTYVYSGVDPSELFSRIFGSDGHFSFSTGFDDDFSPFSTFVNMTSRKSRPSTSTNV
NNNNYNSKPATFEVPLSLTLEELYSGCKKKLKITRKRFMGSKSYEEDNFVTIDVKAGWKDGTKITFYGEG
DQLSPMSQPGDLVFKVKTKTHDRFVREANNLIYKCPVPLDKALTGFQFIVKTLDNREINVRVDEIVTPQT
KKIVSKEGMPSSKIPNTKGDLIVEFDIIFPKNLTSEKKKIIREALVNTF

>PKNH_0906300
MKVVNVRKRKKITNMHSVILLVLSFFLSFGRGMDYYKRLGIKRNASKEDISKAYRKLAKEYHPDVAPDKE
KDFIEIANAYETLSDPEKRKMYDMYGENYADGASPGGAGGGGPGGFGNGFHFDQDVVNEIFRQFAGGGGR
GGGGGRAGNFHFKFTSSGGGPGGAGGGFPNFGHHFEDEYEDIYKNEVLKINSQNYDKVINDITYSLVINF
YSPSCSHCKSFKKKFLKFSKKYDGYLTFSVVNCQEEKSICRKYNVKSLPHIILLKKNKTYETFYGNRTEE
NLISFIEENIPYVYTDITSQRKLDSFLVKNVDTPKVIFFISYNDDVVMLKALSIEFEKRIDIGVIHSSNN
KMMQLFSRRNVRTPSILLVEDIDSLSGDLTFLKNFDFNILSLKLSHVVAQNRLKNNLYGHVTSYQELTKK
KFESGQCQEKDSQICFLILKLLKKSYHKFDEDIKNVAGKFSKDPLKIMYVNVFEQPHILDSFGLVNECKH
ADCLFLVAFRPKRQKFRLHDGEVNVQSVNKFVEDVVSGGISINQAVKRGLRFVGASHYTDEL

>PKNH_1114800
MNYHKILGVTKNACKKTIREAYLKKVKLYHPDLNKSPDATSKFKQIQEAYQALYNNDYAQKSHYGESSYG
KGNKAEDSKNNQSYSYSDDFTDFHKAFYEEVRKMRENERREQNRRYANSNYSYSLNDLFHKYPREHFYIN
LIFKLFPLFVVPFLFLFIVYKQYLLKSHFSDKPILIYDAYGRAFLIDSHGRKFRAAEFDKY

>PKNH_1120300
MIRRKLYCSFLFLAILETFKVNQRWSIFISAWYAHEDVDSDYSHSKLYEVLGVHKYATTEEIKKAYRKLS
KKYHPDKAKDKNSNNRFNEIAEAYEILGDEEKRKVYDHHGLEAAKNVESNKMDEDPTDHFNIYESFFGGA
GGFRREEMKKADSLTLNVEMSLEQLYNGDFFSVIYTRDVNCLRSDDCIMKKKECSGKGYKTVTQQVAPGF
IMQNKMRDENCIDRGKAWNPKCSYCPNGMKEEKTIELTLEVEKGMKNNDKIVFEKKGKQEIGYESGDVIF
VIQTKKHKVYERKNNDLHQFYEISLKDALIGFSKDIDHISGAPIRINKQTVTFHNEILKVQNKGMPIRDS
SKYGDLYIKFLVQFPKYLTEEQKRAISQFL

>PKNH_1246700 (Original)
MKWHPDKHLDENDKKAAEEKFKIISEAYDVLSDPDKKRTYDLYGEEGVKEHMSGDDMNFFNAGMDPADLF
NKFFGSSKNFSFTSVFDDDFPSFSSFVHNMGNMHGQPSGTSAGKRSDSYKSESYEVSLLLSLEELYNGCK
KKLKITRKRFNGIQSYDDDKLVTIDVQAGWNDGTTITFYGEGDQSSPLLEPGDLIFKVETKEHDRFEREG
NNLVYKCHVPLDKALTGFQFTVKSLDNREINIRVDDIVTPNSRRMIPKEGMPYSKNPSKRGDLIIEFEVI
FPKSLTSERKKVLREVLANTF

>PKNH_1246700 (Alternative Upstream Start)
MYYSVLGVPKDATENDIKKAYKKLAMKWHPDKHLDENDKKAAEEKFKIISEAYDVLSDPDKKRTYDLYGE
EGVKEHMSGDDMNFFNAGMDPADLFNKFFGSSKNFSFTSVFDDDFPSFSSFVHNMGNMHGQPSGTSAGKR
SDSYKSESYEVSLLLSLEELYNGCKKKLKITRKRFNGIQSYDDDKLVTIDVQAGWNDGTTITFYGEGDQS
SPLLEPGDLIFKVETKEHDRFEREGNNLVYKCHVPLDKALTGFQFTVKSLDNREINIRVDDIVTPNSRRM
IPKEGMPYSKNPSKRGDLIIEFEVIFPKSLTSERKKVLREVLANTF

>PKNH_1311500
MSKRVNYYEVLGVPQDADLSIIKKSYRTLAMKWHPDKNPNNKAEATEKFKQISEAYEVLSDPKRRRKYDL
YGTDENYMPDENDEFSNFHKNFGFNDAQRIFEMFFGDSTPFGNESFFSEVMGSSFGDSRRGRMARSNDPF
DNFFGSSFNISFGSSNFDTFMDGGSSFTSVETSTSNGGKFKNRFVKTSTSKTTSIINGKRVTRIETVKTL
PNGTIERTVTEREEDGRGNVNVRQLPAHEMRRNKR

>PKNH_1344400
MEEIIIHMIIIAPLTKWLIGPNRSWNKGKREYGVYIALLLLFCVGIYELRKTNQNLYEVLQLNAYASKTD
IQQSFRRLSRVYHPDKNKEADSFERFNKIREAYEILSNEKKKYIYDRFGDFAGSEITNFFYVEIIIIAIF
QFAISFIFGFLYTYGKDNEKYRILICLYIALNFCIELVFRFSPESTHFLSFLPIFCHYTPFERIHSLRVL
VPLVMNAILLLDVYFIDEDTDLYVSTFCEYVFENSQKTIKNMDDVVIFCARLVDGKVNKANNFSWREKDP
YSDLANIEELEDEETYDKKCDKSDMFYKLLYNVVETSTEDVELKIPKKSLCRRFDWSRLYTESVMEKNTE
EKDFEQSNASKGILFSSLLYFIGLISHLVSK

***P. knowlesi* JDPs: Type IIIs (19 Members):**

>PKNH_0112400
MSNWGGFFGDYGNWFTSPGNHIATGNVGTHNVDTNMSTNLAAAAATANNSSASSFFEDNLDPRIRDGGKN
STSIGGNPHDKSRHKYKFKYSRKDRSDERSRSGRMDNGYRDHSSSSGSREPGSDRESRVKREKLKEKKGD
RKEEMGSANNPIGVGSCAHPCGSAQEAFSSGGSVPSGASPIGATPIGASRSSSGESIGGFFSTLGLGNLF
SNLENRTFCNEYKTGYCLDEEAKKVLQKDGDSSSKNSSSKKSSKIGIGLDGKGVFNSESPEGNPICVDTT
YYDILNVKPTATFSEIKSSYYKLALKWHPDKKGDDPEAKVKFQKINEAYQVLSDSERRADYNKYGLNATK
DMVVIDPSLLFMMLYSSDELSDYVGTLRVAFFIKLAFECNSTIEDIQTQGGKMFSEMEVEQSKREIELAL
LLRKRLQPYVDGDTKWVERMEKEISDLLDSSFSSSILESIGWNYRNSASSFIAEVTTLWGMGATLPNIQA
QKRSVQNNFGLATSLISTFFTMQKMVAYNEMNDSLEGADLGEEAKVISGGKVNCEENKLQKEKIQDNGSG
NVRDNASAGYSSEGYSGADHLGGKTAGGNFTHKVADKFSDFFGEKRKEKKKQKKEDMEGAEGITQPSRSS
TELVDDGIPQSQQTHDDDTSKRKTCQKKDMDREAYLEKKNNEAFAIIIKNVLKVVLWDIESTVRKVAEKV
LRDEGVSIETRLQRAKALKLLGKIMLRLSKTKKDMSDSKEFDVNQLFESVILKVAQKAAAEEEAASRRED
EDFFKRESY

>PKNH_0319800
MWPVVILLFGGGILFAKKGMNYLKNQKINPGGTSFFPSSGFNRSLGNLFLKNDMRGFERNMSKSEAYKIL
NINPTTNRDRIREVHKQLMLKNHPDNGGSTYIAAKVNEAKDVLLK

>PKNH_0717100
MLNDIIFQVIIASFGVTIVNSDKIKFLQKFRYAIYVLILSFLLYKGVPWKRENYYTYLNITPNATKQEIQ
TAYRQAAKIYHPDKNPDESADSAFIKLKQAYDVLTDDTRRSNYNRFGDYKNGEVDDNTATLLICLSLVQH
TMFFIIGYFLSYPRKLVFARQIFLVYNIASFCFELQFRFIEDDTTFDWLPSIGYLLPYEKIKLLRMLFPI
VFFISICAAAYTYTDRNATLIYLMRSILATNRIVFERSNDVVDSTNYLKKNGEQLVSKLQEMRKAVEDAK
YAQAKLKDKEQKDKSECLEDSKKDPKDENNADDKLRENVQEFALTLDSHQMGLLEKCFEMMKNKNTKDKK
NQKKSWFEFISMQMIFGIIFVYIWFTSK

>PKNH_0718100
MNKVKYVPLLWRHKAVLNTTGRRRYHHNINNLEKIFYSSGKYTNTARRITCHKCHCDISLDVVPLSCETC
SALLHVDAFKQFNFFELFGLQATYDIDKGHLKQKFNNIQKLYHPDKHAQNEQLEQINEVSSYLNSAYKTL
QNDVDRALYLLSIQYNYKIPEEENLEDSEFLAEIVQVNERIGDPEANITLMTKEYKDKYEEHIEKIKLYF
DKGDFEHILMALKKLKFINRILDRLQNV

>PKNH_0801600
MKNNKATENYYMKEPPCDHGNNVINKCSSYFDISQIKQLYAPQSTNGYEQIFSIPRKAKKTGRKSKKEIS
NTKGSISSFHSDQYENDKQKNPVPIRIGLRKKMKYNSPNEDSHFFYSNSFDQTPRKHFSFNHKDPQNDEP
PNVYNPRGSVTKGVNKRPTSRSDSLNKYDFIRFVCSPTKKSVDNRSANCDSEMCSAMKSNTITNDNTRND

NTRNDSTRNDNTRNDNTRNDNTRNDNTRNENTRNDNTRNENTRNDNTRNENTRNDNTRNDAFKNHPMNIA
NCGEHTATSVASMNDPQRVVNNYVQWDQFFHPKTEPEPPNALKRRTKYDPNIIQNNDIMNDTKGAISSAR
KIYPPNESILNPKGGTKCVERVGRSGTTNSHTGEDTKSAIRNFTKHYPPHELYNDTDESREGEQERTPQN
GAQNNGPIDLDSHDSIDRLSRNFFINNKKIFLTHFRKDKETQTKRGSNYSHSTGGNVPLRHDATQGERNK
GKNEQNKWWKNGEEVTPGGESFHCGHFAKVNSPHCEEYIHSEEEMCRAENSSRRNYHQGEDQAKGDDWNE
DDKHLNHDTRYSDEEPIDGRNFLPDMNSFVRKNCNVMKSLYSHLGVPYNGSKEEIKKSYKEKIKVHHPDK
GGSVNKFLQLKLSYDILTNDKKRKMYDKYGHGILELLVSENFQNYEISSSDGENNQNEIIDEENIKIYNF
FVQKYNNTSYLHNLQDLKMDSYQYNEFQKLIFHFFHHENPMFKNTFHLFPAYSPFVPPYRKKKTDKRKTD
KMKADKMVEVEKGANGKEVDKKMIPHLDLSNSLDNTPVSSIPTKELFSSSESCTSRNYSPSNYSAKKECS
INDVLSEEHVTNLFNEMENDFKYFYNKFIVHKIEESELAKNNSTYGKKAATKIVNPADHNNVEVKGNSDD
VSTPMKEHTHHSFEQFPYDEDMATPLAQCQLSHEKHSSNRWRQREKDYSFRDIQTSPIAYKIINENNERH
IADFYRWFELFFNHRQGDQAAKEAVNQAVNEPVNVAANEAAKEVPNHATQNHHRICSEMAANWHSGATTP
IVIPPPKLQEEAHSAQTNEAHAKINSESPTKFQCSAENFFSFGRMPHPSSNSPNVRIKDNTQNEGKPTIF
SHYKYAKIQHGGGGGTTPLQTQSNATPTRTNTTPRRSGKKNDKPNISREKSAHKITPLCNSSSKMENSVT
INIEEKYGFNLLKRSEQKIKDITHDFNYIYIGSNMMKKNKFVLFVKEESVKKFLKVKLLIEKIKKKSNLK
LISVFEKEIEKNIKHMEYILLLTTHKDEFLPLNDFYLLDRNAFTKDHYCIPLYIKKNTISRPTHFHIRWI
VYTLQSLIFFNFFFKKMNKYMCAFPFFNYKYNLIKHFLDHEEKNNCSIHQHIKKFYIFFQQYIIKNKQHY
LKYFPHSITLHIKNDASPNYQNSKPVCINMSSILVLTPNKPIPSSEW

>PKNH_0804300
MDIEKCKCFYEILNVESTATVEEIKKSYKKIILQYHPDKNSHLSEEEQRRCTNIFRQVQEAYECLVDERR
RKWYDKNRLRIIAGKENEEKRDQNRQARRRSGSAGSSESGGGPTSGINIWEYFSSSCYEGFNDKDERSFY
NVYRKLFEEIIEEENEELNMRRRYGKKRHEEDEENKRNNVHAPSFGNSKSNGKEIDEFYSYWSNFSTVKK
FDYSYEYMKMYEQENRHVRRNLKKAAEKRSLKERKEFNENVRSLVEHLKKHDTRYLNRVLELAEEKRKKA
EEKERQRREQMLQRKYLFEEIKEKREDPEGQAQGDKLPSEEEQPCDYSSCSEQGDRMKGNREGEEQRSGG
KGQGNYNSHCEDAGGEDGESGGEDKNSENPYSEVIYRCEVCRKNFKSMKQYNSHEKSKKHVSNFLKHAKR
YAAGGIFDAEPQGKWLDGQVDEQMDEQMDEQMDEQMDEQMDEQMDEQMDEQMDEQMDEQMDEQMDEQMDE
PSFQQSDNSDGQREKRSAKSKTKKKKNKKSRAQMNNFSESVNSASESSHQDEDVLSWYKGKKQHRNKFPY
TEEEEGISDAAESLQREESPQEDDSAKQVKASPAQAENTMNSSDDYTSSKRKKKLLKKKKKKEGTNRKEN
DEKKLDEPKEDNKDISGKGNSKSKIKESTKNLVCKMCKETFDSRNKLFAHIQKEGHAANKVVVDVSAKVN
KNKKGSSKREKLP

>PKNH_0924200
MGKMKGSINAEVSEGDPDDVVRKEKMGHERDDHQSPEKSVTSNGMSNLTSRGEQSQKEHAPPIWAGMNHH
MRITKQGSPNKKSTSAKKNFPLKSNILAPMRKHTILRSTDTIRECISAKGTMSKHSVGETRPTSEIEPTI
KSPISHTFQRNERKKIKMKNITSIQIPAHRNVSNSVKRNPQQILKNYIYMKNSERNNFYMNTKCTDKKGQ
TQRFPLHLLKERILNFTKRPRDIISKLSKNCNSSNHLEKNKKKIKVDKAEENDDVILLDGDQNLPCRSGE
NGPKSSNTENNNLLEEYNKGKHTVGSSTFTFLKIPLAVKRKHGKITIKGNSIAIARAGIKNKQSSANSTS
RQKNDSVKYAPFKKIFNEEEAHKIELNYGNNFSGMEKKEEEKVKNFYSKFYKCTQAAHEESETNPDCSRD
ASDLHTFCLRTLDTPSKYCKSCKKFLKLLISHGTKYRGTLIGINFICSDVQNLLFAYKCDKQHEFNLSLF
HIMHNLWCPHDYCLFECKGKHEKNYATEFFRLKELDTMEKQKDLFLKAKLFFRVYPENATPVNSENASGE
CTDDVERIIRNANDPWKVLQIKKISKMELCDKELKREARKNYRVLALKIHPDKNKSKNATLAMNILTNSM
KAITSA

>PKNH_0935000
MIGNKEDAYECFNLANRYLKAGNYSHAKNLFLKSKRMFPDIDISEQVKICEEELRKRENAEAQSNSSHAN
GNNNNSDNYNSTTYRPNHNNLHERHVGAKDDGIGRILRTNNYYEILGIPKNSNDETIRAAYKKLAKLYHP
DKNKEKGTEEAFKKVSIAFQNLINKEKRHEYDNNSETDHSHHHPTYRTTHYYYNDDMFTPEDIFRNFFGI
NFATCNNRTFRTNMNNTRAYTNSSNSANSQRTHSFIQIAIFFIMFVIFFLSSHFEQPKAIYSLNKTSYFD
TLNYTSLNKVRFYTKKTFNYNYPKNSHPRFQIEYEVEYKYYEHECQMLTKKIKNDYYREKNQYQKKINYH
EIPESCMKLKTLEDQYNNYIMKLKRR

>PKNH_0939900
MNSKKNHLNKVGRQTGNPNLGRKNNSYKGSTYGQTNGGKVFKFMSDLKYNGYGKKLDGTHSDKSSSTSRN
GKGYGVSSKGKGKKVEHEMGEEEKDDEEDDEEDGEDSDEYEDDMEDEEEDEESEESEEDEEDNDDDDDDD
DGDDDEEEDETNSSETSEFSVKKKRKEKKLHKNDNFYEGVETSSTKNKMNPKNYNSDYYDIYSNIYSYYD
IFTEDKNEKKKYKYFKDVFSNFDHKYENKEYNYGVDLSKKLQEKHIPIIHSNRGGNHFDKPYTKEDRGNA
SPPISNIPAAEHINSNSAGGSAKLGSNINIAFLSNKIFRNGDGKNSRGRKENADNRDGNDNNNSTDVAAT
MSDLHNEFYAGSGSLKTKRSNLKKSEAHKDSVLKSNKKKFVPLKKKKKKKSTSEDISMNDRENLSSQFST
NHSTNPISAVSATGNASTNANPNVTEFKKNLGHPKNGAEDIYGKYDFSDLRRSANNEKVKKKFSHPVDDV
ASIAKKSKGRSNKKPDLFDNQENSEASSATIYAKYANPMKGRGKSHMDAESGTTHRKGRIDNVNNVSNAS
DTSNLSNPSNISLDSSCSIELDEPLEEGKVADVKKEGNLKFLKNPTKKRMNKLKKILEILEYSIYNEEKK
GSKLESKLYDYVLLTKSLKQEIANLEELNNSNKSKMANYEKDITKLKEQNEEMKLTLSTFENELSSLINK
FDKNFAKELIDTKTQNDVLKKEVEKLKMEIEKKNNEIKKMENWNDMSKKLNGDNIVMQGGEGVVPCSVNT
SGMNSTGTTNGPLNNGKMANGVVSSPNVETTGGNRIAALGDKSEQGGVPGGNPLEKNLKEPITTVYEFPF
YYGTSEDIEPSIVDDLILKMKTIRIVYEKNIERYDDDTKATIYGCYISNFILENKDICNHRKVLKHLQEH
CLVESKMLNVHLSLRRERCDNVPIKELEKEVIRQLENPDSDKNKEKVALINSIMLTLLFKNMHLYNLYRL

FVTCLKCDNFRWIRLMKKSLFLKFFDLCLIDSNVKGNMHLPNVKEAHLIQVPPEDALPDPQGNVPMENPH
QVQYNIKHPMFYCSNETLKRLISSMFLNFAKDPINPQNCDNIYHYVLQKKNFDLLKLLKLSGKNYNYIFN
KNGENKTPLDYLDNEDIRIDLISGYILDIAGKGADNYKNAKYEVAFELYSEALEKQIKLSESVRMGKSMN
ENIGKLYYNRARTLMHLNKWIDVIENCNNCLKYIPKYINAFDTQIHAYENLLEYENALTTYKNMCMKCNI
KPDDKEEKLKSQINATSFQILNVNRNCSVAEIKQAFSNLSKKWHPDKLGINSSPDIKKRHNNHFKRLFKA
KQLLLNDAERMKEKKKKETNYVYPQIIEDVNNHSQGNNRTSSHNRQKKAATTGDSVNAPSQQKDGNVTNN
SRNTNHNASTPHKDGVGGNAEHPPVNTNAGTFNNSTKDTSTFSGEAKNNFYHTYKDDIDKFQEKLKNFNK
GLNSESENNKNGNPFLNLFDGKLTNLNNEKVDENFYKKLKEEYEGLSDDEITNFKTKISNSINNLIQTEL
NLKREYQELCIKEKTNVIMNARLCILQEIQKALCVRLDKERKLKVLDSIIKGRNGEQSANQAKANEGVDA
TAEEIPREYERDVPRIGRSEGYAPDTQGEEEKDYLQKNSSKFANGGSQADEKWENSRRKSNADDNDDRFF
EDAAFFRDAQNTKGDSGLASADRKKGNAEGSDEHGLEGDHFTDGSNDDSYGGGSGVQRANKRNGDQMEHP
KNTQKEAAENKMHEDAAKEFFHDDDRGYFEDNEKNKNTPSGSSNGCGGGDNVDHRNRRKKKSAEFNDNNV
QWEEGETQTGESKKKNEKNKMAAENNDNENFIFWDEKGNAHHEGKETSYNFEAADIFQGKNEGKTQVKND
SNNFFESVSANQSSQINQQFRHSNGLPGECSSMGEYEKSFTGDRLEEKNFFFSSKGNKGKNTLNNTVGSF
YDGNAEQPNGNENDHFPQRVKEQMTKEEVDGGEDDVESNGAMTSSHAFAKSMCERGPFDFPHGGNYDYEL
ESRAREGGVAGESRHAREVHTEGGDAPNVGSGISANANSHKVNFPNEEDAYNYHRERMKDIMGTANGTPG
NVKKVSKNGSNSSYDNEQSDGEEDIQQGLGRMTKNGMPNNSVPNNLVPGSSFQYNNNFHVDSSIDKNSDD
GILRNDTYGTTNQHHVHGVGIKHLPPKVADHKEQDNNINGEENDPIKYSNTLKKEEQFADMRFPGSNSYV
RKGVDQNMFNLLNMDANLAGSNSSGNLNSIYAEKFMKKNLMYSNDAVDRERRRSSFKQQVGTKYASDGEE
VAKGEKFYGLYNDNIVGHSSGGTNASGDHQVNNFDPPFFSQPAHLSHDPPSNGRKNGHRNSTTRRDSNND
EAFANLNSYKYDQEIETQHQTPIRMSQQGYKNAEGGIFHNNSNSDGHYTNERYEENYQQERGYNRYYAPD
EEEDTPNGPNNDDDKNFFQTDINTNHSNTNNLYENDMNNPFPEEIDSKENSFPFDDNNFPNSSSNFYENH
LSKSYNYENVKLRNSLMYMNENEEKNKNLKKIFSNDLTSISMDKEFNQNSKFSFNQKVEKNKVKNGKGKV
PSEENNYVKLKMSSLDKVSPNVDASKARPFVLSSGLTMGATSTPAALPVEGSLPKKIFKKNVNADDKHKA
VDQMKRGKGKATDSAVTIGSRQNK

>PKNH_1009500
MSRSQSLENNQIIPKYDYESDREEKSLISNIFSTRKPKHAGAGLVSGLKSVTKGIIVGTSFLFISPYLCA
KAEGINGFFKGMFFGLLSAIVIPIISLGVASYQIGRGIMNTPESIAQKALGKIWDDEKREWYDFYYNLDE
EANKVLNEINDNGVNGGNSNSNGNATYEKKNDVNDDEYYNKNGNIKVKNDEFYRILQVPTNASQNEIKRQ
YYKLAKEYHPDKCSDSKAKEQFQKIGEAYQVLGDIERRRRYDKEGKNAINNMQFIDSTFFFTLLFGSEKL
DPYIGKLRMVMYVEYEQIYKDEDVQRIIVKEQNKREVQLALHLREILNKYIQGNKEEYIAKFEAEMKDLC
QTSFGHVILENVAWSYENCANQFLGDKYSLFGISGKYYKMQQKKRVIGTGLKFVKTLIKTSSLASQIKKK
EEDEDMSLEKTAKVNKKIEDSLPAIVETMLNICLIDIDQTIKGVCKKVFTDMSVDENMRKTRAESLIVLA
KVMKKIIQDFKKNNEITDTKKLFEDACMRAYQKQDDDYK

>PKNH_1031100
MENAPNSNPYSILKVEPNCNIETAKRQFKKLAIIYHPDKLNYHRNKERKGVEANEKNRTDETSALGIVPI
REQNQGDKEEDKNNAKKDNVDFVTLVWAYEQILKDIQHGSKSEKYENALNIKKVDLQYISEENAYIYFCR
CGDFFLFAENLYCEYYVIYACQSCSCSVYLTP

>PKNH_1207800
MKSDTKLDLYEILGVKKNASVKEIAKAYRILVLTYHPDKFAARRGKVVGEKSKTGSEEKENGKLDVLDKE
TVDEEVNDGEEETLTLEKCKEMFLQIQKAYEILRDPEKRKNYDEYGLEDDEYIEFKNYLNPKLFHERIKV
EDILNYEKKYKNSSDEKEDLLEFYNKFNGNLTHILEYIPFSEEADLTRYIDIYNSLFKSKEIKKTPDYDK
TLKNINNIIKKYSNLKKKDSKMNKKRKNSTPPLDDLVLAIRNNEAKRTIKINNLLSNIEKEYQKKNPKKR
KIKPPTEEELNEISKRLEENKKKNAASKKLKSA

>PKNH_1270100
MFFIRKRYFGFSHGLGISGVIHRRLLKEVPNCAFQRRPFSTKNFYEILNVPRNSSKNEIKQAYRKLALKY
HPDRNPNNRKESEKMFREITEAYETLSDENKKRMYDSQLNNGFSSGHFENNYTNTNSNTGTNYAYQTRRM
TDEEIEKVFKNVFGTMNLNDIFKSNLFGEGNFSPRGMENDFFTKFGVPGSYRSNSDNIKQTNIKTEIIPR
GKKIIEKTTKIITYHNGVVKQEITEREISGNSKEYDDMSDFDIFYKSNFNKVDPRARRSEFIDYANSEKG
KKMVKQVVRYGYGIISIAMRRILVNIVIQVIRRIVQGLIHMLRRR

>PKNH_1317800
MKSMSSYDTGPESSELSSFSLKRKSSSKGKGDKHSKGKNVEKSNSADYSDEKETKKKDEQKKKKNEQKDK
KSIMSNDQSKNAKSGKQSNVEKTPVDMFKSKLKLLTDNIIIIAIILSFMTLITFKYLEEKYSMEKYLEEG
DSFDYYEVLKCKRSDSINKIKKNYRDLSKIYHPDSNKDCKDCDKKFRDITKAYKTLSDPRLKKAYDHSKG
KVLKLIESNSINLTMRNYKDLVENSNDYWIIQVYSDTDNLSLNFSKIWEEAFDKYNEYISFGRINILTDK
KLVKAKVPFNVKIFPTIFILAPDGTYQLYSNIFNATSKDFQSYIISNYPNLIYNVHDFNKAYYSLMKKSD
SVSKSGSSHIDKNNKVLLLSNKNKLSLQAKHITFKYRNIYTTYAIRYNEIDSLTDEPLKKEIIDALKVLS
VKKSEYIKENENIDYFILVNNKAQVKIIRRISSTNIKNVYKDALMKNMVEINATNVDSVCSTVGSRHTYC
YVTIIDNLNNEDTLTTLKKIYQHVNNSYASFTSKLGADEIESNMFIQPVYLLKSSMTKNFSKFVKDKSVT
TYDSFFMDYSANTFATVNEIKNLSNYSQKKEDLSFLSNIYKDIEILTFEKIPKYCLPLGVNCLYNPKKTF
SYRLYNIFKRTSQMQVIVSAVVGFFLYPSFKQYGQLRYAYLAASIGATLLVTNIKDFLLLLAN

>PKNH_1335700
MDDDGSFIPKELVGKDIYQILGLTFEDAKKDNIKNIIRKRYLKSALLLHPDKLEASSKGTGPHGTGPQAT
GTSASNTERFNTLKSAYEFLMNEQLRNKYNLYIAHQKTKKKKNNPSSSNISLSRFLDKRKLDAQQQEKWM
FKRKLEAREREMANRGEQKNKWKNNPEEWGSNEKSDGPTESSYQNGKNSRKKKNSQQNEADREQNNLEKI
KKQNEDFVRRHSSHPSSQKKRQKNVCQGEDIGVDVDGDGDGDGRDRVIEVYLDNYPHNVDLLQRYIEQKE
LLTVFLDFNIQKYYLNRNEEQTQCERRVGMFSFSNRTEAIRAYLHFKKNGKHIDRHFKLRLAVPCNEGVE
MRSQGESPQKGGENEKPTKDNVDRMMNEMVDELDKMFSL

>PKNH_1347000
MDLFSIASNLQQIAIGGVNFVNNKLRKKSVSTECNYITYIVIGENEKQPYDTNIFFLPFKICGKIKLKEF
KEFFPFKDNVIFRFKIPVVDLIDVINEGIVNHAPLMKEDTNEQNKEKNIISDENEIKNILRQDQFSHLWV
DITNEEAYIPFYNSKIVVKVLFINNEEYREYTDIYFKNFQCCESNRPIYTLNDTNPCSYIVLGEEENDKR
NSTDNEEEEEEEEEKDHYFDEHMNETSPSNHLKNRHPSNHTDNIPSSHSNNKHTHHLEDEAENFVHNEYV
RNDPQSEATYKKGYPSEMPQHKQHQNNANRKREDDNFFQSIKKEASKNKASEYILQKNSYTSTTNLLLSG
RSNVQGGYEDMPMGRENNGANSGPYNSPDVDKKERQDVIKANVSNRLQELKDFRTQEEAKFKEKVILSEH
IKKQIVKWSKNLDGSYKDVKVMLSTLNEVLWDDSEWRQVPMSELISNSTMVKKAYKSAILLCHPDKHRGK
PVERVLRAEMIFQALNNAYKEKKNM

>PKNH_1407500
MNEPSSGETNLQGGEDDVSAKGRSYGGAGGEKDAESNMNPSDNNTSSAVEDNHENSAQKEGADELGEDAL
NEMFDDFLKDIENISSNMENQQEGKKLNKGDAESEIARLLANKNSSPFEIFGIHEDVDMEKIKSKYRRLS
VLIHPDKCKIEKASEAFHILNKAYEELKKDDIKEQYKSVYETAKKNIIKKLNLKKIRNELNEYLNKNEEE
YEITKEVQLLINEECENLLKVQKEKMEYAQKCKLANLQYAKEKEEEKMKEELKKEEERKLWIEGREERVN
NWKNYKKDNLKTEKEFHIYKNVGKKKEERTEEEKEKLKRISTTNPMEKDGNKKRRKT

>PKNH_1419600
MSQFRDTFTKDSKKEPLLSYDDSAFIFFAGTVLICVLIPCTYIYIKSLFNKIFNNVSNKLKKSKHNSVYR
SCACALCKEKLEKRNKSTTLWEKLGYRKIIQFFLLVIFWGLLFILVQQMLSTKPMQTFDPFEILEVHAGA
TVGEIKKAYRLKSLKYHPDKNPNDTSAAAKFILITKAYQALTDEISKENYEKYGNPDGPGMMKVGIGLPK
LLIDEKYQLLILSIFFLIFLVFIPATFIIYYQRQKQYGPNGVKVETLQYLTYTINENSRAKSYPEMLAAT
AESRDIEFRQGDDEYVKKMIDELVEPKKRTFRIPVITKNYFIILAHMQRRYDLLSEEMKEDLKQILQFSL
LITHSMIEISILRDWFLTAQAALTFRRCLIQALDIRSSSLLQIPHFTEGIIKHVHKGKLAVKEILDFVHQ
NHESRKGLNEMTDEEILDVKSFCNIVPDIKMTARILVEDESHIVKGDVASIYIHIDRTNLHENEAAGYIH
APFFPLPKFEEWWIVATYKGDDRILKYVHVKNCEKIIEEKLQFMVDRVGNLCVSIYIMSDCYFGCDKKLD
LPFRAYSQTEIKREIFVHPEDIELDNEPTLFQQMIGDVGRRDDSSDEEETGAGGDPNRRNNFAHGNTATA
APRNSQNNQTNNAGNGNSSGNNNRNNNGGGVGGGGNPPSSQKSVSRDHENYVPDDDTNEDSGDE

>PKNH_1436500
MKDIILPKNACTQSEEQFCLQDIKAKNEEDYASQLSSLPYISTHHGVSIRKKKVEPAGFMFYVKYEIALL
KGKKKKTKEGKQIPIEEIFLYKEKIDYLNELKGKNQGEEEPHSEGDHGGDYNKEKSTKNNKDKNYEDDYD
DTNNMDSSNKKKQEKANNKKSSNLVKKCINNNINVYEVLSVEEADDLETIKASYKKLILLFHPDKNKGTT
FLNQKEKERKRKKEREKGKDGNRNDDNAKAKGQHGSQETQKDFMYYMEKYNIEKLTPEEKKIMFLKIQDS
YAVLSDKTLRKQYDSSIPFDEYIPTLTELEEAPNFYEFLRPVFKRNAKWSAKKPVPDIGDEHTDIKNVKY
FYDFWYNFINWRDFSYQNEYNYEEAECREERRWMERENKKIQKKASKAENLRIIKLVDLAYNNDPRIIAE
NKRVKLEKQRKKELSILEKQKKIDGATNEMEKESTTNQQSNDKKNKDNKAAVKIWRHHIKSLCVTKLAKY
VDSDLVQERLSLMPFETLCEFIDDIYVFLNFNLQKGNSLSVEKTNVSNDNQVIKQSQGGKDVTHIAHGKS
DSSALINTNSVYENGSSSLVGMNGIAKQDSNNIVKKDPREHANSGSSKHSAGYTNDKNYKTKKEENNTLN
GHIAKISNDDHKDNFSVDKDKQNQVMNSKTSFERPPSENNHLHHSKIETGENDPQVEVYTTGNKREDEQN
EKKLVNAEKVTNGVNSGEPKVSDGDDSKRSPSENMDMRNNTMSSTTCSTASSTTRNQVVNQTVKQVVGQM
ASQTQNIKDSGGNRTTGFIGHLKNIELAEKEIELLILIFKKYINQFTLVVEKEQTKNLEETANTKNKNEK
GAQITEEETINRSPSPVQEQVSDNVKLMNELGSGHVSENDKKKNEESSNTCGKWTAQEVSLLAKALKSYP
GGTRNRWEQISNFIKTKSVKEVIKKTKEMFENETLKNLSRNFEETAFDNFKNQNKGVMKKIDDNLDKRDV
KMTTDGEENLTNNSADNLTNNLTNNLNGHAEVKRPWTHQEQHLLEQALMKHPASLPKKERLQLVASEIKT
RTLEEVILRMKTLRAQILAKKAAK

***P. knowlesi* JDPs: Type IVs (2 Members):**

>PKNH_0941100
MKLFFFSFALVYLLVVNNVNCLFKNFISGFYCNDENCYGILGVSEKASVSEIRSSYHRHLMNMKNSYDLE
KKKRILKAYTVLANKRTRKYYDFFLKNPNSILNVIYLIFYYLFKLIKVIIALIIIGVLLCGFQYLNNKYE
MKRRVHKLSKNKAFKKEVQNRIGLKHPDFRTYEMAMKKKIEEDIEIEVAHEMGLTYKKKDEQFAFSDLII
VKFFILPKQIICYVLWNIKWLIKYHILNNEYDEGDKLYITRKCLNIPAYRWDALSDEDKKILLKKKLWVK
EIQDEFFEEQMEKERLNKISSAKYKKQMRMKKKGTSFNYND

>PKNH_1129500
MELVTWEDQVNGGEMESFRELSTRKEESIRSRSDRSEGAEAYGNTLWNISSMNSENKKYFLQNYVEKIKR
ISQYSVKPMYYDILSVRSNADGKTIRRSYLQLSKLFSVHKKLSREHEECYHCIQKAYQMLNDKFEKFYYD

VLNGYIHESTIEECRRQLQMEAEVIYRNKINEVKSIYLEKLQKEERKNGLIIEKALFGNLTLKEEHINNC
LNMDVITENDLEGPFLDLTTILQARIENSSFMFNDEFSFAHFCGIPKPLIKIPCRKDVSYADILQSTEMY
LYIKYKFLNTDHELIVVDRSRFTLPQSTHRIFGNRICGPFSPVNVIKMKHLSNSLLDTIFHFFSKNKFYI
TLLTTILLCAQSVKSA

P. falciparum JDPs:

P. falciparum JDPs: Type Is (2 Members):

>PFD0462w/PF3D7_0409400/Pfj1
MLALRILRRKVCSEHFLFERSFFTQSIKGKNGCLVTRYDKNKLLFYYKRNINTSRKCLNQDPYTVLGLSR
NATTNDIKKQFRLLAKKYHPDINPSPDAKQKMASITAAYELLSDPKKKEFYDKTGMTDDSNYQNHSSNFE
GAFSGFGDASFMFTDFAEMFTNMAGGNKNTSTRGEDIQSEITLKFMEAIKGCEKNIRLNVKVSCNNCNGS
GKKPGTNLTICKVCNGSGIQRMERGPIIIGVPCRNCSGNGQIINNPCKHCSGSGVKFQTKNITLDIPPGI
KKGMQMRIPNQGHCGYRGGKSGHLFVTINIEPHKIFKWVDDNIYVDVPLTIKQCLLGGLVTVPTLNGDMD
LLIKPKTYPNSEKILKGKGPCKVDSHNNGDLIIKFSLKIPEKLTPRQVELIEEFNTIELNLPNPQTNVKQ
KKNIYETKGNINENIFSMNNTYNNMKGPEGETSNTQAKSMKNQNWNNEKSVNNKGTISKDEKKLNMKNNH
INEKSNLKNSSHMDTNKNEENMSDDEKKKIKKIIPEPPMPHTHKIVNNLESKNSCNIPIPPPPPKSSSKP
ISENQNISNREHNGVTNNSAKLDNNINMNYSCDPYKNVTQNDLNNNDNIKNKIYKDNTNISNHHIFKNDN
INQQQFHCADNSSENNNESDMNTTSTFSFAKKWISDKLKPKN

>PF14_0359/PF3D7_1437900/PfHSP40
MFFSSGFPFDSMGGQQARRKREVNNNKFYEVLNLKKNCTTDEVKKAYRKLAIIHHPDKGGDPEKFKEISR
AYEVLSDEEKRKLYDEYGEEGLENGEQPADATDLFDFILNAGKGKKKRGEDIVSEVKVTLEQLYNGATKK
LAISKDIICTNCEGHGGPKDAKVDCKQCNGRGTKTYMRYHSSVLHQTEVTCNTCRGKGKIFNEKDKCANC
KGMCVLKTRKIIEVYIPKGAPNKHKIVFNGEADEKPNVITGNLVVILNEKQHPVFRREGIDLFMNYKISL
YESLTGFVAEVTHLDERKILVNCTNSGFIRHGDIREVLDEGMPTYKDPFKKGNLYITFEVEYPMDLIITN
ENKEVLKILKKQNEVEKKYDLENSELEVVSCSPVDKEYIKVRVTKQQQQQQQEAYDDEDHQPEMEGGRVA
CAQQ

P. falciparum JDPs: Type IIs (9 Members):

Exported Type IIs (3)

>PFA0660w/PF3D7_0113700
MATLRKSYVPEILYFSKFFMNACFISLLIITVNCFNYENFVCKDKGIYNEKIVIRYKRCLAEGNKNFFFN
KDNGVFGKSSMDYYTLLGVDKGCSEDDLRRAYLKLAMKWHPDKHVNKGSKVEAEEKFKNICEAYSVLSDN
EKRVKYDLFGMDALKQSGFNSSNFQGNISINPLEVFTKAYSFYNKYFSKSSGAGNHNIFTHIKNLYPLRN
DFSEDESSYNDVEEYEVPLYVTLEDLYNGCTKTLKVTRKRYDGCYLYYEDYFINVDIKQGWNNGTKITFH
GEGDQSSPDSYPGDLVLVLQTKKHSKFVRKSRDLYYRHIITLEQSLTGFDFVIKSLDNRDIHIQIDEVVK
PDTKKVIKNEGMPYSRDPSIRGNLIVEFDIIYPNTIKKEQKKLIKEIFKESY

>PFE0055c/PF3D7_0501100
MSILNKYEGKKNKIFLFIINIILFYTLEYVLIGSNYDKHNQSFGNEIFKNTKVFDFTSLRSLAEFNSGSS
RESSKTDETDYYAVLGLTKDCTQDDIKKAYRKLAMKWHPDKHLNDEDKVEAERKFKLIGEAYEVLSDEEK
RKNYDLFGQSGLGGTTTNDEAYYTYSNIDPNELFSRFFSHDASSFFSQGFDDFPSFQGFASMNSRRPRSS
RSNIFSRSFGRAASFEVPLQVTLEELYTGCRKKLKVTRKRFVGLNSYEDNTFITVDVKPGWSEGTKINFH
GEGEQSSPNEQPGDLVFIIKTKPHDRFIREGNNLIYKCYLPLDKALTGFQFSIKSLDNRDINVRVDDIIN
PNSKKIITNEGMPYSKSPSVKGDLFIEFDIVFPKKLSPEQKRTLKETLENTY

>PFB0090c/PF3D7_0201800/KAHsp40
MAIFKKYRFRENKIIFLFFIKIFLFSLFIWELCCFNKEKFQDQIQTSYYNKNNTSGNVSNLIIKRNLAQT
QRNFKSKNGKASTKKNEDYYSILGVSRDCTNEDIKKAYKKLAMKWHPDKHLNAASKKEADNMFKSISEAY
EVLSDEEKRDIYDKYGEEGLDKYGSNNGHSKGFKRTDPNDVFSKFFKTETKFYSNSPSSPNGNVLFEGSL
FGGSSPFSGINPRSGSGYTTSKSFSSMDKVEEYVVPLYVTLEDLYNGTQKKLKVTRKRCQGVTTYDDEFF
VTVDIKSGWCDGTTITYKGEGDQTSPMSNPGDLVFTIKTVDHDRFVRSYNDLIYRCPITLEQALTGHKFT
IITLDNRDIDIQVDEIVTPLTTRVITSEGMPYMENPKMKGNLIIEFDIIFPKKLSDEQKELIKEALGGNG
F

Non-Exported Type IIs (6)

>PFB0595w/PF3D7_0213100PfSis1
MGKDYYSILGVSRDCTTNDLKKAYRKLAMMWHPDKHNDEKSKKEAEEKFKNIAEAYDVLADEEKRKIYDT
YGEEGLKGSIPTGGNTYVYSGVDPSELFSRIFGSDGQFSFTSTFDEDFSPFSTFVNMTSRKSRPSTTTNI
NTNNYNKPATYEVPLSLSLEELYSGCKKKLKITRKRFMGTKSYEDDNYVTIDVKAGWKDGTKITFYGEGD

QLSPMAQPGDLVFKVKTKTHDRFLRDANHLIYKCPVPLDKALTGFQFIVKSLDNRDINVRVDDIVTPKSR
KIVAKEGMPSSKYPSMKGDLIVEFDIVFPKSLTSEKKKIIRETLANTF

> PFF1415c/PF3D7_0629200
MINRKVCLLFLAVFFVLTIFKKLLKWEIFIEAWYTQEETDDDYDRMKLYDVLGVDKNASSDDIKKSYRKL
SKKYHPDKAKDKNSNNKFSEIAEAYEILGDEEKRKIYDRYGLEAAKNMESNKMDEDPSDHFNIYERFFGA
GFKREEEIKKADSLILNIEINLEQLYNGEFFSVMYTRDVKCLRSDDCIERKKECSGKGYKTITQQVAPGF
IMQNKIKDDECIDRGKAWNKKCTYCPNGMKEEKTIELTLEIEKGMKNNDKIVFEKKGKQEIGYENGDIIF
IVQTKKHKIYERVNNDLHQIYEISLKDALIGFSKNLEHISGKPININKQNVTFHNEVLRVQNKGMPIKNS
NKFGDLYIKFLIQFPKQLTDEQKKVLADLL

>PFL0565w/PF3D7_1211400/Pfj4
MSRRVNYYEVLGVPQDADLTVIKKSYRTLAMKWHPDKNPNNKAEATERFKQISEAYEVLSDPKRRRKYDL
YGTDENYMADENDEFSNFHKNFGFNDAQRIFEMFFGDSSPFGNDSFFSDVMGSSFVDKRRGRVPRSNDPF
DNFFGSSFNVSFGSSFDNFMDGGSCFTSVETSTSNGGKFKNRVVKTSTSKSTSIINGKRVTRIETVKTLP
NGTVERTVTEREEDDRGNINIRQLPAHELRRNKR

>PF14_0137/PF3D7_1413900
MEEIILHMIIIAPLTKWLIGSNRRWNQGKREYGIYIALLVLFCVGIYELRKPNQNLYEVLNLNAYASKTD
IQQSFRKMSRIYHPDKNKEPDSLDRFNKIREAYEVLSNDKKKYTYDRFGDFGDSEITSFFYVEIIIIAMF
QFAISFIFGFLYTYGKDNEKYRILICLYIALNFCMELILRFSPESTVFLSFIPILSHYTPFERIHAFRVL
VPLIMNAILLVDIYYIEEDTEVYVSTFCEYVFENNSKSIKNYDDAVLFCARLVDGKMNNASNFSWREEKS
SLDITNMYELDDDKVFDKQYDKDDIFYSLLYNIIENNKNQIDLKIPKKELCRRFDWSRWYTTAILEKNTE
EKNFVESNATKGIIFSSVLYFIGLVSHLVSK

>PF11_0099/PF3D7_1108700/Pfj2
MNVTNVVKRRKKLSYFHSLLLIIFSFFLSCARGMDYYKRLGVKRNATKEDISKAYRQLAKEYHPDIAPDK
EKDFIEIANAYETLSDPEKRKMYDMYGEDYAQGGMGGGSPGRGEHAHGFHFDQDVVNEIFKQFAGGGGAG
ASGGRAGNFHFKFTSGGPSFNHFEDEYEDIYKNEVLKINSKNIESVLNDISFSLIINFYSPTCSHCISFK
KKYLKLRKKFDGYITFAVVNCQEENMLCRKYNVKSLPQLILMRSDKTYETFYGNRTDENLTYFIKNNIPS
AIIECNNQKKLDNFLTQNIEIPKVLFFISHNDNIVMLKALSLEFKKRINIGIIYNTNYSVMKLFKKKNIK
TPSLLLVDDIDSLSGDLTQLKNFDFNILSLKLSHIVAQNRLKNNLYGHVTSYQELTKKKYESGQCHEKDS
QICFFILKLLKKNYKSFDEDIKKVANKFSSDPLKILYINIYQQPYILDSFGLSNNIQYSNGLILVAFRPK
RQKFKVYDGDVNVENVHKFVDNVVSGGIPINQNIKRSLKFVHVEQYDDEL

>MAL13P1.277/PF3D7_1356700
MNYYKILGVTQNACKKTIREAYLKKVKLYHPDLNKSPDATTKFKQIQEAYQALYNNDKSNTSFYNENSYT
KNYRTEDMKKGQGFTNDFSDFHREFYEEVHRMREHERNEQNRRYSNNNYSYSYDIFNKYPREFFYINLIF
KLFPLFVVPFLFLFVVYKQYILKSHFKEKPILIYDAYGRAFLVDIHGRKFRASEFDKY

***P. falciparum* JDPs: Type IIIs (25 Members):**

>PFB0920w/PF3D7_0220100
MKYFKKFKYFLPKYILTNDDENKNKYASHKIYNLNNKYGNFLKLIICLPFILITVLWIFLTISIFNVCTY
DPTLACIVPLDSIYLRSLCESVRSKNSNDTIKEPVLKNKVFSLPNEKKLTKSEDICDNNVNCIFKFNEKL
INDLEKYKVSNENDVMAYVKSYSVYNNNNNNKKDDILDTKIHNIGKNGEDIIKTMEILWLEFMENEKEKY
YLLKGRLYKYNNKFKMENKYTDEYFPRKKWNNYNDLIYKGSKDLEEKLNKMFYEWYKQENLNLEEYRRLT
VLCRTGWKALYNYVENICKEIIHSDLDIIKNKKGSNMKKGLYNNEYKNNGKNIPFNTSSSIDNKKLYNSF
GKFENPMCFNYEDSLTTSCYIDENKSDSSYETEENVNYNNKMGKRKNLVESQIVGKSNNIEEGENVEYLK
NNKKIGDDEMLQDYEKEKLKKKKWTEKEEQTKKVNYSEKVNHSEKVNHSEKLNHSEKLNHSEKLNHSEKV
NHSEKVNHSEKVNHSEKVNHSEKVNHSEKVNHSEKLNHPNREKHSQKEKHTEKDDKRNNFKKNNDVLEIM
DIIRYDSSDEPENSKNIGKKKKKKKKNIFKNFENVANSRGSKNFKNVFSRNKYTLEEEVNSVCKDGFNKK
KVLIKVNMLSNSDDNTSISDDNSDTCVDRTYYDLLNVEPDASFDEIKHSYRKLALQYHPDKNINDPEANE
KFQKINEAYQVLSDENRRKMYDEGGMKATENMFFIDAATFFTMIYSSEKLNKYIGILKITTFVQILYENK
ISADKLDNSKDLIQNVLVNDQIKREVELAVLLKERLQPYVDGDENWVDNMRKEIKGLLDSSFSESILYSV
GWVYKNISSRYIKKMNSILGLKAVRGHMQAYLRCAENIYMGKLAFNKILQGFNLLSGLEGEELSMKLGDI
ICDALRLMLWDIESTVKDVAKRVLRDKAVRKKIRLKRAEAMLILGNLMLEISGISGIDFIHYKVDGMKII
ESALMKSIQFSENPEEN

> PFE0135w/PF3D7_0502800/PfJjj3
MNNDPYLILNVEKNCDIETVKKQFKKLAVIYHPDKLKHNLIKEKSDEKNTIDFVTLIWAYEEILKNIQNQ
NNNTCISQTYENALSIHRKDLEYIKEENTFIYFCRCGDFFIFNENLCYEYYVIYVCQSCSSSIYLIP

>PFE1170w/PF3D7_0523400
MDEPKNVSNSSNFGGEASYDNNQIVPKYNNNYETEKEEKNLISNIFSTRRPKHAGAGLVSGIKSVTKGII
VGTSFLFISPYLCAKAEGINGFFKGMFFGLLSAIIIPVVSLGVASYQIGRGIMNTPESITQKALGKIWDD
EKREWYDFYYNLDEEANELLNITDDNKNNKYDRKKDTNNDEYYNKNGNIKVKNDEFYRILKVPTDASQNE

IKRQYYKLAKEFHPDKCSDLKAKEQFQKIGEAYQVLGDVERRRRYDKEGKNAINNMQFIDSTFFFTLLFG
SEKLDPYIGKLRMVMYVEYEQLYKDEDVQRLIIKEQNKREVQLALHLRDMINNYIFGDPDDYIIKFSQQI
KELCQTSFGHIILENVAWSYENCANQFLGEKYSLFGISGKYYKMQQKKRVIGTGFKFVKTLIKTSSLANQ
IRKKEDDDDISYEKTAKVNKKIEDSLPTIVETMLNICLIDIDQTIKGVCKKVFTDMGVDENMRKTRAETL
IILAKIMKKIIYDFKKNTEIMDTKKLFEDACMRACQKQDDDYI

>PFI0935w/PF3D7_0919100
MLNDIILQVIVAAFGVAIVNSDKIKFLQKFKYATYILILSFLLYKGIPWKRENYYTYLNITPNATKQEIQ
TAYRQAAKIYHPDKNPDESANSSFIKLKQAYDVLTDDVRRSNYNRFGDYKNGEIDDNTATLLICLSLVQH
TMFFIIGYFLSYPKKLEFSRQIFLVYNIASFCFELQFRFIEDDTTFDWLPALGYLLPYEKIKFLRTFFPI
VFFISICASAYSYTDKNATLIFLMRSILSTNRIIVERSNDVIESTNYLKKNGEKLVTKLQQIRKGEASSQ
LNLKDDENLDKEYLENGDKKNATVDSKLLSNVKEFALTLDSQQISLLEKCFDLMKNKDANEKNKKKSWFE
FFSIQVIFGVIFVYIWLTSK

>PFI0855w/PF3D7_0917500
MAINNEEVNTMNETELKRIYYENFIEELNDEELMYKKTVEDLFYIYKSFGFIYSIIKKANLLLSKENFDT
FLKSLVSFIDELFLYNPSLFNNELNNGLNVPYVNKDNILPNKKRKYPEDTGSNDINNMNDMNDINNVNNL
NNLNNLNNMNNMNNEKTNEHTSFMNNMSKTYVEELIKDIKNIDDNFSETNCSDFLYIVKNILKMKNTSDI
ECVKNRFEKIKNVLNIGKSIFDKKDILYDMNDHKKKAHEYIDDMYTKLNNIEPIIISIIYKQNFWYNNIL
NKSEYDSINSILNLNIDVANEKIEEVYTEVLLLLSEEMTQNKDLHFAKEKLERLKTSYDAEIQRKLQQEM
ELTKKLDDKRKAVQFIIDRYNEYTWFDKNSPKKHSFYILGINPKNITEEQLKSFGKRLKHILHPDKEPDT
EWKKKAESAFKEASLAILGCQQEFKTKILKNLEPGPMAPYLTLIGIESTTNESNEKDNINKNKSSNLNDN
NNNNLNGTTGLHPYYPTQVYYPKCELKCVDQKIGTVLIDIKCPVQLGVIKKVILYVHRPIYGNEPMNLIP
DDTYLSYKKEHNVNVKNLNQSIEVRVDYVQPLEIASSTKYYIGIQLIAERGNTMINWNSINITLHKFSNK
NIIKKLLTSFKNATFINQAQLNNTLANDNKDEMTKFLNECIAAAKLWAQTV

>PFI0985c/PF3D7_0920100/PfJac1
MKKIKYLSILWKHKKINICNHKNCYTYNLAQNFFHSNKNNIDSNNYNGNILSNKNKVKHFKCPKCNNNIS
TDTVSFNCEVCKALFNIDIFRNFNIFELFNIEVNYDIDKSHLKKKFNEIQNIYHPDKNAQNVEVDEINEV
SSYLNNAYKTLSNDVERALYLLKMEYNYMISEDECMDDDEFLSEIIKINVEISKPDANIELLTKEYKQKY
EDYSKEIKLHFKEKNFNNILNVLKKLKFINRVLERLQNI

>PFL0055c/PF3D7_1201100
MQFFNKSYRSLLRILLDINNNVKEDYTYGAHVHYNKKKEKKNIRNEHNFLNSPIYISTIIYFIAICYFIL
LNTCIPNNNELYGEAYYYKDKRYLCEAETINISRSNVNTPLTSNFFSLLYNTSPCILDDVFENKEEYMLK
YIQEILGNLNKYISWDNYGLILHIDDYTPVTYNNKDIEIDKKIEMIWKKRQHRIEDIKDSWDEVMLNESS
KYSNTVNELKKYYDKLRYQKLILKRDYDDIWNDYSKFLFIIEKHMKTELYKIFNVWYYKKALYMNEYRKL
VNSCRIAWKALSNHLKYTLKMTIIYDIEKMKHIREADFKNAIMHLYLKKDDDDDEYSNKQGNEKRKKKKK
KKKEGFFHNFFCFLNDPYEIKEDEESISPNNDFNLSAVNYLECCRTARHIDKEKKTHSRKVDEETMTELG
NPIDTRMSERLKELREYEKRKKEKEQNNSKYVFDYGSFEEKSESEKSLDISKNFIFSEEVRSTNSGMHYS
LDIEADEVLSKNKNKERGMNYLLKENNYNNHSVNAVSPVNTTYYEILNVDTKAELKEIKSNYYHLSLQYH
PDYNIGDRIAKLKFRLVSEAYQVLSDDERRRIYNKQGLKATEKMFLMEPGLLFMIMFSIDEMSDYVGDLK
LFYFIKEAFEKKKRIEDIESPFEDMDAKMENDQRKREVVLALLLRERIQPYVDNNKEWMCEMEKEIKSLL
ESSHSNAILGSIGWTYENVATKYLSDIKSKWRLKEPMSKYDASFRHVNRSKRTKMTKLSSRFSGMFSCSS
ALKSQEPSMGETSSSDNDRNEGSEGICNMDNMFSEETLSLLEIDENKYFGFMTMHILTLILWDIEETTQY
AASRVLRDEGVDENTRIKRAEALQILGKLMQKWSLNVKSKKEIREKDIIEIMEKARAYNM

>PFL0815w/PF3D7_1216900/PfZuo1
MSGCNLIWLDKATKKRSKDILCLEDIPYKDEIKYLNELKVLPYVSCIHCVNIRKKKVENAGFMFYIKYEV
PLLLNEGDNIKKSKENKRVEDIEEIFLYKDKIDYMLNLEKSINKNNIYDKNISNDKENKRKKANEKDEEM
NKDDYDNKENNNNGADNIKGQFKKGSNLIKKCIDQNIDVYDVLGVEETDDLETIKSCYKKLILLFHPDKN
KGTAYLNEKEKEKEKKKGKNKKKKNYMNDANNNNNNNNNNEKDFLYFIEKYNIEKLTNDEKKNIFLKIQD
SYTILSDKILRKQYDSSIPFDERIPTLTQLEEAKNFYNFLRPVFKRNAKWSAIKPVPDIGDENTDIKEVK
YFYDFWYNFNNWRDFSYQNEYDYEQAECREERRWMERENKKIQKKASKTENLRIIKLVDLAYNNDPRIIA
ENKRIKLEKLKKKEQAMIEKKNQQNENNNNNIQHNNNNNNIPSHKKNIDKASVKLWKHHIKSLCLTKLSN
LVNTEDIQQKISIMSFDDLCEFIYDIYVILNFTVPKNNTDTTSTLLTNIKNNTIHKKVYNPNINDPKKAF
QSIGSTSTTNNNHVNNNNNNNISTDGKTSTGFIGHLKNVHLDYKQIQTLIDTFKKYIQDHSFILQNNDHQ
LNDQHNYQTDIQTGTYTQCNEQFNKNEKENENEIEKQQTHANNYSNVNTQDIRQNKFSNNILHSNNEKIY
EHVKKENEEINIYSNDSTQININESNEHVYEHVGKLNEDTLKYSNDSTQSNVNIYNEQNNQENELQSNKW
SAQEVSLLAKALKLYPGGTRNRWVLISNSIKTKTVKEVIKKTKEMFENDTLKNLGRNFDETPFDHFKNQN
KGVMKKIDDNLDKREYKLTKENNNQVETDNLNGDVEKKKPWTHEEQHLLEQALIKYPTSIPIKERLKLVS
HELKTRTVDEVILRMKTLRAQIMAQKSSK

>PF07_0103/PF3D7_0724400/PfTIM14
MWPVVMLLFGGGVLFVKKGLNYVKNQGIQLNGKRSFFPSGFNKNLNNLFLKNDLKGFERNMSKSEAFKIL
NINPTTNKEKIREVHKQLMLKNHPDNGGSTYIAAKVNEAKDILLK

>PF08_0032/PF3D7_0823800
MGRKASNDSYENSDVNGAVSKRKSKGKDDINDIMNDLNKKKMNEKKNMKDKKDGNFSGNMIGGMYKNKLR
YILDNIIIISIILSFVVLISFKYLEEKYSIESYFEDGDSFDYYEVLKCKRGDSIQKIKKNYRDLSKQYHP
DSNKNCKDCDKKFQEITKAYKTLSDSRLKKAYDNSKGKVLKLIESNSINLNMKNYVDLVENSNDYWIIQI
YSDTDSLCLSFSNIWEESFDKYHEYISFGRINISTDKKLIKQKVPFNVKIYPTIFILSPDGTYQLYSNIF
NATSKDFQNFITSNYPNNIYDLKMLNDALYKMSSLNVKKSGYIDKNHHVILLTNKKKLSLQAKQITYKFN
NIYKTYSIKYNEIDNISNESIKKSIIDSLKVLSIKKDEYLKENQNIDYFILVNNNQVKIIRRISPTNIKN
VYNDALKKNIVEINSINVDSVCSTMGSRKTYCYVIFVDNVDDEKNVNYMRKTYQNINSSYNKFVSKNGSE
ETEEQLFIQPVYVLKKNLTKKFNKFINDKTINKYDSFLLDYSSNTFSSINEIKNLSNYSQNKDDLSFLHN
IYKDIEILNFQKIPKYCLPFNINCLFNIKTTTPYKLYNIINRTSKLQIIFSLIVGFMLFPTFKEYGFLKY
VLFALSVGFSVIVINIKDFILLLAS

>PF08_0115/PF3D7_0806500
MSNWNFFNDYLNWFNGTTESTTFHNRSSKKGSNKSVSTNSTSINTNKNNINELSNSQPNNSGQTCDEMKA
SNDGFEKDVSKIKCAKKYNPFINIKEKYYKEYNNMNTKKENNKSEKKCGIQIEGQNGNPSENPSENPSES
QNENPIPCQIKKCDKENDQNIPKSGNKNKDTNFFNQLGLSNWLPNYDMPIYNEYKKGYYLDKEAKEVLSK
KGSKKSNVIKIGGDSSNSNMNDFNVSDCGNNTCVDTTYYDALNIKPTAKLSEIKTSYYKLALKYHPDKNA
NDPEAKLKFQKINEAYQVLSDDERRRQYNKYGLNATKDMILIDPSIFFMMLFSSEELSDYTGTLRIAFFV
QLAFEGNMSIEDKKSSNQVMINEMEVEQKIREVELALLLRKRLQPYVDGDVEWEEQMETEIKGLLESSFS
SSILESIGWTYENVATSYIAEVTTLWGVGATVANIQAAGRTIGNTFSAAKSMFNTVVTIKDFSLNSEKIN
SIKEKKDNLKTSSNNSLNNHSSSNKGSSEQNGTNENLKCDNNQVNHKNNVNTSDNINNVNTSNTHENKEE
EKKNTIIDKEENKALGVIIKNVLTLVLWDIESTVRQATEKVIRDEDVNIETRLKRAEGMKFLGKLMQKWS
KIKNDKCDTNDIDATKLLEKAIIKASKMPNDEDQGDQDNIKREYL

>PF10_0032/PF3D7_1002800
MRKKNNTKKIAEKTNETKSNNTHNKTTNYFNINEIKELYNNKNNDMYEKLFDNNNNEQCGRKKKRKKENI
TCENNNNLPSIYKKQIKQTNVICNNHNDNNKEIITQSESYHYLYNPSFIKRSKTNNEVNTLTKNKKNINI
NKTDQCSNNHFPYSNRIFDQTYKKSSSTYNINTRNINKKNKIINESNKIQNNCNINKNYNMNHSFNKYDF
IRFVCSPNKSKQKVNDMDSPKKMLETKECFKETNLSNTCTYQNNKIDDSICIRNKENQDNYKNCIIQNKN
KASTTTQDENITKQSPQMKCDKNIKMDKHVQAQKKKIQNCSNEDIPLIDLNDEDSINKLSKNFFINNKKI
FINDISKDKISKDSVNKYYEYGNISPYKLGSHYMDHLSCHNKEGKNISTTTTNSNNNNNNDDNNNNNDNN
NNHNNNNHNNNHNNNHNNNDNTIGTNIYKHHNNVNYNNNHNINDNIKINNLNDLNVHDNNYFPYDHTIHH
LDDNVNNTSDKIKTKKNLSTLKNVNIYESNVQPYSYNMFRDNNMENDKYNIYNDDNKNDLHENMNEYIKQ
NYKTTKSLYSYLDLSYNCSNEEIKKAFKEKIKVCHPDKGGNIKEFLQLKFSYDILSNKKKRKMYDKYGNS
ILELLISDHIYDYNISSDEKTEEEIIDEEYIKIYELFVQKYHNISYLHNLLDLKITNSQYNHFQKLIYHF
CNIDTNIFKNILYLYPIYSPNISTYNKKNTTKKNENLNNSTISFQEQTSQYSQETLSENSSSSLEATNKE
ITIFDTIEKKNFGNLFLEINKDFDHFYKNYLIHKMKSTNQKNEPQGINENIQKIYEHNGKDNISHNLYDY
NNEMETPLKSTPMKKKNIENVENIQNVENIKNVENIKNVENTQNVESTQNVENTQNVENIKNDAFFKDIQ
VSPVVYKIINEKNDKLMDDFYKWFEYFFDESENDKQPNTQQHYETNNIDTDNQRNKNNECNNTFYEYIPI
GEKRKNITNDYKKNDCTPNNHVPYIYEHVNKPNIKQTNYYVLDEKQSIHVNLSNIYQHDNNFYDLCNETP
HHSIKKSKSIKTPISYKKNIEEYQRIACSSFDKKTFHSSYNLHNKEHKYINHPYCTPIKTNITTNKLSPY
MYAEENSKNKTNHNHIYNCTLNVEEKYGFNLFKKSNHKIKDLTHDFNYIYIGNNINNQIHVNPNNEQNTN
KRKKIFFFLFIKQECINNFLKIKLLIHKMKEKKNNLKLISLFQNQIDKYMKNMEYILLLTTHKEELIPLN
DFYLLDKNIYTKRFYCIPIYIKKNNILIRPNFFHIKWIVYTLQSFIFYNFFFKKINKYMCTYPFFNYKYT
HFKNYINQHNQQQNNDENDIQTYNTNFYDYYIFFQHYIIKNKIKYVKHFPHHLMLNFKDLPSTNMKDYKN
VHIHISSIFVLTPNKIYPSVDNYMY

>PF10_0057a/PF3D7_1005600/PfJjj1
MMDIEKCLCYYKILNVDTDASVDDIKKSYKKIVLLYHPDKNTHLCDEDKKRYTNIFRKIQEAYECLTNEV
QRKWYDRNRKRIIEGRESSEEKNSGKQKYAYSYTNINIWKYFNNTCYNGFDDTEGGFYDVYGKLFDDIIK
EENEEIKIMNNIKGCHKNGNNHKKKREDNMNSSDKNNTQQDYLNNKNNGDDNKNNGDDNNKNNGDDNKNN
GDDNKNNGDDNKNNGDNNYNGDDNNFYFIKAPHFGNSSTCGKEIDEFYEYWSNFTTVKKVDYSYEYIKTY
EYENRNFRRNLKKVSEKRSIKEKKEYNENIRSLVNHIKKYDIRYINRIVELIEEKRKKVELRELKKKEEI
LKRKLLFEENKKKEYYEEMEEQYISSSSSSSLNVHKYNNECKDINYPFETNKNSLPNYTKDKQNVDITEP
KTQHINQKKKHFTDDDNISSVHDVKRNNNDDQHSMDSHNEDENNERVINKIIYRCEVCRKNFKSLKQYAS
HEKSKKHMNNFLKNACKYAMDNMFGDENREDHINDPKCRDNKNKKNNDDDDDDNDESESIHGDNNNNNKK
NKNDDDDDDDDNDESESIRSDYNNESESIHSDYNNDSDSICRNYNNDSGSIHSDDSNNNNDKDKEKNEIKE
KSDSECSSQSSISKENDDILWWYKNKKKNNYKDVLTSSYNNLEENNVDILNEKIKTDDSSDNYKSSRKKK
KKKKKNMNIKESHDNIIKNNNMNIINKVDEDNKMTSVHISNSKKLKENIKNLKCQICQQIFDSRNKLFQH
IQEKGHSAYKNVDTNIKTNRGKKKKK

>PF10_0378/PF3D7_1038800/Pfj3
MKCLKLFSFKSSKKNLHKNKLNSEYKNAYSCEDKTVKKYKRKVCETLFCKYINVLIWYILLYIIILNVCL
WNSISSYDIEFNYIYGRKLYSTESLNREKTNTEKTIISKVNLLPNEIILSNLKNENNKREESVLEFNEQL
IENLQKYKLWNNYIAIPYVKKYNPIKYNDIDNELNEKIDNVGKNGEDIISEMNNLWLQVMNNEKSKYLSL
IHRLHKYYYNIKVKYKIPNDYHNAKWKECYEIIKMGEEDIEKRLNKMFKDCFKQKMIYLEDYRRLTVACI
IAWKALSNYVQNSCRKVMSVYFNCIKKYNKNMYIDINQFNNNNINTKKYNTQLYEGKNFGKINFCECLIS

APYIDEDNSDALLSVEDIFCIELEKGNSKMENEELNMNDERENRGNPNGNMEETKQIKLCDIKNDNPHDN
TCINQNDIASNSSSDDTDNDINDDINIFHQSKKYSRIPRYDTSNLKGYNKNSYYLDLEAEWLFNSFLEIK
KLDEKIKNKKRRRNKENMNNPLSPDNSENVFVDKTYYDILNVNPDADFVEIKNSYYKLALKYHPDKNKGD
EEAKLMFQKINEAYQVLSDEERREQYDNYGKNATQNMFLIDGSFFFTLVFSSEKLCDYIGTLQISTFVKL
VHERGMNSNDLLHSMREIQNKLSREQDIRETELALLLRDLLQPYVDGDPNWEKRMEEEISSLIYSNYSSS
ILKSIGWTYKNVAKTFIKENKSFCGLGAEITKMKAEFRHINNCSKVTRSAIRLNSKIFKNIQDNKMLMGN
LSLMNNNKITDGNYRTFDNDSMNDEGKSSSKNDVIVYDNNTTEILKEKSKIVADILDDIFTIVLCDIELT
VRYAADRVLRDEGCNKEIRLKRAEGIKIVGNLMNKWAKIKKQQMKDNKIDITDTIESALHVSRIRSRNQK
D

>PF11_0273/PF3D7_1126300
MKQKKMDFRKNKNNNDSNNVIIINDEINSDISLESSEEEVDNYDQHYDNFLTPKNGNGNRVLNHNHIHNL
KSKKNNIIIDLTLSDNSNKDYVEYQNVKKNCEDNFKEGDNPSNISVSTKEFIDCYKNDKVKNIKVNEKQI
EQNNTKNQESQKNIHINNTSFMKISCNEKNKNEMINEFKINEHDNISIHSVHTIEEDTVNSNEDINFNHN
KNNCMNFLHNEHNKRNYEQMMEHNIRNEKNKRHIIIENTKTHKITSPCINLEDITQPEHLNQTPQNKKKY
YRNINNNVIDLDKENEQTKDFSFYKNDHSSSNKNRKKNNPNNAYHANNAYHANNAYHANNAYHANPFKSN
MFRNILFNKKYRIEDLYYIKLKYDDLSNIEKKEEENVKNFYNKYYKCSFYVDKNINNICLKKYDICNYCK
HCLKFLKFLIIHGNKYKGNLINIFFIYSDIQQLIFTYKCEKKHLFNISLFHVIHNLWCPHDFCLFQCKGS
YSKNYATEFFRLKELESMEKQKRLFLQAKVFCLFNSYGTLPVNKKNVESEYTNDIDRIIKNANNPWEVLQ
MNTYTKLDISDKTELKKMARKNYHKLALKVHPDKNKNDNASLAMNILTNSMQSIMSI

>PF11_0380/PF3D7_1136800
MISNKDDAHECFNLACKYMKNGNFSHAKNLFLKAKRMFPDIDITEKLKTCEEEINKSEHIGTEKTTYNNN
TTTSTFNRDTTNNVHERFRKKDECLEKILRTNNYYEILGIPKNSNDEAIRGAYKKLAKLYHPDKNKDKGA
EEAFKKVSKAFQHLINKEKRYEYDNNIDEHGQHTTYRTTHYYYNDDTFTPEDLFRNFFGLNFATCNNTNF
RTHINSNRSYSNNNNTNSANNAQRNYSFVQILIFLIMFLIFFLSSHFEQPKAVYSLQKTNYFDTINYTSL
NNIRFYTKRTFNYNYPRNSHSRFQIEYEVEYKYYEHECHILTKKIKNDYYREKNKYQQKINYKDIPESCM
KLKTLEEQYNNYILKLKKR

>PF11_0433/PF3D7_1142100
MNDKKNSINKSGRHSFNRNLKKKNIYKDNNNVYNYSEGNKKIFKYMNDLKYSSYNNLNDGHSDKSSSNNK
PSRKKNLLKKKRNEHFNKKKMNSTNSDHHYDDEDEEDDDDDEEDDDVDDDDDDDDEEEDDVDDDEEDDDD
DDDEDDDDDDEDDEDDEDDENDDNENENDNENENDNDDDGENDQYRHKNNMNTNNNFAINKKNNHIDVDD
GDYKNSYFYNDLDKSNKKNKVNLKNYNSDYYDIYSNIYSYYDIFTEEKTDKKKFKYFNDVFSNFDKKYEN
NNNEYNNDKYNYDMNNKRKVQEKYLQQPTVRTSSTIEKSNTSPHYLSQKNKNLLSKSYGREHNINDEQKI
SSNINEENIYNEYYDDGMYNHKRNILKKSASANMMDNQNVKNNYINKKINKKRNENKNHKDINQNIHQDV
HKNMDDDDKAYVHINYDEKGDIHKMNNDYHNNIYNKIYGNNNNSDRNNNHNNHNNQNYYANEFSHVKENN
CEYTDVDETSNVFNNLSDNYIKNKNIRKQKRKKNKKKTTLSDNEKATSDLSSIHNKKINNNMNNMDNMKR
INNMNNMKRINSMNNLQSEDLNNDPMNQQDYDKKYTYSKEKSESNISLDSSCSFKWEAHLEKEKMNDGMQ
KNIKFLKNPTKKRMYKLKKIVEILEYTIYNEEKQGSKLEGKLYDYVVLTKNLKEKIINLENINTNNKNKM
QSYEKDITKLKEQNEEMKLTLSTFENELSNLINKFDKNFAKELIDTKSQNDVLKKEVERLKGEIEKKNSE
IKKFENLNDLSKKLQNEYMDHAVPCDVTASNVENKTDANLENNQNGPFSNNNISTSFNGVYKDNATEVTN
MNNNNNNNNNICNNNNNNICNNTCNNNSNNSSNNYICDNSYHDGKTNNYTNYIEQDLFLNELPFQYGCSD
SIEYNIVDEFILKIKSSRMSYDKNIEKYDDQTKSVIYGSYISTFIIENKDICNHKKILKLLQEFCLVESK
FLHIHLCLKKERCDNISQKILEKEVIRQLENNEFDKNKERISVISPVILLLIFKNMNIINLYKIFISCLK
CDNYRWLRIIKKSLFIKFFDGTLFDTKIKITDGSFVREIKLINLLLNGNVPDEYIFSENMNNNNNINRDH
CINSCVVKQPLNLNQLNMVNYKMKHPLFYCSNEQLKRFVYRMYLNFTKEPINVFNGDNIYHYVLQKKNLD
VLKILKLSGKHYNYIFEKNNINKTPLDYIENEDIRVDLISGYILDIAGKGAENYKNSNYNIAYDLYTEAL
EKQIKLSESVKVGKSMNENIGKLYYNRARTLMHLNKWMDVIENCNNCLKYIPKYVNAFDTQIQAYEYLLE
YDNALLIYKNMCLKCNIKPDEKEEKLKAQINATSFQVLNINKNCSISEIKQAFSNLSKKWHPDKLGIHIS
PDIKKRHNNHFKRLFKAKQMLLNEVDRIKEKKKKETNYIYPQIIEDININKNNNNNINQTNTKSTDTNFK
HMNNNNNNNNNSNVVYNTRTNNPYYYNVQPNKSNLNNTQNINKSPSYEKDKHLNYDQHDDKTYKYEPKKS
SNHFMQSNENHIYNNSASDINKNVNNDNSNLPNVNDIRNNIFSTYKDDIDKFQGKLKSYNNINNNNHIFN
NNVEDSNKHLDNKLSNLNNDKVDESFYKKLKEEYEVLNYEELMNLKTKTFNSINNLILTEVNLKREYQEL
CNKEKNNVIMNARLCILQEIQKALCVRLDKERKLKVIENIIKNRQEDQSSIYSSRNNHLNNSNNNDNHFV
KTNIDESHVHTNKSDEMNLQADRPNQQNYNALNKNKSTTTNEFSENIFNDQKEHVPLSSNMDSNKGNTYV
DEEPIDSTKNNRNDNISNKNVHKKDIQNINQNNNIVGSDQGDDIFFKNNNNNFGEGQDIQNIHHNVYNNT
PTDQNKKEYEEESSGFNNIQHDNTMKSPVDKHMDHNNVPINEWPGDAHEKELNNIFVNKIKNNNDAQSYK
GINNKTIFLEENNDERYNQENEKEKNYTENKEYLKRNENHNNIEEESFIHLSNIKGSTTNSNIMKMGSNL
NTSNNMNIFFNQHNEEEHEKEQEREHKSEKEGEKNKNEKGGEKNKSEKGGEKNKSEKGGEKNKSDDLFKT
PPSCSFRKSNTINDKNMNEVNTYLFNSNHYNDNYENIKENTNNYTNDYTNDYTNDYTNDNINNRFIPSDS
QTKEDYNYDKYRKNEDINNVPIINNENIFNNKFNTNDHSNNIHLDNSFNENFHNLKKKNNEEGNLNNDKE
NINSNIRNAHYDYTKSFINMKNNEYNYDEQYNYDEKHNYDEHNNDEQYNYDENNHDEHNYEYHMHDKTNS
FPNQIKKNTYHFNKKDEEFKNNDMTNFNDEENNQIFNNKNHIHNQVDSQDDLLSSSRMNNSNLLKNNKNT
DKNNFNNNNNNNIFYRDTNNNSSSNHLNYNFTETKIKNNLNISNVMYSNDNQKVIINNDNFLNLYKENST
NNKMMNNNLESNFKNDIDRSSPSLINDNKVGDQKKSMFYIKEEKNNKQTYVTYDNNNDDDADEEEEEEED
NDNNKNVQEQHYINKEMTSNSEQYDNNNYDDANNNNNNNNFRQSFSLNNIHSNHMNNYQNHNNIFNNDDQ

SFTYDDGDYYNLKTSNYAYENNKLRSFSIFPNDNEQINSDIYSNDRNTSFNNLDNDINAQPKTNYSFNDN
MQKNIYQKKNSQNKMFSTYEQNENVDKINDFPEDNKKKQTQDSIQPKKGFKKFFSSDDKKKLIDQNNKKN
KLKTKDKK

>PF11_0513/PF3D7_1149600
MKSYSKLYNKYVPIFSFDNNENKCKKSVLLKYLNCRYNFVLIFGIILYIFLLNINIYDDRYGSEYIIIFN
RNLSETSSSISSGNKNERLLEINNFTNTQKKVNTYDKGLNSLFKKSEKYLEKFINNDMSKRSSKSKTDVG
SKEPIIYNNIGGSKMSGTKSNVIESNVSSTITKSLERNDSNTNIQEKVPKESKNTRKDVRTKNVNTSKRN
EGLNDINESEKTRTRRPSTKSNNNNVSYENELESVSESFDNFLEDHMKEGVFLDGEKKTSHRKEVDDEKE
HNRYDNKEKKKKDFDKRRTKFSSSDNRYKYADTTYYDILNVSPDADSSEIKRSYYKLALEYHPDKNPGDE
EAKVKFQKVNEAYQILSDKEKRAQYDRMGMQCVEDMTLIDPSLLFMMLFSSEKLCDYIGVYDLTYMFNFI
MKSMNEEHGGGLMFNMLGLMNKFFDKFKKDQEDREFDLAVSLKYRLEGYVNGDDDWEKQMENEIEDLLES
NFSGHILESVGWIYENVGKCYILKNTTFMGWGARSAKKEYKKRDRMNDKRVFRSIFNTMGMIARFVLNPP
PFMLEGQYMNYNNMGQITNNENTSNSCIVCSSSNRGPHGVQNINGLGNYSNAMAVETYIRKIFDSLMSTI
VTLFLSIIEGTVRTSCKMVLVELDVDKDTLFKRAEGMKLLGQKMQKLAKRKKEKSAAKEMNSMDIIEKAI
KEAKLRMESYREEN

>PF13_0036/PF3D7_1307200
MEEGKVGNSNEDFPEKEMNIDTNNNMKDNTHEDEKNNPSEENQLNNNNNDDDDDMNELLETFLQDIDNIT
SNKEKNVEEKKLNKGDAGKEINRILEHKHSSPFEIFGIYENINMDLIKSRYRKLSILIHPDKCKIDKAAE
AFHILTRAYEELQKDEIKEQYKSVYEIAKKNIIKKHQLKKKKINEINEYLNKTEEEYEITKEIQQLINEE
CENLLKIQKEKIEYAQNCKLANMKYVQEKEEERLKEEMEKEKEKKLWEQGRDERVNNWKNYKKENLKDEK
EFHLYKNINKKKQERTQQEEQKLKNVPLTYEQNNVHKKRKKN

>PF13_0102/PF3D7_1318800/PfSec63
MSQFRDTFTKDSKKEPLLSYDDSAFIFFAGTVLICVLIPCTYVYLKNIIKRIFKNDYYNLKKSKNNSTYR
SCTCSLCKQKQEKRNKNTRIWERIGYIKIIQFFLLVLFWGLLYILINQMLNTKPMQTFDPFEILEVSIGA
TVGEIKKAYRLKSLKYHPDKNPNDTSAAAKFILITKAYQALTDEISKENYEKYGNPDGPGMMKVGIGLPK
LLIDEKYQLLILSIFFLIFLVFIPATFIIYYQKQKQYGPNGVKIETLQYLTYTINENSRSKSFPEMLAAT
AESRDIEMKKDDEQYIKTLMEELIEPKKRTFKIPVITKNYFLILAHMQRRHDLLSEDLKKDLEHILKFSL
LITHSMIEISILRDWFLTAQSALTFRRCLIQAFEAKNSSLLQIPHFDENIVRHVHKGKFSVKEVLDFVHQ
DHENRKGLVDMNPDQILDVKSFCNTIPDIKMTAHIVVEDETHIVKGDVASVYVQIDRSNLKENEAAGYIH
APYFPQPKFEEWWIIATYKNDDRILKYVHVKNCEKIIEEKLQFLVDKVGNLSVSVFALCDSYFGCDQKVD
IPFKAYSKTEIKREIFVHPEDIELDNEPTLFQQMLGDINKKRVSSDDEDDDDNEQGKNKNNSNNKVMKTK
ASADQENYVPEDDANDESDDD

>PF14_0111/PF3D7_1411300
MDIFSLASNLQQIAIGGVNYVNNKLLKKSLNYETNYITYIVIGENEKTPYDINMFFLPFELSTKLRFKDF
KKYFPFKGNIIFRFKIALCDMINVINEGIINNSPLLLREKNIHHNIPHNNNIISDENEIKNILKQDNFNY
VWIDITNDEAFIPTFNGSVIVKVLFVNHENYKEYNNIYFKNYNYSHKTYHINESHLCSYIPLKYENKIKK
NSSTDDNIYNDDILIHNKRNHCETIKRSYDHDNLVNFSYTNSSDEFQRVNSDEHCKKGSDKNLNLQQNDL
HKSNNYYQNGVYTYNKYASNEHINISNNVNNNNNNNNNNKLYDEQNTKQEDNSKEYNYQYNYNNSNIYNY
HDSHQVLERRSNSDNLNTLHEENKIINNSQSNYIPSIHISSLNCNEIDPHNKYISTKGAIHNSRSNLLTN
QTKNIQNDFNNKTQMFKENENITNKINNRLQELKEYRDHEQAKFKEKVVINNQIKKQINKWCKNSDNTYK
DIKVLLTTLNEVLWKNAQWKNVYMADLISNPNVVKSAYKNAILLCHPDKNRNTTTEQELRAEMIFQALNN
SYKDKRNL

>PF14_0213/PF3D7_1422300
MDLSFIPKELRGKDIYKILGLHINDCNNENAKNIIRKKYLKAALILHPDKKEVHHEQKEEKKVYAENFAT
LKSAYEFLLNENLRKKYNLYLEKKGKVVNNTPVNNTSLKRYLDKKKFLSFNLEKLHLKKKLEEKEKIAQR
INENNSISKKKKVIINKNKNDNHYNHEQHHLKDIKKQNESFMKKNKSKHFYKKQREIVDQNEKIIEVYLK
NYQQNVKLLQSYIETKNILKFFINFNFKRYSLNFINDEQHDTNLDKNGDKNDDGNKNDDKNDDKVKVGYL
IFSHRFETIRAYLHYKNNMNEINKNFILKLRVPCDQKKNKNCKKKNGNEDEDEKDNIDKIMNEMVNELDK
IFTT

>PF14_0700/PF3D7_1473200
MSSKQDVDLYEILGVNRRAGIKEITKAYRILALKYHPDKFLTNFKKSGDAKDSKDATNQEQNGVALSSEV
VIGSGNTAKMENQNTGNDVNSGNSENAEKSEQSEKVEKVEKAEQSENSGKSENSGKSEISGKSEISGNSE
NARNSENFGNSEISENSESAERSRIPEDCPTPENTPNSENSVNVANSEFAENHVVVVEQLNDQERNQEEG
STNCSNVVEEEMTLEKCKEMFLQIQKAYEILKNPVLRENYDFYGLDKDLDEFQTYYEPRLFHSRINVKDI
HKYESFYKGSAEEKEDLMYFYEKFHGDLNNILEFIPFSESTDLNRFIGIFEKSFDDGEIVKTELYEKSLE
NVEKIIKKYESLLKKEHSEIPKNEDKKKSKRKKQDSLEELIVAIRNNEERRNLKITNLLTSIEMENKNKN
KRRKKEDFPTEEELNKIKKKLEENKKRNEQARRK

>MAL13P1.162/PF3D7_1330300
MFLIKKRFLSFYNIISTSPILNKKFVYVKNNYIFQKRHFSNKNFYDILNIKKDSNKNEIKQAYRKLALKY
HPDRNPNNRKESEQKFREITEAYETLSDDNKKKMYDSQLNNGFYSNNFNNNYYNTTSNSNHMNYNYQSKR

MTDEEIENVFKNVFGNMNLNDIFKSNIFNENSFSSRTMGSDIFSNFGSSASYGTPRNENIKQTNIKTEII
PRGNKIIEKTTKIITYKDGNVKQEIIEREISNNSKEFEDFVDFDFLYKNNNLYNNMNSQNKINKQSFHRV
INNYKQNRLVRNVLNYCYGILSLATRRILVNLVIHVIRKVIQTIIFMLRKK

>MAL8P1.204/PF3D7_0831200
MFDYYKNKYKKGCLPYFLVIEIFSVIILGFIYVTFLDTRPYIGNSFIATDFFDRWGRYLYATERFNEIFG
IPSKDVLRSRSKIERYEEDDVILINRFVDEDEYNAFLYIKKLMKDLKKYKLWNNYSVIPHVNEYNIVNKN
EIDERIDNEIDTIKIFSKSNKNIMYDLWFKVMNNEKMKYYSLINILKNTYKELKIIYKIPRNLRKRRRNK
CKNIIYMYGDFMELMLNEMFEDWFNEGDFFLDEFKLLINSNRIAWKALMNHIQCTCKNIMTEGLEEVIRK
KNEKRSSNNIKKSSEKRKRRNRNENTKCAEKKNIDNYKEKVFLLKYNQYLSFNYKDSVDTELNMDEKEKR
FKVKRRKHKKGKLRGTHMENENMKECMKQKEKNLNETLLNNPDLYKSESNNIKSNNIKSNNIKSNNIKSN
NIKSNNIKSNNIQSNNIKSNNIQSNIIQSNTIQSNNIQSNNIQSNNIQSRKSRNLEEAESIFSSETRFNN
YNKKKVYYLDEEAHELFSNKNSKQKDNNNDNAYGGCSRSRSSCVDTKYYDILNVKPYASFKEIKDSFYKL
ALKYHPDKNENNIEAKIMFQKINEAYQILSDEDQRRKYDEGELNEVNDAFFMDPLIFFMMLFTSEELFDY
IGTLRIATFVSLVFKHNFFANGILTTKNIINKGIEKEQKKREVELAILLRERLQPYVDGNENWAENMENE
IKKLFVSPFACSILESIGWTYENVSKRYIDEITNKWGIGLSFVNIKLAYRTVRAVYSHVKSFFNIIFTVK
KLKRAYEIMDSIIDGKDVHEPISNESNNMICDSECSCDYNVITPDNINKDSMSSNFNNNHSEHENNRKNV
SEACSIISKKNNINDLNDNNSPITYEEKNKILKSFIIELLTVILYDIETTVRNASDKFLRDQGVDVYMRL
KRAEGMCILGKLMQKWVKTKKNDQDINKFDFINHTKNAFDKASCVDVDEDD

***P. falciparum* JDPs: Type IVs (13 Members):**

>PFA0110w/PF3D7_0102200/RESA
MRPFHAYSWIFSQQYMDTKNVKEKNPTIYSFDDEEKRNENKSFLKVLCSKRGVLPIIGILYIILNGNLGY
NGSSSSGVQFTDRCSRNLYGETLPVNPYADSENPIVVSQVFGLPFEKPTFTLESPPDIDHTNILGFNEKF
MTDVNRYRYSNNYEAIPHISEFNPLIVDKVLFDYNEKVDNLGRSGGDIIKKMQTLWDEIMDINKRKYDSL
KEKLQKTYSQYKVQYDMPKEAYESKWTQCIKLIDQGGENLEERLNSQFKNWYRQKYLNLEEYRRLTVLNQ
IAWKALSNQIQYSCRKIMNSDISSFKHINELKSLEHRAAKAAEAEMKKRAQKPKKKKSRRGWLCCGGGDI
ETVEPQQEEPVQTVQEQQVNEYGDILPSLRASITNSAINYYDTVKDGVYLDHETSDALYTDEDLLFDLEK
QKYMDMLDTSEEESVKENEEEHTVDDEHVEEHTADDEHVEEPTVADDEHVEEPTVADEHVEEPTVAEEHV
EEPTVAEEHVEEPASDVQQTSEAAPTIEIPDTLYYDILGVGVNADMNEITERYFKLAENYYPYQRSGSTV
FHNFRKVNEAYQVLGDIDKKRWYNKYGYDGIKQVNFMNPSIFYLLSSLEKFKDFTGTPQIVTLLRFFFEK
RLSMNDLENKSEHLLKFMEQYQKEREAHVSEYLLNILQPCIAGDSKWNVPIITKLEGLKGSRFDIPILES
LRWIFKHVAKTHLKKSSKSAKKLQQRTQANKQELANINNNLMSTLKEYVGSSEQMNSITYNFENINSNVD
NGNQSKNISDLSYTDQKEILEKIVSYIVDISLYDIENTALNAAEQLLSDNSVDEKTLKKRAQSLKKLSSI
MERYAGGKRNDKKAKKYDTQDVVGYIMHGISTINKEMKNQNENVPEHVQHNAEANVEHDAEENVEHDAEE
NVEENVEENVEENVEENVEENVEENVEENVEENVEENVEENVEENVEENVEENVEEYDEENVEEVE
ENVEEYDEENVEEVEENVEENVEENVEENVEEYDEENVEEVEENVEENVEENVEENVEENVEEVEENVEE
NVEENVEENVEENVEENVEEYDEENVEEHNEEYDE

>PFA0675w/PF3D7_0114000
MHSMKERRYYSIYKKDNKMEDLGNKNKNKIHFISVSLMNCLSVIMFIILYIISLHIFFYKTNSFHLSLRE
PVPYANRTYNRILYSTEKKKVNRREQIRTQREPQKSNYKASMKNYLKCVKSAPYIDDAKYGALISEEEER
EMKMIKKMELEELEKRDELMEKRRLRRIERMKKKEEEKRIKEEEERIKEEEKRIKEEEERIKEEEKRLKE
EEERRLKEEEEERRLKEEEERRLKEEEKERLKMLEEDKLYKEREEQKKNKLNVEEEVVTFERLKSKKMDEK
EDTVVEKKENVEDQKDTEEVREEASEDKGDIEEVGKEEASEDKEDTEEVKEQEVSEDKEDAEEVGKGEVS
EDIEDKEEEGEKEVSEDIEDKEEEGEKEESEDKGDKEEEGKKEVSEDKENAEEVREEEASEDKEDAEELG
KGEVSEDMEDKEEEGKKEESEDKENAEELGKREVSEDKENAEEVREEEASEDKEDAEELGKGEVSEDIED
KEEEGEKEESEDKGDKEEEGKKEESEDKENAEEVGKEEASEDKEDAEELGKGEVSEDIEVQEEEGEKEES
EDKGDAEELGKGEVSEDKEDKEKEGKKEESEDKEDTEEVKEEEASEDKENAEEVGKEEASEDKEDAEELG
KGEVSEDKEDADEVEKEETSEDVRDNEEVGEGEVSEDIEVQEEEGEKEESEDKGDAEELGKGEVSEDKGD
AEELGKEETSEVKGDAEELGKEETSEDKEYTEEVGKEEASEDKEDTEEVKEEEVSEDKGDIEEVEKEETS
EDKGDTEEVREDEVSEDIEDSDEVGEGEVSEDIEDSEEVGKEEEPYEKGDYEEVGKEEASYDKGDADEVS
EDIEDYEELGKEEEPYEKGDYEEVGKEEASYDKGDAEEIGKEEASDDKGDSEELGKEEEPYEKGDYEEVG
KEEASYDKGDAEEIGKEEASDDKGDSEEVGKEEASDDKGYSEEVGKEEGSYDKGDAEEVEDEDEEHIRSK
EEVKEPEIGTDIEEENKEKYIKKLDVQDTLYEINVLNGEDITSFLENKNKIIQNEGEEDDDDDEDDEDDE
DGEDGKGVYINIVNNVPGNIWNDTIYDDNYNNNTYSSAEEYIFYEKKLDDNTRNNMKRSYYDILDVKEDS
DINEIKRKFYNLSLKYYPKMNKDKNLVMNQKFENISEAYQILGYENRRKLYDLGEYDETNKMIIIDPLIF
FNLIFTSDMMYEYTGNTQVSTFVKLFFEKNISVEDISYYVGEIMKEMMEGQNIREEKVAELLKDRLDLYI
DNEDEWKKLMENEISMLLKSSFSSFILESIGWTYENVSNIFLEEKVNSGKNKKGIYLKEANERIIRNSIV
LRQCKSRFISIITNYYPFKEQNNPFIKQAQYVSSSNYVLDDIINNIDYSIDNIHRAIDNLYYEHILNLLE
EEKNEILEEILRNILKIILCDVETTVRRSAQKVLQNAEGDTNLMLKRAKGLQSLGKMILQKVN

>PFB0085c/PF3D7_0201700
MKCKRNVFFSKSLKFGHISVFIFGILYGVINKLFISDVNSCYSVSNSIIYERQLSEKDNLSNSLEQNGEP
VVIGQFFSLPNGKSISISDDIFIDEEQSIVNFIDNIIEGLEQYMLWNNYMVIPHMKQYPPVFNNDKDIEL
NNKVDNLERNREDIIIETEKLWLEIMKNEKNKFASLKCKLFNQYNKFKNKHNIPKEQYEKGCNLCKKLIE

IGEKYLELKLNSVFYEWYDKKVICVEDFKRKIERCRIAWKALSNKIQYLCNKIIINCLDKIKYMNEMKIM
KAKKKAVKVVEKPEPKKKQEENLSMVEGLNCFEENHKIICIKNNDLISGCENVDTQGCPSVNEIINSSSI
NYYEKMRDGLYHDDEEYDALVTDDDLIFEMFDENKEDDIIEESENNESDEDDLLVEESESNESDEDDLLV
EEYENNESDEDESIIEEYGEAQEEVAISSSEVVDDEFTTNEDIESEERYSLDKEANRLLFKNDIYNIWFS
DLSNIYVDTTYYDILNVYPTSELSEIKSNYYNLALKYNPESNLGNAEALTKFRDINEAYQILSLDQRRMN
YNKYGLNATKDMFLIDPSIFYVKMLSIEKFYDYIGTTQIESFLKVLSEKNIALHELEHRLEDIMNLMYEQ
QEVRQVKIALYLRNKLQPYVDGDDQWKKHMEEEVKKLNKSIFGTFFLKSIGWIYTNLTQCYREDNGHSFG
VNLKLANMEFENRNKKNQLKVSKSMRNLLSIIKEYIPRNENITGLVKKIEYLKSENDIENNISNVNEKSS
SNDNSSDDENQNENENENQNENENENENRKDLKLLSDNEKRKVLHFMIKNIKNVVQGDIELTIRYAAEKV
LFDEGVDKETQLKRVEALEILGNIMKTCSKENKNWEKDQEADIENIIEKVINVSKMVNNE

>*PFB0925w/PF3D7_0220400
MTLICGIPYMFLMVVCVNKLYAFFAYTFDERHQRNLYTAECLIKNKESYSLEKNDSSSIDNYYKSIQNAP
YIDEDIVDNYKGELKELIKINKNDISNEINKNDTLNDLKRSDEFHNRNELHNREGSESKYVSNISAKEMT
NQDNCRKSSHNKKRGYSLEKELEKLYRIALNNNNNINIDNNTNIDNNTNICVDYTYYDILNINANSKLEE
IKEKYYEVASKYHPEKNIGNDKAFKKFELINSAYQILSNEELRRKYNSDGRSKMNNTNLIDPFVLFMLSY
ISINMSEYVGKLKIEYLIEESFETNSNFYDLLLSNKIMNNYLNVEQKIREVELALLLRDRLETYLEGDEN
CIVPIKNNIRAILEYSFSFSIMNFVGWLYEYFSKLYMGYNIELSLMNDNKGIMENLFRNIVKKEMHKNLL
NKNNVNITKDSDDFIIIDEKDHNNENIKNCTVLFNHIRSNNENNINLEDMTRNVLILIIILDIKLVIKKAV
ERVLCDKGVSQLTRKKRAKGLMSLGKEIQNYTQKIRDKDYKIINENTNILESIIEDIKKYMEIDKMNFLK
EKGKKEIDKIFYFVGNNIYRNKLKRNINEKCRLLKFLKYMINSTEE

>PFE0040c/PF3D7_0500800/MESA/PfEMP2
MEVICRNLCYDKKNNMMENEGNKVKKVYNNSSLKKYMKFCLCTIICVFLLDIYTNCESPTYSYSSIKNNN
DRYVRILSETEPPMSLEEIMRTFDEDHLYSIRNYIECLRNAPYIDDPLWGSVVTDKRNNCLQHIKLLEMQ
ESERRKQQEEENAKDIEEIRKKEKEYLMKELEEMDESDVEKAFRELQFIKLRDRTRPRKHVNVMGESKET
DESKETDESKETGESKETGESKETGESKETGESKETGESKETGESKETGESKETGESKETGESKETGESK
ETGESKETGESKETGESKETGESKETRIYEETKYNKITSEFRETENVKITEESKDREGNKVSGPYENSEN
SNVTSESEETKKLAEKEENEGEKLGENVNDGASENSEDPKKLTEQEENGTKESSEETKDDKPEENEKKAD
NKKKSKKKKKSFFQMLGCNFLCNKNIETDDEEETLVVKDDAKKKHKFLREANTEKNDNEKKDKLLGEGDK
EDVKEKNDEQKDKVLGEGDKEDVKEKNDEQKDKVLGEGDKEDVKEKNDGKKDKVIGSEKTQKEIKEKVEK
RVKKKCKKKVKKGIKENDTEGNDKVKGPEIIIEEVKEEIKKQVEDGIKENDTEGNDKVKGPEIITEEVKE
EIKKQVEEGIKENDTEGNDKVKGPEIITEEVKEEIKKQVEEGIKENDTESKDKLIGQEIITEEVKEGIKE
NDTENKDKVIGQEIITEEVKEGIKENDTENKDKVIGQEIITEEVKKEIEKQEEKGNKENILEIKDIVIGQ
EVIIEEVKKVIKKKVEKGIKENHTESKDKVIGQEIIVEEVKEEIEKQVEEGIKENDTESKDKVIGQEVIK
GDVNEEGPENKDKVTKQEKVKEVKKEVKKKVKKRVKKRNNKNERKDNVIGKEIMKEDVNEKDTANKDKEI
EQEKEKEEVKEKEEVKEKEEVKEKEEVKEKEEVKEKEEVKEKEEVKEKEEVKEKDTESKDKEIEQEKEKE
EVKEVKEKDTENKDKVIGQEIIIEEIKKEVKKRVKKRNNKNENKDNVIVQEIMNEDVNEKDTANKDKVIE
QEKEKEEVKEKEEVKEKEEVKEKEEVKEKEEVKEKEEVKEKDTESKDNVIVQEIMNEDVNEKDTESKDKM
IGKEVIIEEVKEEVKKRVNKEVNKRVNRRNRKNERKDVIEQEIVSEEVNEKDTKNNDKKIGKRVKKPIDD
CKKEREVQEESEEESEEESEEESEEESEEESEEESEEESEEESEEESEEESEEESEEESEEESEEESEEE
SEEESEEESDEEKNTSGLVHRRNCKKEKKYNNGELEEYYKEKQNEEYFDEEYIIQSKEHNTLNTFPNMAL
NEDFRREFHNILSIHEDTDLMELKRILYNLFLEYNPHMNNKQKAELDKKFSEMNVVHQILNYEERIRMYE
ENAARGRLNTVILDPIITFNVIFGDDTMFKFIDE

>PFF1010c/PF3D7_0620700
MNTILTVNKTNELGEKNEKNESLYEPSSDIDNNIIILNINSMNSEHKKYFLNNYISKIKYMRSSYSKPKYY
EILNVNVKSDAKTIRKSYLALSKLLSVNKKLSREYEECYYLIQKSYKILTNKFEKFYYDVLNNYIDENTI
EEQRYMLEKEADIIYANKIEELKDIYEIKIKEEQNKNGLIIEKALYGDLSLKEECINNCFNIESISEQHL
QGPYIDLTKILQCKVENSSLLYNDDFSFAYFCDIPKPLIKISSKQTKKKLYSHILQDTEMYLYIKYKFLN
VYHELIVVDRSNFSLPQSSHRVFGDRISGPFSPVNVLKMTHISSSFKDNILKFFSKNKFYITLFTTIVLC
AQSIKIKMNKLE

>PFL2550w/PF3D7_1253000/PfGECO
MLLKTSLKNICFLLCVVAFVYNNVGRKRVLSFNINNVYSRILSDLEERESESYNNNSNLKYNDSEFKYSF
GYYNDDTSGMSTFNGKDIISYLEEKNDNVLDDYNEDDNSPDMDTDDPVESYYDLLNVDKYGDLSELKNNF
YNLSLKYYPKMKNGKLLELNKKFEELSEAYQILSYKIRKEIYDNEGISGIEKMNIIHPLLYFNGIFIFDM
MYQYIGTTEIGYIIKIFLENNISSENIPSFREEMNENIMEYQIKREEELTELLKKRLDLHMDNDEQWKNV
MENEINLLSNKSFSNFILESIGWTYQNVANIYLEEIENVGKIYRGIYMFQANERINKNEEMFDNSRNHIH
SLINSFYPYNEQINPFLKRAQYNRTNVECITSNRENKMNSEYDALYNENVNNISDKVKYNLLNDLLISIL
YINVYDIEETVRNIAEVVLRDNDVNVNTRSKRAHRMRLLGSMILQKINV

>*PF10_0381/PF3D7_1039100
MLYFNLSNMNMSKKRLNKNNVNYKFEFINLFDKNGKNKRKITSWKFCKSLILTTLGMLYIFLLKVDIGTI
KNCTSKNFTHKKRRLYENEFFNNYNDEFSNENYNKVGEKYFHCIKTAKFIDDDSSDVLVKEHDNNIKELE
NYNLFESDEINSNSNYKEGEKLNQIDEISPSVIRNIKDEGYNIDDNSYSLNFNLDYSVDRTYYDILNVNV
DASLNEIKNNYYNLALEYFLDKNTNDLKRKMEFEKISEAYQILSDKEKREKYHKEGLDVTKDMFIMDPSI

LFMLNYSLDQLFPYIGKYDITTIINFVTDQFTRGNIFETLIGKSSLEKYGDLIRKMDEKEEERKNKLVLF
LKDRLQEYVDVDEDTWIIKMENEIMGLLESKFSSYIIESVG

>PF11_0034/PF3D7_1102200
MLYFNLSNMNMSKKRLNKNNVNYKFEFINLFDKNGKNKRKITSWKFCKSLILTTLGMLYIFLLKVDIGTI
KNCTSKNFTHKKRRLYENEFFNNYNDEFSNENYNKVGEKYFHCIKTAKFIDDDSSDVLVKEHDNNIKELE
NYNLFESDEINSNSNYKEGEKLNQIDEISPSVIRNIKDEGYNIDDNSYSLNFNLDYSVDRTYYDILNVNV
DASLNEIKNNYYNLALEYFLDKNTNDLKRKMEFEKISEAYQILSDKEKREKYHKEGLDVAKDMFIMDPSI
LFMLNYSLVQLFPYIGKYDITTIINFVTDQFTRGNIFETLIGKSSLEKYGDLIRKMDEKEEERKNKLVLF
LKDRLQEYVDVDEDTWIIKMENEIMGLLESKFSSYIIESVGWVYENVARAFIGKEGKVMSMDEKKARKQA
KHREQMNRKEAMIWSFRTVSSIGYILSGEPKNHLMIHGVTCNMYNYNEMNNFYNYDDKLVCHYPNGYSIN
KMFHRIIHTFVKTMTVYFLDIIESIVRVVAETVLYDESDDIKIRLKRARGMKKLGILMQKVARIRSEQFG
EDKVDF

>PF11_0443/PF3D7_1143200
MRIHCFSFVLLYFLFVNNVNCLLKKVLHHFYCNNENCYDILGVNEKASLDEIKFSYFRLLKKVEKNHDRE
KKKRIVKAFNVLVNKSTRKYYDYYLKYPNSFLNLVYLNMYIFYKLFKIICILLLIGLLLCVFQYIHNKYE
LKRVIQKSSKNKAFKKEVQNRISSQHPGFMNYDIKKKKKIEEQIEEEVVQEIVMINNQKTKKLLLADLII
VKLLFLPKQLWFYIIWNIKWVIKYNILNEDYDEHDKIYITRKYMNISMDKWNTLNPEEKKNYLKKELWMK
AKQEEFLQEIKERDRLNKISSAKYKKQIRMKKKGLSFNYND

>PF11_0509/PF3D7_1149200/RESA3
MKPYSSYSSAFSKQYMGTKSVKAKNPTIYSFEEEKQNENMSLLKSLCSKRLVLPILGILYIILNGNFGYN
GSSNSGAQFTDRCSRNLYCETLPINPYADSENPIVVSQVFGLPSEKPTFTLEGTPDIDHTNILGFNEKLM
TDVNRYRYSNNYEAIPHTREFNPLIVDKVLFDYNEKVDNLGRSGGDIIKKMQTLWDEIMDINKRKYDFLK
TKLQKTYSQYKVQYDMPKEVYESKWGQCLKLINQGGDNLEERLNTQFKNWYRQKYLNLEEYRRLTVLNQI
AWKALSNQIQYTCRKIMNSNISSFKHISELKSLEQRAAKDAQEEMRKRAEKQKKKKSKRRGWLCCGGGDN
ETVEPQQEEPVQDVGEHQINEYGDILPSLKVSINNSAINYYDAVKDGKYLDDDSSDALYTDEDLLFDLEK
QKYMDMLDGSEDESVEDNEEEHSGEANEEELSVDENVEEQNVDESGEQQSDDESGEHQSVNEIVEEQSVN
EIVEEQTVDEIVEQETVDENVEEQAVDENEEQQTVDENVEQQTIDESQVQEEISTIQENIEEVVSEVQQD
SEVDRTLHVPDTRFYDILGVGVNADMKEISESYFKLAKQYYPPKYSVNEGMLKFKQISEAYQILGDIDKR
KMYNKFGYDGIKGVNFIHPTIYYMLASLEKFAFYTGSPQIVTLMKFLFEKKLTVNDLDTKSEHLSKIMGV
YQKERETYISENLISRLQPYIDSIRNWDVQIKDQIYELMGSPFDIAIIDSIGWTLQYVSMSHMKNPKKAI
KKLETRSKKNKETVAYENNKLMNILREYFGNNEQINSITYNMEYNTLNENNENGYRKILNLNHKKQKKLF
EEIISYIVNISLSDIENTVKNSAESILTVEGLDEKKLSKRIESLRMLANAIRKYILRGKKGKKYKNKDAK
SLSGNIANEINLINKELQNLKEHTQANIPEHIEENVQENMEENVEENVEENVEENVEENVEENVEENVEE
NVEENVEENVEENIEENVEENVEENIEENIEENAEENVEENIEENIEENIEENIEENIEENVEENVEENI
EENVEENVEENAEENAEENAEENAEENDETPQEHNEEYDE

>*PF11_0512/PF3D7_1149500/RESA2
MKQHSSYRMYFSKKYLYIRDIKAKNTTLYFFEQEKQNKNKSFLKILCSKRFLVPILGILYIILNYNFTYS
GNSTCRLQFTHRCSRNLYGKELSIKPYLNSRYPIVISQVYGLPSEKPTFNLECIPDIHYTNIVGFNEKLM
NDANRYTLLNNYEVIPHVKEFKPLIVDDELLEYNQKVHNIGRNGEDILTAMQTLWNEIMDINKKKYTVLK
AKLRKKYHKYKIQCDMPEKAYVYKWRQCLNIIRKGGHNLEQRLNKQFDTWYKQNALYLEEYRRLTVLNQI
VWKALSNQIEYSCRKIMTSDITSFIRINELEIMEQRAEKAAQEEMEKREHICFSCGGDIQKNEPLEHSLK
NVKEHEINEYGDVLPSLKVSINNSAINYYDAVKDGEYLDHESSDALYTEEDYWFDLEKQTYMDVINMRKE
EILQSQQNQRKVDNELEVDKKIYLPDTRFYDILGVDINADMNNIDKSYFKLAKEHYPINGSTIDDLKKFK
EINEAYQVLGDIDNKRKYNQYGYNGIKNFNFIYPSLFYFFSCLKKYDYYTGTPHITSILKFLFEKKLTMD
DIETKSQYILRVMNEYQKEIEATISLRLIDKIQGNIDGIKSWDARIISEIKKLESHFSLPILDSMAHIFI
NVSQCYIGNQEKAKKKIQNRFKKNKLKSSCAYNELRYTLREYFKTRKQVSSLSYTLENNNNKKYNQNKWY
KNITNLDDKNKYKILYSFVKNLIKIALSDIEYTIKTVCENILTEKGIDDITIKARAESLNKLGYIIRQNI
LKGKNIKKIIKCDSRNIAANIVREINTINELLK

>PF14_0013/PF3D7_1401100
MSNRQSIDDMNLSVKNKKSKSIFSCRPVKYVCLSVAVAAVGYANYMNNGDRNSLSCVDLNNVYSRNLSES
QENHNSSFTRTNLVENENEEDDIFGVHRDESFAETVLNGQDIISYLEHQNTKIHEDNIHDDISHTLLNEG
DVQNYYDVLNVNEHSDLNELKRNFHNLSLQHYPKITSDNSFELNDEFNQLSEAYQVLSYQIRKNIYDNEG
VYGTKKMAIVNPLIYFNGIFTTQLMHEYIGTTEVAQFVQLFLERNIAPENIVSFLEESVSDMMKGQDYRE
LQLTELLKQKLDLYINDDEKWQNIIKSEINVLTKSPFSKFILEAVGWTYENVGNIYMEQTDNFDNVYHGI
YVNLADERINRNYAILDENVNDFVSLLKKFYPFTETVNPYLRRAKHNLNNLQGGINNLYSSVNVVYDNLF
NENINISSNEHYHLLQELLKIILNINLCDIEETIRECAYNVLKDKTVDASVHSKRAHRMNILGSLMLESS
NE

* The original records in NCBI for these PfJDPs have been removed, and the genes are referred to as pseudogenes encoding hypothetical proteins; the protein sequences listed here were retrieved using their old accession numbers (PFB0925w / NP_473113.2; PF10_0381 / NP_700854.1; and PF11_0512 / RESA2/NP_701357.1) as previously published (Botha et al. 2007. Int. J. Biochem. Cell Biol. 39: 1781-1803)

P. yoelii JDP: Exported Type II:

>PY17X_0216500
MQMRNNKNQKNHPSNAIIPYVKLLLFFYIIYIQKYTKSVINEKNAYENILGNVSGLRPQRVLTEYGRGKT
NIFQRIHNEDYYDILGVTKSADLDQITKAYKRLAMRWHPDKHTDENDKLYAEEMFKKISSAYSVLSDERQ
RKIYDTYGIEGIKGNVDSFKPFYHTEYFNKIISPLKNFSFMTLINDKYNEISNFLHNIEYKPFSSSKTRN
NNIPGSREITLDLTLEELHQGCRKEYKIVKNVFVGGTPFQIDKVLTIDIKPGLNNNDLITFHGEGDQVSP
SSLPGNAIFKISTKKHDTFIRRGNNLIYKHHITLEQALKGFNFSVRSLDNKDIIINVDDIVGPNSKMIIP
NEGMPCMDNPNNKGDIIIEFIHMYPETMSEEEKAALRDIINSTNNKNTSS

P. berghei JDP: Type II: Potentially Exported Type II:

>PBANKA_0214800
MQMGNNIYQKDHPPNGFIPYVKLFLFFYIIYIQKYTKRVINEKNAYENILGNVSDLRPQRLLTEYVRGKT
NIFQRLHNEDYYDILGVTKSADIDQITKSYKKLAIKWHPDKHTDKDDKLYAEEMFKSISSAYSVLSDEHL
RKIYDTHGIEGIKGSVESYKPFYYTEYFNKIINPLKNFSFVTLLNDKYHKLSNFLHSAEYKPFSSLKTRT
GNNNPGLREITLELTLEELYQGCKKEYKIVKNVYVGLTNFQIDKTLVIDIKPGLEDNALIMFHMEGDQVS
PSTPPGNIIFKIFTKKHDTFIRRGNNLIYKHYITLEQALKGFNFSIKSLDNKDIIINVDNIVSPNSKMII
PNEGMPYMDNPNHKGDLIIEFIHIYPETMTEAEKIALRDIINSTNDKNTSY

P. chabaudi JDP: Type II: Potentially Exported Type II:

>PCHAS_0213300
MENNKYQKDHPHNGFIPYAKLLLFFYIIYIQKYTESIINEKPTYENVLGSVSDLRPQRMLTEHMRGKTRT
MQKLHTEDYYNILGVTKGADLDQITKAYKKLAVKWHPDKHRDDDDSRVYAEEMFKNISSAYSVLSDEKQR
KIYDTYGVEGIKGTMEAPKPFDHTEYLNKIINPLKNFSFKSMINDKYAGLSNFLHHAESKSHASPEIGKV
NHNKAGSREITLELTLEELYQGCKKEYTIVKNVYVGVTHFQVDKTLVIDIKPGFDDNTLIVFHREGDQVS
PSSPPGNITFRITTKKHDTLTRRGNNLVYKQYITLEQALKGFDFTVKSLDNKDIIINVDNVVSPNSKMVI
PNEGMPYLDNPNHKGDLIIEFVHIYPETMTEEEKMALRDILNSKNNKHTYH

H. Sapiens HSP70

>HSPA1A
MAKAAAIGIDLGTTYSCVGVFQHGKVEIIANDQGNRTTPSYVAFTDTERLIGDAAKNQVALNPQNTVFDA
KRLIGRKFGDPVVQSDMKHWPFQVINDGDKPKVQVSYKGETKAFYPEEISSMVLTKMKEIAEAYLGYPVT
NAVITVPAYFNDSQRQATKDAGVIAGLNVLRIINEPTAAAIAYGLDRTGKGERNVLIFDLGGGTFDVSIL
TIDDGIFEVKATAGDTHLGGEDFDNRLVNHFVEEFKRKHKKDISQNKRAVRRLRTACERAKRTLSSSTQA
SLEIDSLFEGIDFYTSITRARFEELCSDLFRSTLEPVEKALRDAKLDKAQIHDLVLVGGSTRIPKVQKLL
QDFFNGRDLNKSINPDEAVAYGAAVQAAILMGDKSENVQDLLLLDVAPLSLGLETAGGVMTALIKRNSTI
PTKQTQIFTTYSDNQPGVLIQVYEGERAMTKDNNLLGRFELSGIPPAPRGVPQIEVTFDIDANGILNVTA
TDKSTGKANKITITNDKGRLSKEEIERMVQEAEKYKAEDEVQRERVSAKNALESYAFNMKSAVEDEGLKG
KISEADKKKVLDKCQEVISWLDANTLAEKDEFEHKRKELEQVCNPIISGLYQGAGGPGPGGFGAQGPKGG
SGSGPTIEEVD

Figure S1: Phylogenetic Analysis of PkJDPs

Phylogenetic trees were generated for: (A) all PkJDPs and selected *Plasmodium* JDPs known or predicted to be exported; and (B) all PkJDPs, all PfJDPs and selected *Plasmodium* JDPs known or predicted to be exported. The phylogenetic trees were created using NGPhylogeny.fr (Lemoine et al. 2019. Nucleic Acids Res. 47(W1):W260-W265). The protein sequences used for the tree generation are listed in Supplementary List S1, and the PlasmoDB Accession Numbers for each sequence are indicated in the trees. The Latin Numeral following each accession number is the JDP type (types I-IV).

Figure S1A

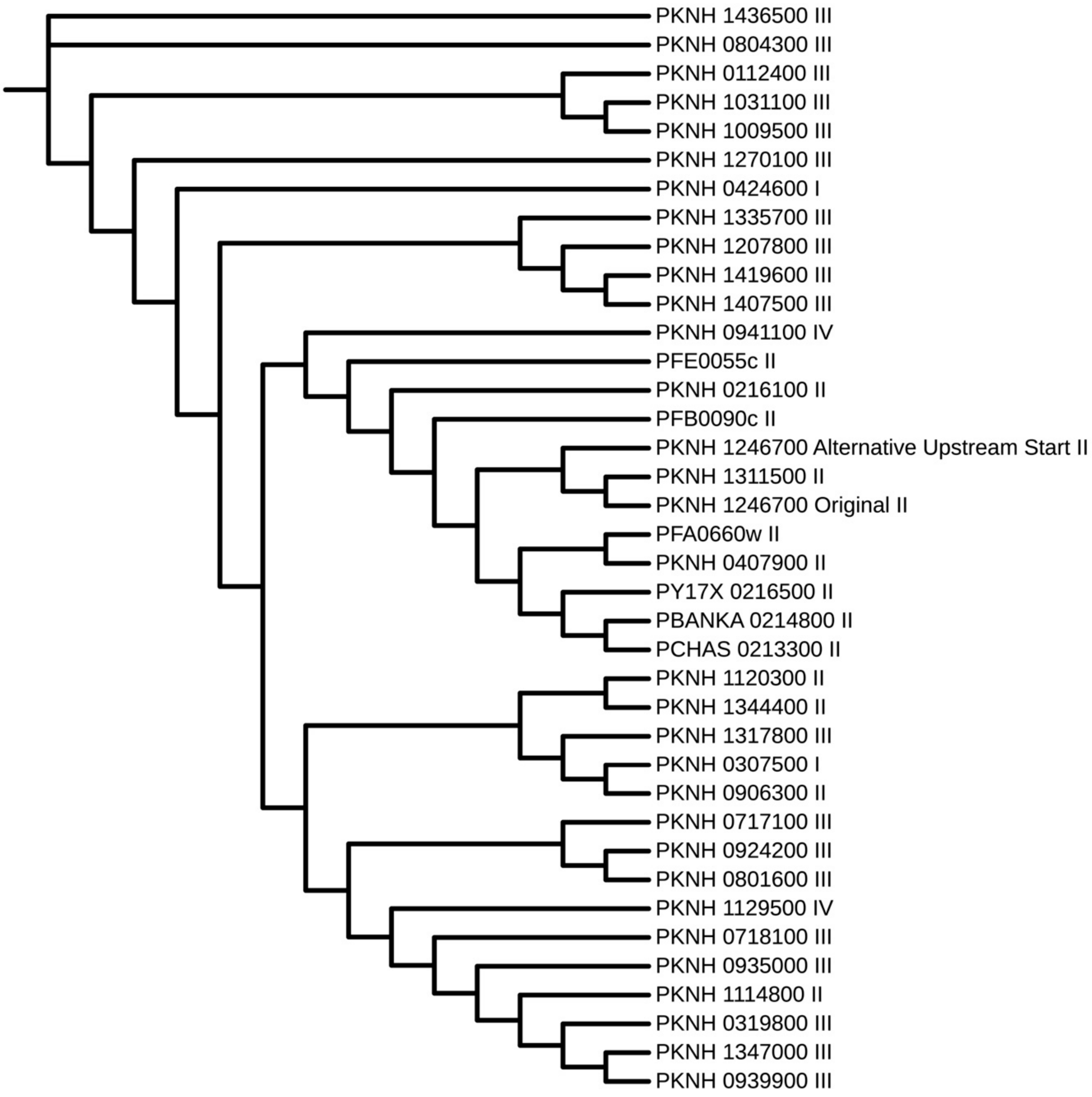

Figure S1B

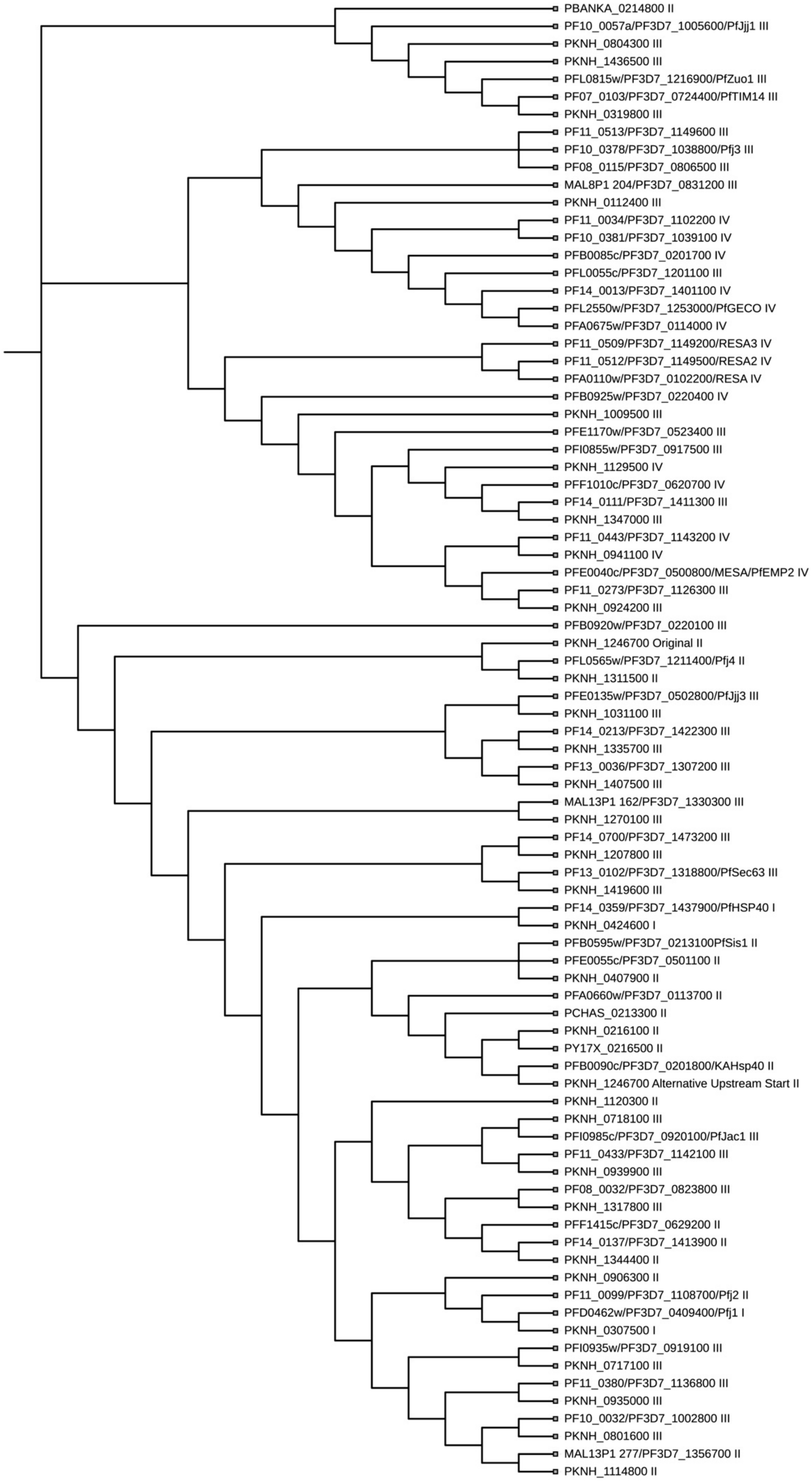
PBANKA_0214800 II
PF10_0057a/PF3D7_1005600/PfJjj1 III
PKNH_0804300 III
PKNH_1436500 III
PFL0815w/PF3D7_1216900/PfZuo1 III
PF07_0103/PF3D7_0724400/PfTIM14 III
PKNH_0319800 III
PF11_0513/PF3D7_1149600 III
PF10_0378/PF3D7_1038800/Pfj3 III
PF08_0115/PF3D7_0806500 III
MAL8P1 204/PF3D7_0831200 III
PKNH_0112400 III
PF11_0034/PF3D7_1102200 IV
PF10_0381/PF3D7_1039100 IV
PFB0085c/PF3D7_0201700 IV
PFL0055c/PF3D7_1201100 III
PF14_0013/PF3D7_1401100 IV
PFL2550w/PF3D7_1253000/PfGECO IV
PFA0675w/PF3D7_0114000 IV
PF11_0509/PF3D7_1149200/RESA3 IV
PF11_0512/PF3D7_1149500/RESA2 IV
PFA0110w/PF3D7_0102200/RESA IV
PFB0925w/PF3D7_0220400 IV
PKNH_1009500 III
PFE1170w/PF3D7_0523400 III
PFI0855w/PF3D7_0917500 III
PKNH_1129500 IV
PFF1010c/PF3D7_0620700 IV
PF14_0111/PF3D7_1411300 III
PKNH_1347000 III
PF11_0443/PF3D7_1143200 IV
PKNH_0941100 IV
PFE0040c/PF3D7_0500800/MESA/PfEMP2 IV
PF11_0273/PF3D7_1126300 III
PKNH_0924200 III
PFB0920w/PF3D7_0220100 III
PKNH_1246700 Original II
PFL0565w/PF3D7_1211400/Pfj4 II
PKNH_1311500 II
PFE0135w/PF3D7_0502800/PfJjj3 III
PKNH_1031100 III
PF14_0213/PF3D7_1422300 III
PKNH_1335700 III
PF13_0036/PF3D7_1307200 III
PKNH_1407500 III
MAL13P1 162/PF3D7_1330300 III
PKNH_1270100 III
PF14_0700/PF3D7_1473200 III
PKNH_1207800 III
PF13_0102/PF3D7_1318800/PfSec63 III
PKNH_1419600 III
PF14_0359/PF3D7_1437900/PfHSP40 I
PKNH_0424600 I
PFB0595w/PF3D7_0213100PfSis1 II
PFE0055c/PF3D7_0501100 II
PKNH_0407900 II
PFA0660w/PF3D7_0113700 II
PCHAS_0213300 II
PKNH_0216100 II
PY17X_0216500 II
PFB0090c/PF3D7_0201800/KAHsp40 II
PKNH_1246700 Alternative Upstream Start II
PKNH_1120300 II
PKNH_0718100 III
PFI0985c/PF3D7_0920100/PfJac1 III
PF11_0433/PF3D7_1142100 III
PKNH_0939900 III
PF08_0032/PF3D7_0823800 III
PKNH_1317800 III
PFF1415c/PF3D7_0629200 II
PF14_0137/PF3D7_1413900 II
PKNH_1344400 II
PKNH_0906300 II
PF11_0099/PF3D7_1108700/Pfj2 II
PFD0462w/PF3D7_0409400/Pfj1 I
PKNH_0307500 I
PFI0935w/PF3D7_0919100 III
PKNH_0717100 III
PF11_0380/PF3D7_1136800 III
PKNH_0935000 III
PF10_0032/PF3D7_1002800 III
PKNH_0801600 III
MAL13P1 277/PF3D7_1356700 II
PKNH_1114800 II

Figure S2: Contact analysis of the predicted 3D structure of the complex of the J domain of the type II PkJDP, PKNH_0216100, and human HSP70 (HSPA1A)

The contact analysis was conducted with LigPlot+ (Laskowski and Swindells. 2011. J Chem Inf Model. 51:2778–2786) on the interface between helix II of the J domain and the underside cleft of the nucleotide-binding domain (NBD) of human HSP70. The molecular elements are indicated as follows: the J domain bonds are shown with purple lines; the NBD bonds are shown with brown lines; carbons, nitrogens and oxygens are shown as black, blue and red dots, respectively; hydrogen bonds are shown by green dashed lines with the length of the bond (in Å) indicated in the middle of the line; and hydrophobic contacts are shown as brick-red spoked arcs.

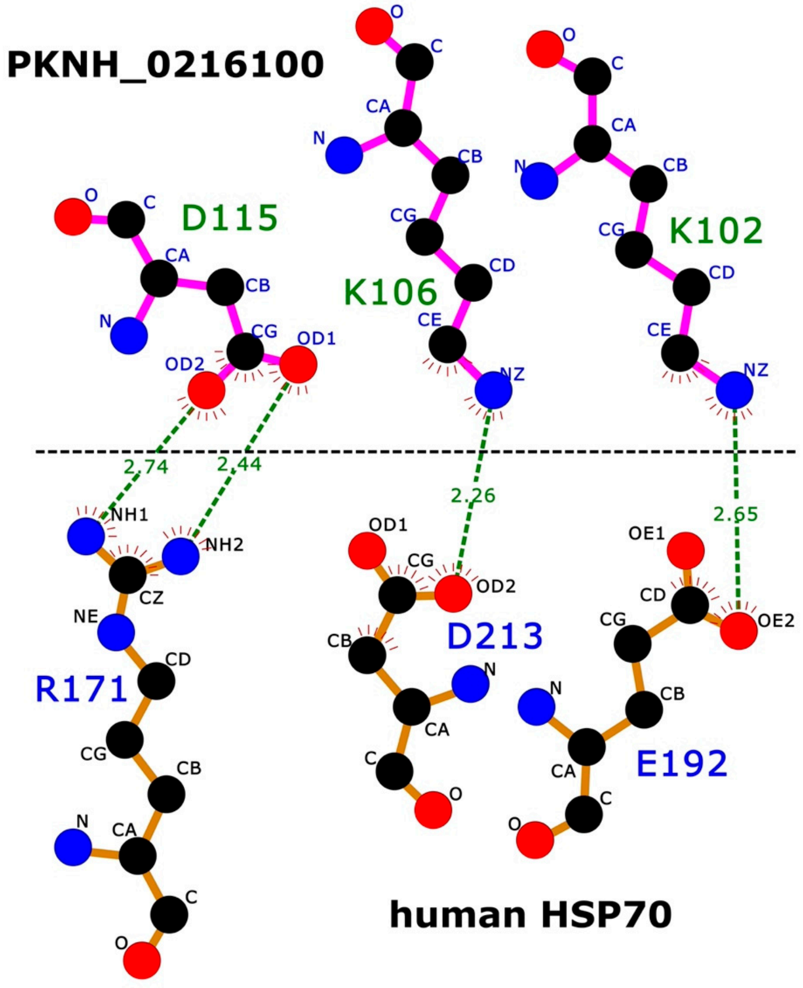

Figure S3: Multiple Sequence Alignment of All PkJDP J Domains

The alignments were created using Clustal Omega (Sievers and Higgins. 2018. Protein Sci. 27: 135-145) and rendered with box shading using Multiple Align Show (Stothard. 2000. BioTechniques. 28: 1102–1104). The residue position of the C-terminal residue of each J domain is shown in the final column.

```
PKNH_0718100 NFFELFGLQATY------DIDKGHLKQKFNNIQKLYHPDKHAQNEQLEQINEVSS-----
PKNH_0924200 DPWKVLQIKKISKMELCDKELKREARKNYRVLALKIHPDKNKSKNATLAMNILTNSMKAI
PKNH_1129500 MYYDILSVRSNA--------DGKTIRRSYLQLSKLFSVHKKLS-REHEEC----------
PKNH_0319800 EAYKILNINPTT--------NRDRIREVHKQLMLKNHPDNGGSTYIAAKV----------
PKNH_1031100 NPYSILKVEPNC--------NIETAKRQFKKLAIIYHPDKLNYHRNKERKGVEAN-----
PKNH_1114800 NYHKILGVTKNA--------CKKTIREAYLKKVKLYHPDLNKS-PDATSK----------
PKNH_0935000 NYYEILGIPKNS--------NDETIRAAYKKLAKLYHPDKNKE-KGTEEA----------
PKNH_0717100 NYYTYLNITPNA--------TKQEIQTAYRQAAKIYHPDKNPD-ESADSA----------
PKNH_1419600 DPFEILEVHAGA--------TVGEIKKAYRLKSLKYHPDKNPNDTSAAAK----------
PKNH_0112400 TYYDILNVKPTA--------TFSEIKSSYYKLALKWHPDKKGDDPEAKVK----------
PKNH_1009500 EFYRILQVPTNA--------SQNEIKRQYYKLAKEYHPDKCSD-SKAKEQ----------
PKNH_1347000 SEWRQVPMSEL-------ISNSTMVKKAYKSAILLCHPDKHRGKPVERVLRA--------
PKNH_1335700 DIYQILGLTFEDAK---KDNIKNIIRKRYLKSALLLHPDKLEASSKG-------------
PKNH_0939900 TSFQILNVNRNC--------SVAEIKQAFSNLSKKWHPDKLGINSSPDIKKRHNNHFKRL
PKNH_0941100 NCYGILGVSEKA--------SVSEIRSSYHRHLMNMKNSYD-------------------
PKNH_1407500 SPFEIFGIHEDV--------DMEKIKSKYRRLSVLIHPDKCKIEKA--------------
PKNH_1436500 NVYEVLSVEEAD--------DLETIKASYKKLILLFHPDKNKGTTFLNQKEKERKRKKER
PKNH_0801600 SLYSHLGVPYNG--------SKEEIKKSYKEKIKVHHPDKGGSV----------------
PKNH_0804300 CFYEILNVESTA--------TVEEIKKSYKKIILQYHPDKNSHLSEE-------------
PKNH_1317800 DYYEVLKCKRSD--------SINKIKKNYRDLSKIYHPDSNKDC----------------
PKNH_0307500 DPYTVLGLSRNA--------TTNEIKKQFRLLAKKYHPDINPSPDAK-------------
PKNH_1207800 DLYEILGVKKNA--------SVKEIAKAYRILVLTYHPDKFAARRGKVVGEKSKTGSEEK
PKNH_1270100 NFYEILNVPRNS--------SKNEIKQAYRKLALKYHPDRN--PNNR-------------
PKNH_1344400 NLYEVLQLNAYA--------SKTDIQQSFRRLSRVYHPDKNKEADS--------------
PKNH_0906300 DYYKRLGIKRNA--------SKEDISKAYRKLAKEYHPDVAPDK----------------
PKNH_1120300 KLYEVLGVHKYA--------TTEEIKKAYRKLSKKYHPDKAKDKNS--------------
PKNH_0424600 KYYEVLNLKKNC--------TTDEVKKAYRKLAIIHHPDKGGDP----------------
PKNH_1311500 NYYEVLGVPQDA--------DLSIIKKSYRTLAMKWHPDKN--PNNK-------------
PKNH_0407900 DYYSILGVSKDC--------TTNDLKKAYRKLAMMWHPDKHKDVKSK-------------
PKNH_0216100 DYYAILGVPKDA--------TENDIKKAYKKLTMKWHPDRHVDPEYK-------------
PKNH_1246700 MYYSVLGVPKDA--------TENDIKKAYKKLAMKWHPDKHLDENDK-------------

PKNH_0718100 ---------------------------------------------------YLNSAYKTL
PKNH_0924200 TSA---------------------------------------------------------
PKNH_1129500 -------------------------------------------------YHCIQKAYQML
PKNH_0319800 -----------------------------------------------------NEAKDVL
PKNH_1031100 ---------------------------------------EKNRTDETSALGIVPIREQNQ
PKNH_1114800 -------------------------------------------------FKQIQEAYQAL
PKNH_0935000 -------------------------------------------------FKKVSIAFQNL
PKNH_0717100 -------------------------------------------------FIKLKQAYDVL
PKNH_1419600 -------------------------------------------------FILITKAYQAL
PKNH_0112400 -------------------------------------------------FQKINEAYQVL
PKNH_1009500 -------------------------------------------------FQKIGEAYQVL
PKNH_1347000 ----------------------------------------------EMIFQALNNAYKEK
PKNH_1335700 ----------------TGPHGTG-------------PQATGTSASNTERFNTLKSAYEFL
PKNH_0939900 FKAKQLLLNDAERMKE-----KKKKETNYVY-----------------------------
PKNH_0941100 ----------------------------------------------LEKKKRILKAYTVL
PKNH_1407500 ----------------------------------------------SEAFHILNKAYEEL
PKNH_1436500 EKGKDGNRNDD-NAKAKGQHGSQETQKDFMYYMEKYNIEKLTPEEKKIMFLKIQDSYAVL
PKNH_0801600 -----------------------------------------------NKFLQLKLSYDIL
PKNH_0804300 -----------------------------------------EQRRCTNIFRQVQEAYECL
PKNH_1317800 -------------------------------------------KDCDKKFRDITKAYKTL
PKNH_0307500 -----------------------------------------------QKMASITAAYELL
PKNH_1207800 ENGKLDVLDKE-TVDE---------------EVNDGEEETLTLEKCKEMFLQIQKAYEIL
PKNH_1270100 -------------------------------------------KESEKMFREITEAYETL
PKNH_1344400 ----------------------------------------------FERFNKIREAYEIL
PKNH_0906300 ----------------------------------------------EKDFIEIANAYETL
PKNH_1120300 ----------------------------------------------NNRFNEIAEAYEIL
PKNH_0424600 -----------------------------------------------EKFKEISRAYEVL
PKNH_1311500 -------------------------------------------AEATEKFKQISEAYEVL
PKNH_0407900 -------------------------------------------KEAEEKFKNIAEAYDVL
PKNH_0216100 -------------------------------------------KIAEEKFKIVLEAYEVL
PKNH_1246700 -------------------------------------------KAAEEKFKIISEAYDVL
```

PKNH_0718100	QNDVDRAL---YLLSI	153
PKNH_0924200	----------------	636
PKNH_1129500	NDKFEKFY---YDVLN	143
PKNH_0319800	LK--------------	115
PKNH_1031100	GDKEEDKNNAKKDNVD	91
PKNH_1114800	YNNDYAQKSH-YGESS	68
PKNH_0935000	INKEKRHE---YDNNS	175
PKNH_0717100	TDDTRRSN---YNRFG	117
PKNH_1419600	TDEISKEN---YEKYG	194
PKNH_0112400	SDSERRAD---YNKYG	345
PKNH_1009500	GDIERRRR---YDKEG	255
PKNH_1347000	KNM-------------	515
PKNH_1335700	MNEQLRNK---YNLYI	103
PKNH_0939900	----------------	1353
PKNH_0941100	ANKRTRKY---YDFFL	95
PKNH_1407500	KKDDIKEQ---YKSVY	180
PKNH_1436500	SDKTLRKQ---YDSSI	297
PKNH_0801600	TNDKKRKM---YDKYG	660
PKNH_0804300	VDERRRKW---YDKNR	78
PKNH_1317800	SDPRLKKA---YDHSK	209
PKNH_0307500	SDPKKKEF---YDKTG	120
PKNH_1207800	RDPEKRKN---YDEYG	116
PKNH_1270100	SDENKKRM---YDSQL	109
PKNH_1344400	SNEKKKYI---YDRFG	119
PKNH_0906300	SDPEKRKM---YDMYG	96
PKNH_1120300	GDEEKRKV---YDHHG	110
PKNH_0424600	SDEEKRKL---YDEYG	88
PKNH_1311500	SDPKRRRK---YDLYG	72
PKNH_0407900	SDEEKRKI---YDAYG	72
PKNH_0216100	SDDYKRRI---YDLYG	153
PKNH_1246700	SDPDKKRT---YDLYG	69

Figure S4: Multiple Sequence Alignment of all PkJDP J Domains except ones with loop insertions

The alignments were created using Clustal Omega (Sievers and Higgins. 2018. Protein Sci. 27: 135-145) and rendered with box shading using Multiple Align Show (Stothard. 2000. BioTechniques. 28: 1102–1104). The residue position of the C-terminal residue of each J domain is shown in the final column.

```
PKNH_0718100  -NFFELFGLQATYD------IDKGHLKQKFNNIQKLYHPDKHAQN---EQ
PKNH_0924200  -DPWKVLQIKKISKMELCDKELKREARKNYRVLALKIHPDKNKSK-----
PKNH_1347000  SEWRQVPMSELIS--------NSTMVKKAYKSAILLCHPDKHRGKPVER-
PKNH_0939900  -TSFQILNVNRNC--------SVAEIKQAFSNLSKKWHPDKLGINSSPDI
PKNH_0941100  -NCYGILGVSEKA--------SVSEIRSSYHRHLMNMKNSYD--------
PKNH_1407500  -SPFEIFGIHEDV--------DMEKIKSKYRRLSVLIHPDKCKI------
PKNH_0801600  -SLYSHLGVPYNG--------SKEEIKKSYKEKIKVHHPDKGGSV-----
PKNH_0804300  -CFYEILNVESTA--------TVEEIKKSYKKIILQYHPDKNSHLS-EEE
PKNH_1317800  -DYYEVLKCKRSD--------SINKIKKNYRDLSKIYHPDSNKDC---K-
PKNH_0307500  -DPYTVLGLSRNA--------TTNEIKKQFRLLAKKYHPDINPSP---DA
PKNH_1270100  -NFYEILNVPRNS--------SKNEIKQAYRKLALKYHPDRN--P---NN
PKNH_1344400  -NLYEVLQLNAYA--------SKTDIQQSFRRLSRVYHPDKNKEA---DS
PKNH_0906300  -DYYKRLGIKRNA--------SKEDISKAYRKLAKEYHPDVAPDK-----
PKNH_1120300  -KLYEVLGVHKYA--------TTEEIKKAYRKLSKKYHPDKAKDK---NS
PKNH_0424600  -KYYEVLNLKKNC--------TTDEVKKAYRKLAIIHHPDKGGDP-----
PKNH_1311500  -NYYEVLGVPQDA--------DLSIIKKSYRTLAMKWHPDKN--P---NN
PKNH_0407900  -DYYSILGVSKDC--------TTNDLKKAYRKLAMMWHPDKHKDV---KS
PKNH_0216100  -DYYAILGVPKDA--------TENDIKKAYKKLTMKWHPDRHVDP---EY
PKNH_1246700  -MYYSVLGVPKDA--------TENDIKKAYKKLAMKWHPDKHLDE---ND
PKNH_1129500  -MYYDILSVRSNA--------DGKTIRRSYLQLSKLFSVHKKLS------
PKNH_0319800  -EAYKILNINPTT--------NRDRIREVHKQLMLKNHPDNGGST-----
PKNH_1114800  -NYHKILGVTKNA--------CKKTIREAYLKKVKLYHPDLNKS------
PKNH_0935000  -NYYEILGIPKNS--------NDETIRAAYKKLAKLYHPDKNKE------
PKNH_0717100  -NYYTYLNITPNA--------TKQEIQTAYRQAAKIYHPDKNPD------
PKNH_1419600  -DPFEILEVHAGA--------TVGEIKKAYRLKSLKYHPDKNPND-----
PKNH_0112400  -TYYDILNVKPTA--------TFSEIKSSYYKLALKWHPDKKGDD-----
PKNH_1009500  -EFYRILQVPTNA--------SQNEIKRQYYKLAKEYHPDKCSD------

PKNH_0718100  LEQINEVSSYLNSAYKTLQNDVDRAL--YLLSI----    153
PKNH_0924200  --NATLAMNILTNSMKAITSA----------------    636
PKNH_1347000  VLRAEMIFQALNNAYKEKKNM----------------    515
PKNH_0939900  KKRHNNHFKRLFKAKQLLLNDAERMKEKKKKETNYVY    1353
PKNH_0941100  ----LEKKKRILKAYTVLANKRTRKY--YDFFL----    95
PKNH_1407500  -EKASEAFHILNKAYEELKKDDIKEQ--YKSVY----    180
PKNH_0801600  -----NKFLQLKLSYDILTNDKKRKM--YDKYG----    660
PKNH_0804300  QRRCTNIFRQVQEAYECLVDERRRKW--YDKNR----    78
PKNH_1317800  --DCDKKFRDITKAYKTLSDPRLKKA--YDHSK----    209
PKNH_0307500  ----KQKMASITAAYELLSDPKKKEF--YDKTG----    120
PKNH_1270100  RKESEKMFREITEAYETLSDENKKRM--YDSQL----    109
PKNH_1344400  ----FERFNKIREAYEILSNEKKKYI--YDRFG----    119
PKNH_0906300  ----EKDFIEIANAYETLSDPEKRKM--YDMYG----    96
PKNH_1120300  ----NNRFNEIAEAYEILGDEEKRKV--YDHHG----    110
PKNH_0424600  -----EKFKEISRAYEVLSDEEKRKL--YDEYG----    88
PKNH_1311500  KAEATEKFKQISEAYEVLSDPKRRRK--YDLYG----    72
PKNH_0407900  KKEAEEKFKNIAEAYDVLSDEEKRKI--YDAYG----    72
PKNH_0216100  KKIAEEKFKIVLEAYEVLSDDYKRRI--YDLYG----    153
PKNH_1246700  KKAAEEKFKIISEAYDVLSDPDKKRT--YDLYG----    69
PKNH_1129500  -REHEECYHCIQKAYQMLNDKFEKFY--YDVLN----    143
PKNH_0319800  -Y----IAAKVNEAKDVLLK-----------------    115
PKNH_1114800  -PDATSKFKQIQEAYQALYNNDYAQKSHYGESS----    68
PKNH_0935000  -KGTEEAFKKVSIAFQNLINKEKRHE--YDNNS----    175
PKNH_0717100  -ESADSAFIKLKQAYDVLTDDTRRSN--YNRFG----    117
PKNH_1419600  -TSAAAKFILITKAYQALTDEISKEN--YEKYG----    194
PKNH_0112400  -PEAKVKFQKINEAYQVLSDSERRAD--YNKYG----    345
PKNH_1009500  -SKAKEQFQKIGEAYQVLGDIERRRR--YDKEG----    255
```

Table S1: *P. knowlesi* JDPs, HSP70s, HSP90s and HOP

Gene ID[1]	Proteomics iRBC[2]	Localization iRBCs[2]	PfJDP Homologs	Type JDP[3]	Comments[4]
JDPs					
**PKNH_0307500**	Y	Parasite/Parasite	PFD0462w / PF3D7_0409400 / Pfj1	I	Homologous to an unusual type I PfJDP with no PEXEL (Pfj1)
**PKNH_0424600**	Y	Parasite/Parasite	PF14_0359 / PF3D7_1437900 / PfHsp40	I	Homologous to a canonical type I PfJDP with no PEXEL (PfHSP40)
**PKNH_0216100**	Y	HCC & Vesicle/Vesicle & HCC	PFE0055c / PF3D7_0501100; PFB0595w / PF3D7_0213100 / PfSis1; PFB0090c / PF3D7_0201800; PFA0660w / PF3D7_0113700	II	Exported? Homologous to two type II PfJDPs, one not exported (PFB0595w) and one exported (PFE0055c); homologous to two other type II PfJDPs, both exported (PFB0090c and PFA0660w); the N-terminal region has a potential PEXEL motif similar to those of PFE0055c, PFB0090c and PFA0660w
**PKNH_0407900**	Y	Parasite/Parasite	PFB0595w / PF3D7_0213100 / PfSis1	II	Homologous to a type II PfJDP with no PEXEL (PFB0595w)
**PKNH_0906300**	Y	Parasite/Parasite	PF11_0099 / PF3D7_1108700 / Pfj2	II	Homologous to a type II PfJDP with no PEXEL (Pfj2); similarly to Pfj2 there is a C-terminal TDEL, suggesting localization to the ER
**PKNH_1114800**	ND	ND	MAL13P1.277 / PF3D7_1356700	II	Homologous to a type II PfJDP with no PEXEL (MAL13P1.277)
**PKNH_1120300**	Y	Parasite/Parasite	PFF1415c / PF3D7_0629200	II	Homologous to a type II PfJDP with no PEXEL (PFF1415c), which has been localized to the PV
**PKNH_1246700**	Y	Vesicle/HCC & Vesicle	PFB0595w / PF3D7_0213100 / PfSis1; PFE0055c / PF3D7_0501100; PFB0090c / PF3D7_0201800; PFA0660w / PF3D7_0113700	II	Exported? Homologous to two type II PfJDPs, one not exported (PFB0595w) and one exported (PFE0055c); homologous to two other type II PfJDPs, both exported (PFB0090c and PFA0660w); since the annotated sequence data for PKNH_1246700 did not appear to encode helices I and II of the J domain, a re-analysis of the genome sequence was conducted using alternative start codons, and this revealed an extended open reading frame encoding additional amino acids at the N-terminus containing helices I and II; this sequence of PKNH_1246700 was used in the protein sequence analyses to determine homologous sequences
**PKNH_1311500**	Y	Parasite/Parasite	PFL0565w / PF3D7_1211400 / Pfj4	II	Homologous to a type II PfJDP with no PEXEL (Pfj4)
**PKNH_1344400**	Y	Parasite/Parasite	PF14_0137 / PF3D7_1413900	II	Homologous to a type II PfJDP with no PEXEL (PF14_0137)
**PKNH_0112400**	Y	Parasite/Parasite	PF08_0115 / PF3D7_0806500; MAL8P1.204 / PF3D7_0831200; PFB0920w / PF3D7_0220100; PF10_0378 / PF3D7_1038800 / Pfj3; PFL0055c / PF3D7_1201100	III	Homologous to a type III PfJDP with no PEXEL (PF08_0115), a type III PfJDP with no PEXEL (MAL8P1.204), a type III PfJDP with a PEXEL (PFB0920w), a type III PfJDP with a PEXEL (PF10_0378), and a type III PfJDP with a PEXEL (PFL0055c); PFB0920w, PF10_0378 and PFL0055c are the three

					exported type III JDPs with PHIST domains and MEC motifs
**PKNH_0319800**	Y	Parasite/Parasite	PF07_0103 / PF3D7_0724400 / PfTIM14	III	Homologous to a type III PfJDP with no PEXEL (PF07_0103/PfTIM14)
**PKNH_0717100**	Y	PVM/Parasite	PFI0935w / PF3D7_0919100	III	Homologous to a type III PfJDP with no PEXEL (PFI0935w)
**PKNH_0718100**	ND	ND	PFI0985c / PF3D7_0920100 / PfJac1	III	Homologous to a type III PfJDP with no PEXEL (PFI0985c)
**PKNH_0801600**	ND	ND	PF10_0032 / PF3D7_1002800	III	Homologous to a type III PfJDP with no PEXEL (PF10_0032)
**PKNH_0804300**	ND	ND	PF10_0057a / PF3D7_1005600 / PfJjj1	III	Homologous to a type III PfJDP with no PEXEL (PF10_0057a)
**PKNH_0924200**	ND	ND	PF11_0273 / PF3D7_1126300	III	Homologous to C-terminus of of a type III JDP with no PEXEL (PF11_0273); J domain near the C-terminus
**PKNH_0935000**	Y	Vesicle/HCC & PVM	PF11_0380 / PF3D7_1136800	III	Exported? Homologous to a type III PfJDP with no PEXEL (PF11_0380)
**PKNH_0939900**	Y	Parasite/Parasite	PF11_0433 / PF3D7_1142100	III	Homologousto a type III PfJDP with no PEXEL (PF11_0433)
**PKNH_1009500**	Y	PVM & Parasite/Parasite	PFE1170w / PF3D7_0523400	III	Homologous to a type III PfJDP with no PEXEL (PFE1170w)
**PKNH_1031100**	ND	ND	PFE0135w / PF3D7_0502800 / PfJii3	III	Homologous to a type III PfJDP with no PEXEL (PFE0135w); appears to have an insertion in the J domain leading to a long loop region after the HPD
**PKNH_1207800**	Y	Parasite/Parasite	PF14_0700 / PF3D7_1473200	III	Homologous to a type III PfJDP with no PEXEL (PF14_0700); appears to have an insertion in the J domain leading to a long loop region after the HPD
**PKNH_1270100**	ND	ND	MAL13P1.162 / PF3D7_1330300	III	Homologous to a type III PfJDP with no PEXEL (PFE1170w)
**PKNH_1317800**	Y	Parasite & PVM/Parasite	PF08_0032 / PF3D7_0823800	III	Homologous to a type III PfJDP with no PEXEL (MAL13P1.162)
**PKNH_1335700**	Y	Parasite/Parasite	PF14_0213 / PF3D7_1422300	III	Homologous to a type III PfJDP with no PEXEL (PF14_0213); appears to have an insertion in the J domain leading to a long loop region after the HPD
**PKNH_1347000**	Y	Parasite/Parasite	PF14_0111 / PF3D7_1411300	III	Homologous to a type III PfJDP with no PEXEL (PF14_0111); has a J domain near the C terminus
**PKNH_1407500**	Y	Parasite/Parasite	PF13_0036 / PF3D7_1307200	III	Homologous to a type III PfJDP with no PEXEL (PF13_0036)
**PKNH_1419600**	Y	PVM/PV	PF13_0102 / PF3D7_1318800 / PfSec63	III	Homologous to a type III PfJDP with no PEXEL (PF13_0102 / PfSec63), which is a homolog of human Sec63
**PKNH_1436500**	Y	Parasite/Parasite	PFL0815w / PF3D7_1216900 / PfZuo1	III	Homologous to a Pf type III JDP with no PEXEL (PFL0815w); appears to have an insertion in the J domain leading to a long loop region after the HPD
**PKNH_0941100**	Y	Parasite/Parasite	PF11_0443 / PF3D7_1143200	IV	Homologous to a type IV PfJDP with no PEXEL (PF11_0443); J domain has a corrupted HPD (KNS)
**PKNH_1129500**	Y	Parasite/Parasite	PFF1010c / PF3D7_0620700	IV	Homologous to a type IV PfJDP (PFF1010c); J domain has a corrupted HPD (SVH)

**HSP70s**					
PKNH_1312700	Y	Parasite/Parasite	PF08_0054 / PF3D7_0818900 / PfHSP70-1	NA	Homologous to the highly abundant, intracellular and cytosolic/nuclear PfHSP70-1
PKNH_0715900	Y	Parasite/Parasite	PFI0875w / PF3D7_0917900 / PfHSP70-2 / PfBiP	NA	Homologous to the ER-localized PfHSP70-2
PKNH_0932200	Y	Parasite/Parasite	PF11_0351 / PF3D7_1134000 / PfHSP70-3	NA	Homologous to the mitochondrial-localized PfHSP70-3
PKNH_1257200	Y	Parasite/Parasite	MAL13P1.540 / PF3D7_1344200 / PfHSP70-y	NA	Homologous to the ER-localized PfHSP70-y
PKNH_0107400	Y	Parasite/Parasite	PF07_0033 / PF3D7_0708800 / PfHSP70-z/ PfHSP110c	NA	Homologous to the cytosol-localized PfHSP70-z
**HSP90s**					
PKNH_0107000	Y	Parasite/Parasite	PF3D7_0708400 / PfHSP90	NA	Homologous to the highly abundant, intracellular and cytosolic PfHSP90
PKNH_1441400	Y	Parasite/Parasite	PF3D7_1222300 / PfGRP94	NA	Homologous to the ER-localized PfGRP94
PKNH_0915900	Y	Parasite/Parasite	PF3D7_ 1118200 / PfTRAP1 / PfHSP90_M	NA	Homologous to the mitochondrial-localized PfTRAP1
PKNH_1238400	Y	Parasite/Parasite	PF3D7_1443900 / PfHSP90_A	NA	Homologous to the apicoplast-localized PfHSP90_A
**HOP**					
PKNH_0420900	Y	Parasite/Parasite	PF3D7_1434300 / PfHOP	NA	Homologous to the cytosol-localized PfHOP

[1]Oresegun et al. (2022. Front. Genet. 13:855052)
[2]Detection and localization in fractions of infected human red blood cells (iRBCs) subjected to proteomics analyses; localization was determined by two algorithms with up to two possible locations for each approach, first most probable and second most probable; the first prediction methodology, “used an ‘iterative Permutation’ (P) algorithm”, and the second prediction methodology “used a parallel ensemble ‘Machine Learning’ (ML) approach”; ND = Not Determined/Identified; HCC = host cell cytosol; Siau et al. (2023. Cell Reports. 42: 113419)
[3]The JDPs were categorized by type; see main text for the definitions of types I, II, III and IV; Botha et al. (2007. Int. J. Biochem. Cell Biol. 39: 1781-1803); Dutta et al. (2021. Adv. Exp. Med. Biol. 1340: 97-123) (NA = Not Applicable)
[4]JDP comments, Dutta et al. (2021. Adv. Exp. Med. Biol. 1340: 97-123); HSP70 comments, Blatch (2022. Front. Cell Dev. Biol. 10: 921739); HSP90 comments, Dutta et al. (2022. Biomolecules. 12: 1018); potential PfJDP homologs were identified on the basis of phylogenetic analyses; the Supplementary Materials contains the details of the analyses, including the sequences used (List S1) and the phylogenetic trees (Figure S1A and Figure S1B)
